# It is all about the solvent: on the importance of the mobile phase for ZIC-HILIC glycopeptide enrichment

**DOI:** 10.1007/s00216-016-0051-6

**Published:** 2016-12-01

**Authors:** Kathirvel Alagesan, Sana Khan Khilji, Daniel Kolarich

**Affiliations:** 1 Department of Biomolecular Sciences, Max Planck Institute of Colloids and Interfaces, 14424 Potsdam, Germany; 2 Institute of Chemistry and Biochemistry, Freie Universität Berlin, 14195 Berlin, Germany

**Keywords:** Glycoproteomics, Glycopeptide enrichment, Hydrophilic interaction chromatography, HILIC, Glycopeptide synthesis

## Abstract

Glycopeptide enrichment is a crucial step in glycoproteomics for which hydrophilic interaction chromatography (HILIC) has extensively been applied due to its low bias towards different glycan types. A systematic evaluation of applicable HILIC mobile phases on glycopeptide enrichment efficiency and selectivity is, to date, however, still lacking. Here, we present a novel, simplified technique for HILIC enrichment termed “Drop-HILIC”, which was applied to systematically evaluate the mobile phase effect on ZIC-HILIC (zwitterionic type of hydrophilic interaction chromatography) glycopeptide enrichment. The four most commonly used MS compatible organic solvents were investigated: (i) acetonitrile, (ii) methanol, (iii) ethanol and (iv) isopropanol. Glycopeptide enrichment efficiencies were evaluated for each solvent system using samples of increasing complexity ranging from well-defined synthetic glycopeptides spiked into different concentrations of tryptic BSA peptides, followed by standard glycoproteins, and a complex sample derived from human (depleted and non-depleted) serum. ZIC-HILIC glycopeptide efficiency largely relied upon the used solvent. Different organic mobile phases enriched distinct glycopeptide subsets in a peptide backbone hydrophilicity-dependant manner. Acetonitrile provided the best compromise for the retention of both hydrophilic and hydrophobic glycopeptides, whereas methanol was confirmed to be unsuitable for this purpose. The enrichment efficiency of ethanol and isopropanol towards highly hydrophobic glycopeptides was compromised as considerable co-enrichment of unmodified peptides occurred, though for some hydrophobic glycopeptides isopropanol showed the best enrichment properties. This study shows that even minor differences in the peptide backbone and solvent do significantly influence HILIC glycopeptide enrichment and need to be carefully considered when employed for glycopeptide enrichment.

**Electronic supplementary material:**

The online version of this article (doi:10.1007/s00216-016-0051-6) contains supplementary material, which is available to authorized users.

**Graphical Abstract:**
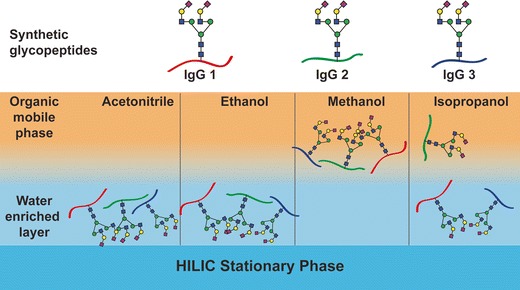
The organic solvent plays a crucial role in ZIC-HILIC glycopeptide enrichment

## Introduction

Protein glycosylation is one of the most common and functionally diverse post-translational modifications. It is involved in different processes such as cell–cell interaction and signal recognition [1] and is an essential regulating factor of the immune system [2–4]. Glycosylation is a non-template-driven enzymatic process that also reflects the physiological state of the cell [5, 6], and glycoprotein functions are dictated by both glycans and their respective proteins. Thus, sensitive and selective methods for primary structure sequencing of glycoproteins are essential to understand and study the functional significance of glycosylation. This integrated and systematic approach for compiling glycoprotein structure and function is one major aim in glycoproteomics [7].

Peptide and glycopeptide mixtures are frequently analysed following proteolytic digestion by either LC-ESI-MS/MS or MALDI-TOF-MS. However, simultaneous detection of peptides and glycopeptides can be tricky. Glycoprotein proteolysis often results in unequal mixtures of these compounds as glycopeptide microheterogeneity reduces the concentration of each individual glycopeptide molecule compared to unmodified peptides obtained by the same digest [8]. Hydrophobic molecules also tend to provide stronger signals compared to hydrophilic ones, which further complicates glycopeptide detection in the presence of unmodified peptides [9]. Subsequently, glycopeptide signal strengths are significantly lower compared to their unmodified counterparts, mostly due to the presence of the large hydrophilic glycan moiety [10]. Therefore, glycopeptide enrichment is often performed to allow their detection and identification [11–13].

In contrast to glycopeptide enrichment methods using lectins, hydrazide chemistry, titanium dioxide or graphitized carbon, hydrophilic interaction chromatography (HILIC) comes with the unique advantage to enable glycopeptide enrichment in a largely glycan structure unbiased manner. During the HILIC enrichment process, glycopeptides are also not chemically or enzymatically altered: This is highly relevant for in-depth glycoproteomics. Another significant advantage of HILIC is that both peptide and glycan present in the enriched fraction can be analysed in a high-throughput fashion as intact glycopeptides but also individually after enzymatic treatments with PNGase F/A.

In contrast to normal phase chromatography, the HILIC retention mechanism is largely a result of a hydrophilic partitioning of the analyte to the water-enriched layer surrounding the polar stationary phase [14]. Glycopeptide retention mainly depends on the size of the glycan moiety and its hydrophilic properties, but also on the hydrophilic features of the peptide backbone. The polar interaction between the glycopeptides’ glycan moieties with the hydrophilic layer surrounding the stationary phase provides an opportunity to separate glycopeptides from (usually) less hydrophilic peptides. Furthermore, depending upon the type of the used stationary phase, hydrogen bonding, electrostatic or dipole–dipole interactions also influence analyte retention [14, 15]. A wide range of HILIC stationary phases have successfully been reported for glycopeptide enrichment ranging from silica particles [16], cellulose [17], sulfoalkylbetaine (ZIC-HILIC) [18, 19], amide-based [20] to even simple cotton [21]. The excellent review by Hemström and Irgum describes in detail the developments in polar stationary phases and their retention mechanisms [22]. More recently, also the suitability of several chemically fabricated stationary phases has been reported for glycopeptide enrichment [23–25].

Glycopeptide enrichment is usually performed at starting conditions with 80% organic solvent concentration while elution is performed by disturbing the hydrophilic interactions using aqueous conditions. Acetonitrile is by far the most popular organic mobile phase applied for this purpose [26]. Although HILIC SPE is efficient (reproducible and sensitive) for glycopeptide enrichment from mixtures, hydrophilic non-glycosylated peptides are also frequently co-enriched. This represents a particular problem for analysing complex samples as the co-enriched hydrophilic peptides can cause glycopeptide ion-suppression. Co-enrichment of hydrophilic non-glycosylated peptides can, however, be avoided or at least significantly reduced by the addition of suitable ion-pairing reagents such as trifluoroacetic acid (TFA) or hydrochloric acid (HCl) [27, 28]. Alternatively, the reduction of non-specific enrichment has also been reported by digesting the glycoprotein with non-specific proteases prior HILIC enrichment. This greatly increases glycopeptide hydrophilicity due to the shorter peptide backbone [29]. The drawbacks of this approach are, however, increased sample heterogeneity, impeded accurate site specific glycan structure assignment and lack of accurate relative quantitation of site specific microheterogeneity [30].

Andrew Alpert once described HILIC as “the combination of hydrophilic stationary phases and hydrophobic, mostly organic mobile phases” [14]. Compared to other HILIC materials, ZIC-HILC is known to provide higher selectivity for glycopeptides [31]. Therefore, in the present work, we systematically evaluated the effect various mobile phases [acetonitrile (ACN), methanol (MeOH), ethanol (EtOH) and isopropanol (IPA)] have on the selectivity and efficiency to enrich glycopeptides using ZIC-HILIC. Glycopeptide enrichment efficiencies were evaluated for each solvent system using a variety of samples, which required the development of an enrichment technique suitable for this purpose termed “Drop-HILIC”. Drop-HILIC is significantly cheaper and quicker to perform than the conventional micro-spin technique and provides comparable results. Different purified glycoproteins as well as more complex samples provided in the form of depleted and non-depleted human serum were tested to conclude that glycopeptide enrichment efficiency largely depends on the organic mobile phase.

## Materials and methods

### Materials

If not otherwise stated, all materials were purchased in the highest possible quality from Sigma-Aldrich (St. Louis, MO, USA). Trypsin (sequencing grade) was obtained from Roche Diagnostic GmbH (Mannheim, Germany). Water was used after purification with a Milli Q-8 direct system (Merck KGaA, Darmstadt, Germany). IgG (I4506) and A1PI (A6150) were obtained from Sigma-Aldrich. Human serum was obtained from BioreclamationIVT (New York, USA). The amino acid numbering applied for all proteins analysed in this study is based on the respective UniProtKB entries.

### High abundance serum protein depletion

Depletion of abundant serum proteins was performed according to the manufactures instructions using a commercially available kit (ProteoSpin™ Abundant Serum Protein Depletion Kit Cat. # 17300).

### In-solution protease digestion

One hundred micrograms of protein [IgG, A1PI or serum (depleted and non-depleted)] was reduced with 10 μL of 500 mM dithiothreitol (DTT) (in H_2_O) (99 °C, 5 min) and then subsequently alkylated with 10 μL 500 mM iodoacetamide (IAA) solution (in H_2_O) at room temperature for 60 min in the dark. Prior trypsin digestion, the samples were subjected to chloroform-methanol precipitation as described earlier [32]. The protein pellet was resolubilised in 200 μL of 25 mM NH_4_HCO_3_ and trypsin added in a 1:30 ratio (enzyme:substrate). After overnight incubation at 37 °C, the resulting glycopeptide/peptide mixtures were aliquoted corresponding to 3 μg of initial protein concentration and dried in the speedvac without additional heating. The samples were stored at −25 °C until further experiments.

### HILIC enrichment—micro-spin

ZIC-HILIC (pore size 200 Å, 10 μm particle size, SeQuant AB, Sweden) was filled up to 1.5 cm in a C18 ZipTip P10 (Merck Millipore, Tullagreen, IRL). The column was washed three times with 50 μL of 1% TFA and then equilibrated three times with 50 μL of 80% ACN containing 1% TFA. The dried sample was reconstituted in 10 μL 1% TFA and slowly adjusted to 80% ACN/1% TFA by the addition of 40 μL ACN/1% TFA. The sample was applied onto the column and centrifuged until the entire liquid passed through. The flowthrough was reapplied onto the column and again centrifuged. The sample was washed twice with 50 μL of 80% ACN containing 1% TFA and glycopeptides were eluted off the column by washing it thrice with 50 μL of 1% TFA followed by 50 μL of 80% ACN containing 1% TFA. The eluted fraction was dried in the speedvac and reconstituted in 50 μL of 0.1% TFA for further MS analyses. All analyses were performed in triplicate.

### HILIC enrichment—“Drop-HILIC”

Tryptic protein digests were dissolved in 10 μL 1% TFA and slowly adjusted to 80% organic solvent conditions by the addition of 40 μL organic solvent (ACN/1% TFA or EtOH/1% TFA or MeOH/1% TFA or IPA/1% TFA). ZIC-HILIC beads were washed three times with 1% TFA (3× 250 μL) and then equilibrated three times with appropriate binding solution (3× 250 μL). Subsequently, the HILIC beads were added to the sample and incubated at room temperature for 1, 3 or 5 min with occasional shaking. After incubation, the HILIC beads were spun down in a table centrifuge. The supernatants (flowthrough) were transferred into a new vial. The HILIC beads were then mixed with 50 μL appropriate binding solution, vortexed and spun down. The supernatants were pooled together, and the washing step was repeated twice. Enriched glycopeptides were eluted using 3× 50 μL of the elution buffer (1% TFA), and all three elution supernatants were combined in a new vial. The eluate was dried in the speedvac and reconstituted in 50 μL of 0.1% TFA for further MS analyses. All analyses were performed in triplicate.

### Glycopeptide synthesis

Solid phase glycopeptide synthesis (SPGPS) was performed manually using 5 and 10-mL disposable polypropylene syringes with a bottom filter. All peptides and glycopeptides were synthesised by SPGPS using previously reported fluorenylmethoxycarbonyl (Fmoc) protocols [10, 33, 34] and as described in detail in the Electronic Supplementary Material (ESM).

### LC-MS analysis parameters

Nano-LC-ESI-MS analysis was carried out on an Ultimate 3000 RSLC-nano system (Dionex/Thermo Scientific, Sunnyvale, CA) coupled to an amaZon speed ETD ion trap mass spectrometer (IT-MS) equipped with CaptiveSpray nanoBooster™ (both Bruker, Bremen, Germany). In each run, glycopeptides corresponding to 180 ng were injected.

In nano-LC mode, the peptides were concentrated on a C18 precolumn (Acclaim PepMap100™, Thermo, 100 μm × 20 mm, 5 μm particle size) and separated by reversed phase chromatography on a C18 analytical column (Acclaim PepMap™, Thermo, 75 μm × 15 cm, 3 μm particle size). The samples were loaded in 99% loading buffer (0.1% TFA) for 5 min on the precolumn at a flow rate of 5 μL/min before the captured peptides were subjected to reversed phase nanoLC at a flowrate of 400 nL/min on a column equilibrated in 95% buffer A (0.1% formic acid). The gradient conditions were as follows: increase of buffer B (90% acetonitrile containing 0.1% formic acid) from 5 to 45% (6–36 min), further increase to 70% B (36–38 min), followed by a steeper increase to 90% B (40–42 min). The column was held at 90% B for 10 min (42–52 min). The mass spectrometer was set up to perform CID on the three most intense signals in every MS scan. An *m/z* range from 400 to 1600 Da was used for data-dependent precursor scanning. The MS data was recorded using the instrument’s “enhanced resolution mode”. MS/MS data was acquired in “ultra-mode” over an *m/z* range from 100 to 2000. A detailed parameter setting is provided in ESM Table S1 following MIRAGE [35] and MIPAE [36] recommendations.

Data analysis was performed using ProteinScape 3 (Bruker Daltonics) and MASCOT 2.3 (MatrixScience, United Kingdom) using the following search parameters: Cysteine as carbamidomethyl was set as fixed modification, and deamidation (Asn/Gln) and oxidation (Met) were set as variable modifications. Up to two missed cleavages were allowed. Peptide tolerance was set at ±0.3 Da for MS and at ±0.5 Da for MS/MS. The data were searched against the SwissProt protein database (taxonomy restriction: *Homo sapiens*, SwissProt 2011_08; 531,473 sequences; 188,463,640 residues).

## Results and discussion

### Rationale and experiment design

**Fig. 1:**
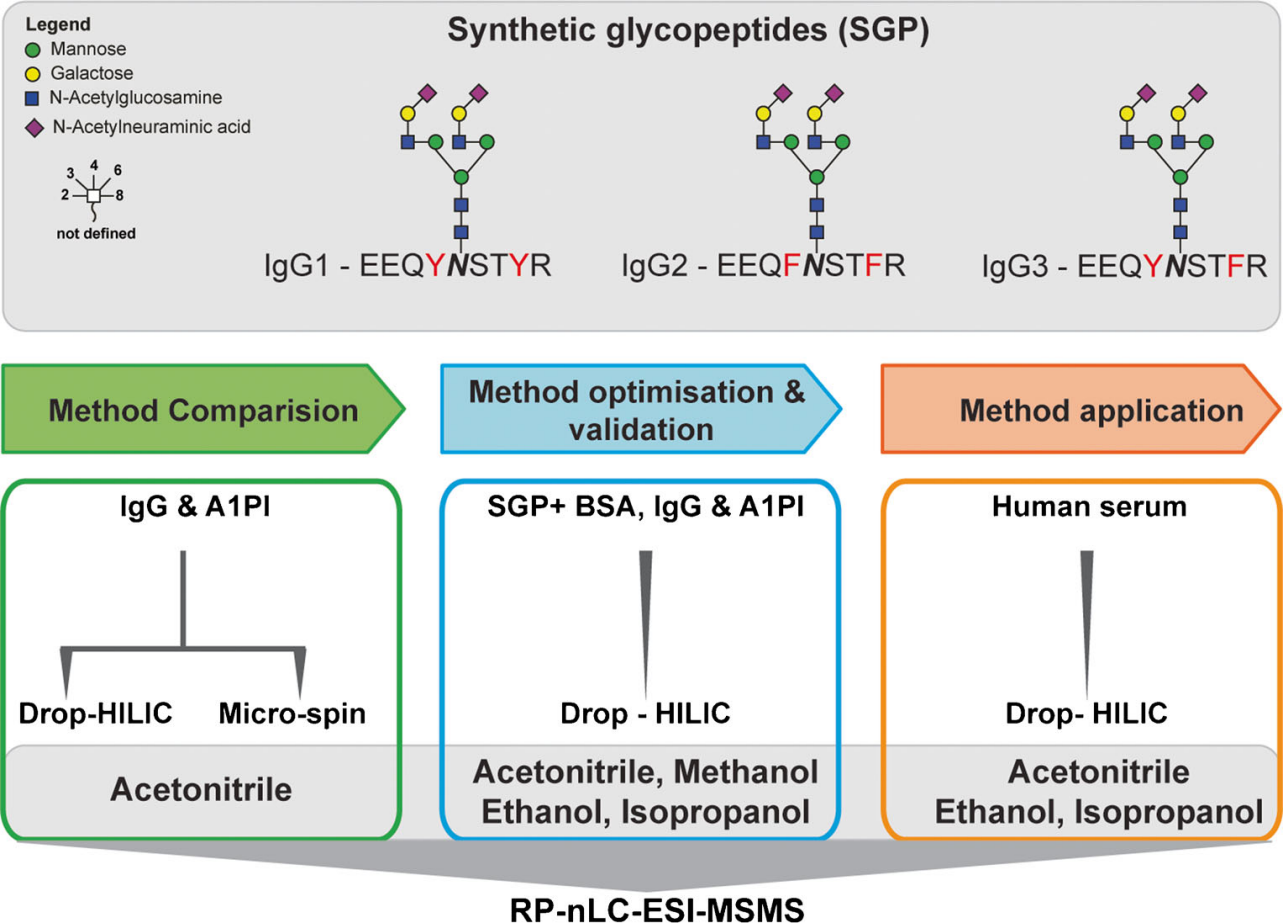
Experiment design. Investigating the influence of various organic mobile phases on ZIC-HILIC glycopeptide enrichment efficiency and development of a simplified HILIC enrichment technique (“Drop-HILIC”). All four mobile phases [(i) acetonitrile, (ii) ethanol, (iii) methanol and (iv) isopropanol] were tested using 80% organic solvent starting conditions. Glycopeptide enrichment efficiencies were evaluated by nanoLC ESI-MS/MS using samples of increasing complexity ranging from well-defined synthetic glycopeptides spiked into a tryptic digest of BSA over individual standard glycoproteins to a tryptic digest of depleted and non-depleted human serum

The efficiency and selectivity of glycopeptide enrichment by ZIC-HILIC SPE were first investigated using well-defined synthetic glycopeptides spiked into different concentrations of tryptic peptides derived from BSA. Following these initial experiments, HILIC was performed on proteolytic digests of standard glycoproteins (IgG and A1PI) and finally applied on a complex sample derived from human serum (Fig. 1). The influence of the mobile phase on glycopeptide enrichment efficiency was tested using four different solvents: (i) acetonitrile, (ii) methanol, (iii) ethanol and (iv) isopropanol were used at a concentration of 80% in water containing 1% TFA as an acidic modifier. In the course of this work, we also evaluated a simplified HILIC enrichment technique (“Drop-HILIC”) and compared it to the traditional micro-spin HILIC approach.

### Salt removal is crucial for efficient HILIC enrichment

Initial experiments applying the micro-spin method to enrich IgG glycopeptides resulted in no/insufficient enrichment when performed subsequently following in-solution trypsin digestion (data not shown). Applying this workflow directly on in-gel digested (glyco)peptides, however, provided the expected results. We found that the residual presence of higher salt concentrations derived from the alkylating reagents resulted in electrostatic (ionic) interactions that were compromising glycopeptide enrichment when performed subsequently after an in-solution proteolytic digest. A simple reversed phase-based desalting step could be introduced prior HILIC. This step, however, can also lead to the loss of very hydrophilic glycopeptides [37]. To avoid such losses, we modified our in-solution sample preparation protocol by introducing a simple chloroform–methanol precipitation step immediately after reduction and alkylation to remove any excess DTT and IAA. This simple, fast and efficient step allowed us to successfully enrich glycopeptides, greatly minimise sample losses and eliminate any molecules affecting the enrichment efficiency. These optimised proteolytic sample preparation conditions were then applied for all following experiments.

### Same, same but different: to spin or to “Drop-HILIC”

Using acetonitrile loading solvent conditions in our hands, the sample required approximately 3–5 min to pass through the ZIC-HILIC micro-spin column. However, when applying more viscous solvents such as isopropanol up to 20 min were required. As we intended to evaluate the influence of various organic mobile phases on the glycopeptide enrichment, we established a simplified and accelerated technique by simply co-incubating the sample with the HILIC beads, termed “Drop-HILIC”. This approach provided the opportunity to normalise incubation times and evaluate any solvent effect, which could not reasonably be achieved by the micro-spin HILIC method. Besides being easier and quicker to perform, Drop-HILIC provided additional advantages: (i) the amount of HILIC material could be optimised to accommodate variable sample amounts and (ii) it was more cost and time effective as no C18 Zip-tip columns or custom made tips were required.

**Fig. 2:**
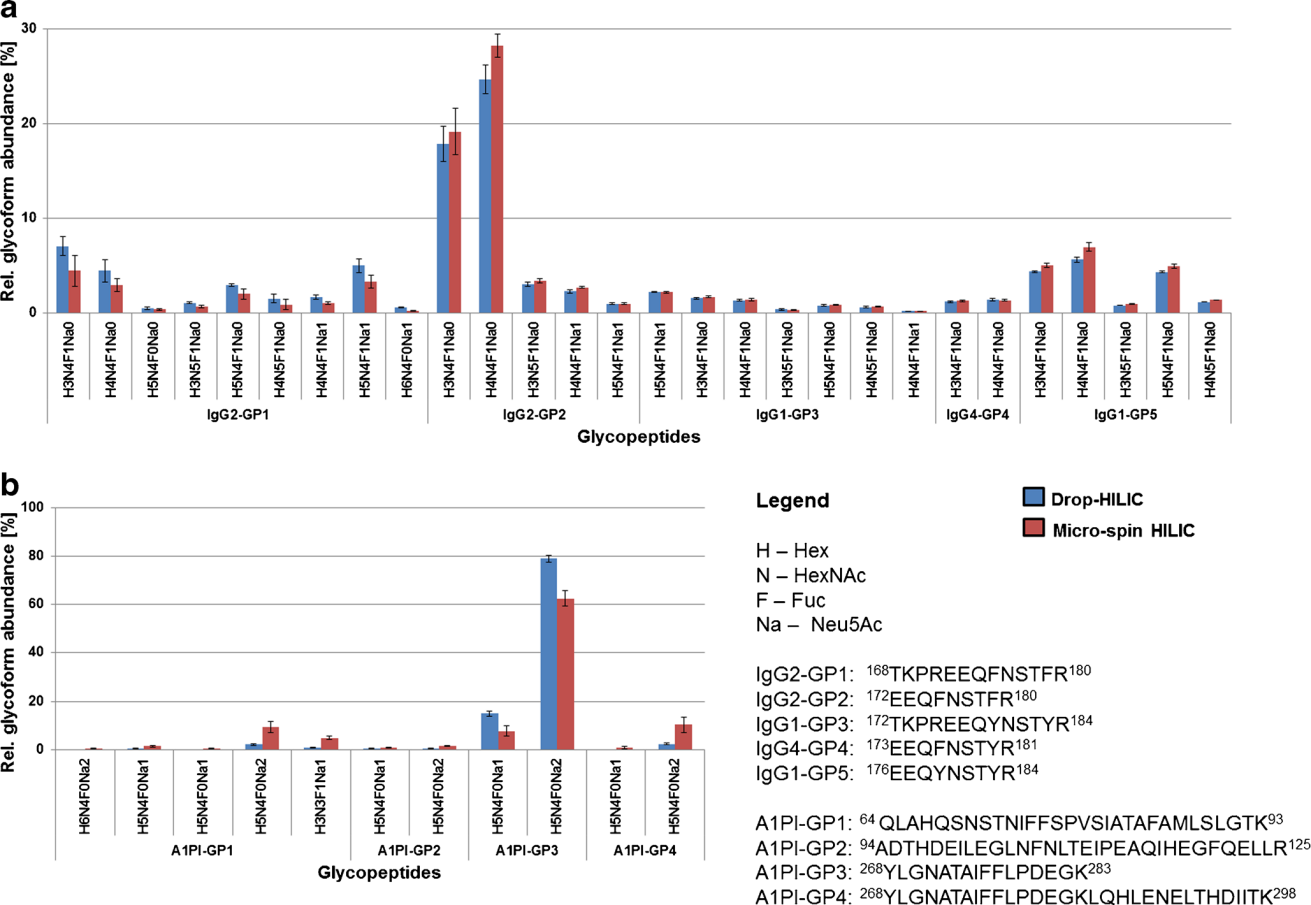
Glycoprofile comparison of the Drop-HILIC and micro-spin techniques using ACN as mobile phase for glycopeptide enrichment from (**a**) IgG and (**b**) A1PI. Enriched glycopeptides were analyzed by RP-nanoLC-ESI-MSMS. IgG contains a single site of glycosylation whereas three are present in A1PI. The relative abundances were determined using the area under the curve of extracted ion chromatograms (EIC’s) produced from all glycoform and charge state signals detected for each single glycopeptide. Three technical replicates were performed. Both techniques performed similar on IgG glycopeptides, while some glycoprofile differences were found for the hydrophobic A1PI glycopeptides

We first compared the micro-spin and Drop-HILIC approaches using two well-characterised standard glycoproteins, human IgG and A1PI using 80% acetonitrile loading solvent conditions. The glycopeptide enriched fractions were subsequently analysed by nanoLC-ESI-MSMS (Fig. 2, ESM Tables S2 and S3). Both techniques yielded comparable IgG glycopeptide profiles (Fig. 2a). IgG2 provided the most abundant signals followed by the glycopeptides derived from IgG1 and IgG4. However, a slightly different trend was observed in the case of A1PI. The drop approach enriched glycopeptides A1PI-GP3 (^268^YLG**N**ATAIFFLPDEGK^283^) significantly better. On the other hand, glycopeptides containing a larger peptide backbone (A1PI-GP4 carrying H5N4F0Na2 and H3N3F0Na1 as well as A1PI-GP1) were not enriched with similar efficiency (Fig. 2b). In order to evaluate whether the one-minute incubation time affects Drop-HILIC enrichment efficiency for these larger glycopeptides, possibly due to inadequate phase partitioning, we additionally tested various incubation times.

### Incubation Time Does Not Influence Drop-HILIC Enrichment Efficiency

Various incubation times (1, 3 and 5 min) did not show any significant changes in the enrichment efficiency of IgG glycopeptides under standard conditions. Longer incubation times did also not improve the enrichment of the larger hydrophobic A1PI glycopeptides, indicating that the Drop-HILIC approach exhibited some limitations for efficiently enriching such large, >25 amino acid long, comparably hydrophobic glycopeptides. Despite these observed limitations the Drop-HILIC approach provided glyco-profile results comparable to the classical micro-spin HILIC method for IgG and A1PI-GP3 (ESM Fig. S1). Drop-HILIC, however, came with the advantages of being significantly quicker and cheaper to perform. As the incubation time did not show any influence on the enrichment efficiency all further experiments were performed using 1 min incubation times for the evaluation of any effect the organic mobile phase has on glycopeptide enrichment efficiency.

### It is all about the solvent—influence of the solvent system on ZIC-HILIC glycopeptide enrichment

The underlying mechanism of analyte retention in ZIC-HILIC is originating from hydrophilic partitioning in addition to contributions derived from minor electrostatic interactions. The use of TFA in the mobile phase nullifies any possible electrostatic interactions making hydrophilic partitioning the only cause for analyte retention [28]. For an efficient enrichment, the ideal mobile phase should be water miscible, but not contribute any hydrogen donor or acceptor functionalities. A completely aprotic solvent such as acetonitrile embraces this particular feature and thus is often used for glycopeptide enrichment by HILIC SPE. However, a systematic evaluation of any mobile phase effect on glycopeptide enrichment is still lacking. The optimised sample preparation step in combination with our simple, fast and equally efficient Drop-HILIC approach allowed us to evaluate the influence of various MS compatible mobile phase solvents on ZIC-HILIC glycopeptide enrichment efficiency.

First, we evaluated the solvent effect in a defined system using glycoform-specific synthetic *N*-glycopeptides spiked into the background of tryptically digested BSA (please refer ESM – Glycopeptide synthesis section). The synthetic glycopeptides corresponded to the tryptic glycopeptide sequences present in IgG1, IgG2 and IgG3 and carried a bianntenary, disialylated *N*-glycan (Fig. 1). These compounds were mixed with tryptic BSA derived peptides in molar ratios 3:1:1:3 (IgG1/IgG2/IgG3/BSA), and glycopeptide enrichment was performed using the Drop-HILIC technique. Our initial results indicated that methanol is a non-favoured mobile phase for this purpose (data not shown). Due to its strong tendencies to form hydrogen bonds, methanol effectively competes for the active sites on the stationary phase and is thereby perturbing hydrophilic partitioning. This subsequently resulted in strongly reduced glycopeptide retention and thus was not further evaluated [38]. The glycopeptide-enriched fractions from the other solvents (ACN, EtOH, IPA), however, were analysed by RP-nano LC-ESI-IT-MSMS.

**Fig. 3:**
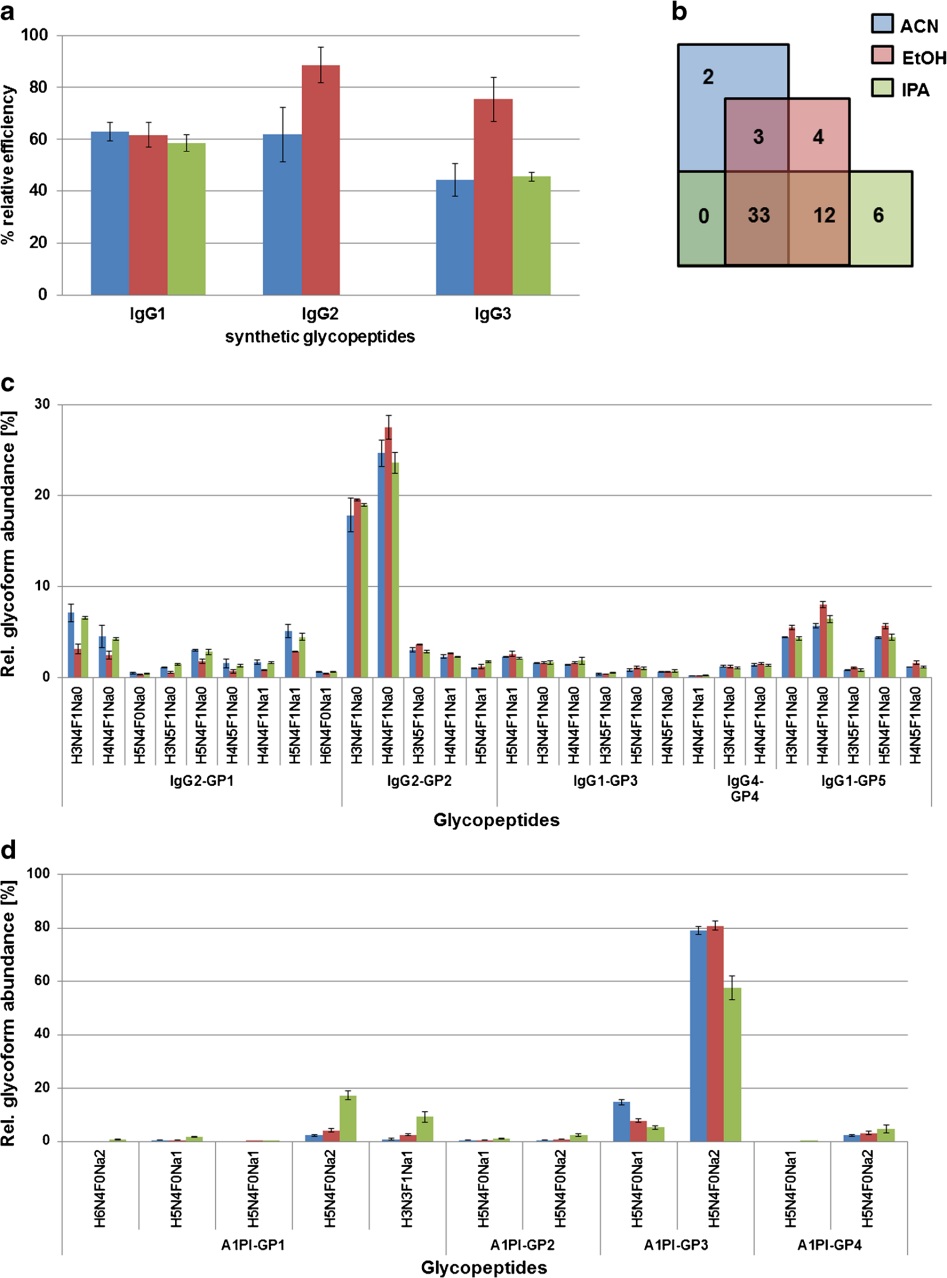
Loading solvent effect on ZIC-HILIC glycopeptide enrichment. **a** Synthetic *N*-glycopeptides corresponding to IgG1, IgG2 and IgG3 tryptic peptides were spiked into a tryptic digest of BSA. Whereas ACN did effectively enrich all three isoforms, EtOH and IPA exhibited IgG subclass specific tendencies. **b** Venn diagram showing the number of BSA-derived peptides co-enriched with the synthetic glycopeptides when using different loading solvents. **c**, **d** Different mobile phases were used for loading the (glyco)peptide mixtures onto the resin, resulting in a differential enrichment of various glycopeptide species from (**c**) IgG and (**d**) A1PI samples

We observed a mobile phase solvent dependency in the selectivity and efficiency for glycopeptide enrichment (Fig. 3a), which was also considerably influenced by the hydrophilicity of the peptide backbone (ESM Table S2). The synthetic IgG1 glycopeptide was efficiently enriched in a similar manner by all three solvents, while a strong mobile phase dependency was observed for IgG2 and IgG3 synthetic glycopeptides. Ethanol significantly enriched the synthetic IgG3 (75.43 ± 8.42%) and IgG2 (88.58 ± 6.76%) glycopeptides better than ACN or IPA while the IgG2 glycopeptide could not be enriched at all using IPA in the background of tryptic BSA peptides (Fig. 3a). Interestingly, this peptide is also the most hydrophobic of the three synthetic *N*-glycopeptides (GRAVY score of −1.60, see also ESM Table S4). The data suggested that either the hydrophilic BSA peptides outcompeted the IgG2 synthetic *N*-glycopeptide or suppressed its ionisation, making it not detectable under the used conditions. When excess molar ratios of BSA were applied (IgG1/IgG2/IgG3/BSA = 3:1:1:6 and 3:1:1:10), glycopeptide enrichment efficiency was compromised especially in the case of EtOH and IPA as the abundances of co-enriched peptides clearly increased (ESM Fig. S4). Under the tested conditions, ACN provided the best compromise for the retention of all three synthetic glycopeptides while keeping the number of co-enriched BSA peptides at a low level. Nevertheless, with all three mobile phases, numerous peptides were co-enriched in a mobile phase dependent manner (Fig. 3b, ESM Table S5).

Inspired from these results, we next studied the loading solvent influence on ZIC-HILIC glycopeptide enrichment using individual standard glycoprotein digests of IgG and A1PI. Also for these low-complex samples, a loading solvent-dependant selectivity was found. The human IgG glycopeptides showed similar results as obtained for the synthetic ones, with the exception that IPA was a suitable solvent to enrich the IgG2 glycopeptide (Fig. 3c), indicating that the BSA tryptic peptide background was interfering with its enrichment in earlier experiments (Fig. 2a). In the case of A1PI, however, isopropanol provided the best compromise for the simultaneous enrichment of hydrophilic and hydrophobic glycopeptides (Fig. 3d). This can possibly be explained by the fact that not all (glyco)peptides were equally soluble under 80% organic mobile phase conditions [39]. This hypothesis was also supported by the different identified co-enriched unmodified peptides that were found for the individual solvents (ESM Table S6A- S6C, Fig. S5). As a consequence of this insolubility, an insufficient enrichment of certain glycopeptide species was observed when using EtOH. Our results indicated that analyte retention in HILIC was not just controlled by hydrophilic partition but that more complex mechanisms occurring prior sample loading and at the interface of the stationary polar and organic mobile phase during sample solvation were also influencing enrichment efficiency.

### Glycopeptide enrichment from human serum using Drop-HILIC

**Fig. 4:**
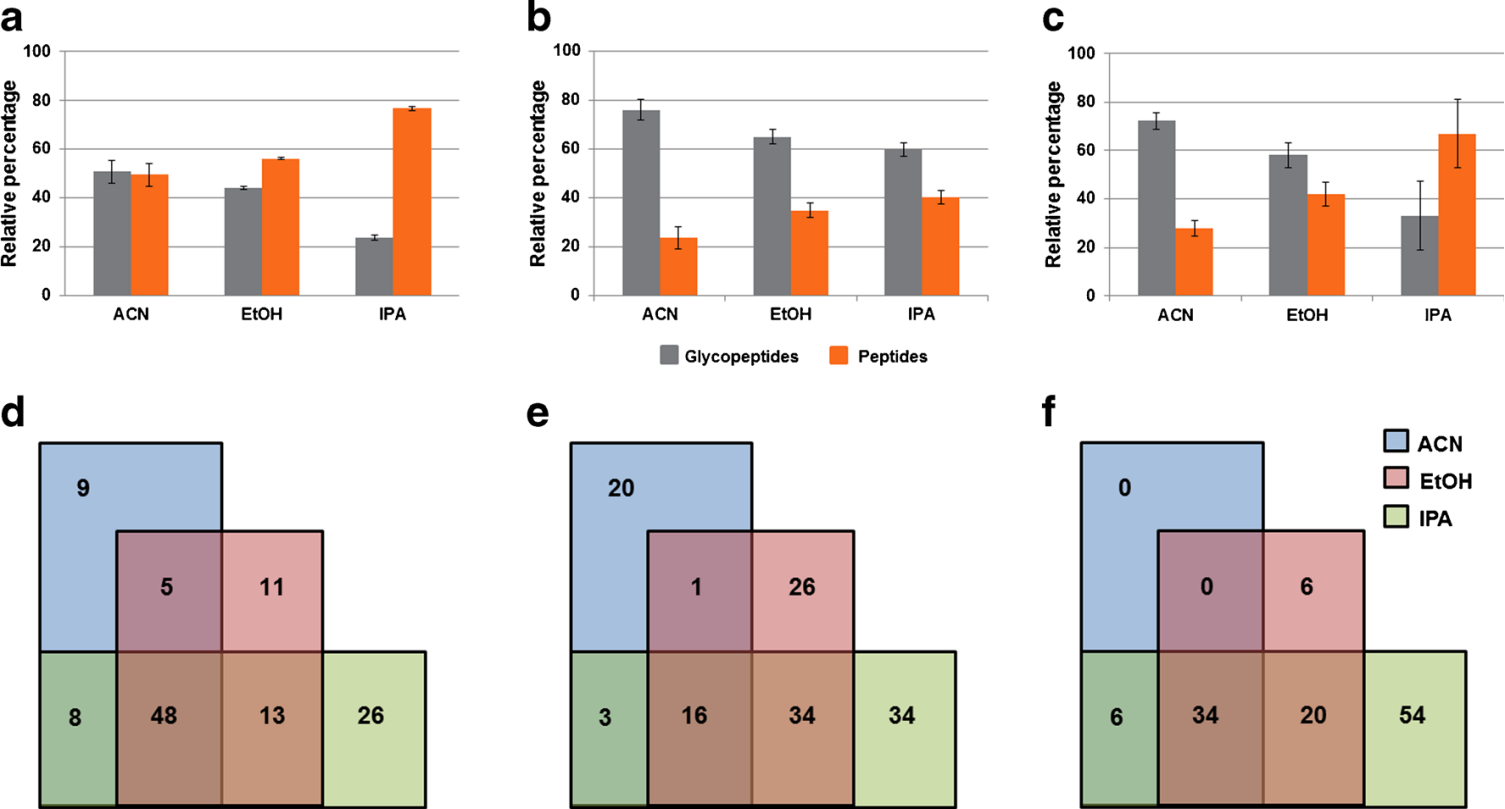
Loading solvent effect on ZIC-HILIC glycopeptide enrichment from (**a**) the four depleted high abundance proteins (**b**) depleted human serum (**c**) un-depleted serum. Depending upon the complexity of the sample, the relative percentage of the peptides and glycopeptides present in the HILIC enriched fraction varied in a solvent dependent manner. **d**–**f** Venn diagram showing the overlap of various peptides present in the HILIC enriched fraction. Different subsets of overlapping and distinct peptides were co-enriched in a solvent dependent manner indicating that sample solvation plays a major role in HILIC enrichment in addition to hydrophilic partitioning

Finally, glycopeptide enrichment from human serum before and after depletion of the four most abundant proteins (albumin, A1PI, transferrin and haptoglobin) was evaluated. As observed for the purified standard glycoproteins, the number of enriched glycopeptides varied in a solvent dependant manner (Fig. 4a–c; ESM Table S7). Glycopeptide enrichment efficiencies were determined taking the presence of identified co-enriched, non-glycosylated peptides as an indicator, while a simple automated glycopeptide classification feature available in the ProteinScape software tool was used to establish the number of enriched glycopeptides. After manual verification of the MSMS spectra for oxonium ions, just hits with a minimum oxonium ion intensity score of ≥60 were considered as glycopeptides.

A significant number of relatively low molecular weight, non-glycosylated peptides (between 1000 and 2200 Da) were frequently co-enriched. The degree of non-specific enrichment of higher molecular weight peptides was found to largely dependent on the used solvent (ESM Table S8). A high number of human serum albumin-derived peptides was co-enriched by all loading solvents, but each solvent co-enriched an individual peptide subset (Fig. 4d–f). The enriched glycopeptide fractions were also treated with PNGase F and analysed by RP-nano LC-ESI-MSMS to simply identify enriched, previously glycosylated peptides. The use of acetonitrile, ethanol and isopropanol, respectively, as loading solvent resulted in the identification of 26, 28 and 33 non-redundant, previously glycosylated peptides carrying the deamidated *N*-glycosylation sequence motif. So despite the fact that isopropanol also co-enriched the highest number of unmodified peptides, it also provided the highest number of glycopeptides. These results clearly emphasise that besides (glyco)peptide hydrophilicity sample solvation plays an important role in ZIC-HILIC glycopeptide enrichment.

## Conclusion

With Drop-HILIC a simple, fast and cost-effective optimised sample pre-treatment and ZIC-HILIC glycopeptide enrichment strategy was developed. This technique was applied to evaluate the effect the loading solvent has on glycopeptide enrichment efficiency. Independent of whether glycopeptides were enriched from single, purified glycoproteins or complex (glyco)peptide mixtures derived from human serum, ZIC-HILIC glycopeptide enrichment efficiency largely relied on the applied mobile phase but also on the peptide backbone composition. ACN provided the least number of co-enriched peptides while IPA and ethanol showed some preferable features when larger, hydrophobic glycopeptides needed to be enriched. To the best of our knowledge, this is the first study to systematically investigate the effect the mobile phase has on ZIC-HILIC glycopeptide enrichment. Implementation of orthogonal mobile phase solvents provides one opportunity to increase glycopeptide enrichment efficiency of ZIC-HILIC.

## Electronic supplementary material

Below is the link to the electronic supplementary material.

ESM 1 (PDF 2.01 mb)

ESM 2 (PDF 2.27 mb)
