# Supplementary material for: It is all about the solvent: on the importance of the mobile phase for ZIC-HILIC glycopeptide enrichment
Source: Anal Bioanal Chem. 2016 Dec 1;409(2):529–38. doi: 10.1007/s00216-016-0051-6 (PMC5203826; doi:10.1007/s00216-016-0051-6)
Supplement: Supplementary file 1 — (PDF 2.01 mb) [file 216_2016_51_MOESM1_ESM.pdf]

Analytical and Bioanalytical Chemistry

Electronic Supplementary Material

It is all about the solvent: on the importance of the mobile phase for ZIC-HILIC glycopeptide enrichment

Kathirvel Alagesan, Sana Khan Khilji, Daniel Kolarich

Table_S6A - 216_2016_51_MOESM2_ESM.pdf

MATERIALS AND METHODS

Table S1 Ion trap settings applied in this study, following the MIRAGE guidelines (www.beilstein-mirage.org)

**MS Settings - General**	
ESI probe	CaptiveSpray™
Capillary voltage	1.3 kV
SPS	*m/z* 900
Compound stability	100%
Trap Drive Level	100%
Spectra averaging	5
Dry gas temperature	150°C
Dry Gas flow	3 L/min
Maximum accumulation time	200 ms
Ion mode	positive
**MS-Scan**	
MS Scan mode	Enhanced scan
ICC target	200000
Mass detection range	*m/z* 350-1800
**MS2**	
MS scan mode	ultrascan
SPS MS(n)	automatic
MS(n) spectra averages	5
MS(n) ICC target	40000
Preferred charge state	≥Doubly,
Active exclusion	Off
Mass detection range	*m/z* 100-2000
Isolation width	3 Da
Exclude singly charged ions	on
No. of precursor ions	3
SmartFrag	Enhanced
SmartFrag Start amplitude	30%
SmartFrag End amplitude	120%
Fragmentation width	5 *m/z*
**Resolution**	
Ultra Scan	32,000 u/s @ 0.5 u FWHM
Enhanced Scan	8,100 u/s @ 0.3 u FWHM

Glycopeptide synthesis

Standard amino acids for SPPS were purchased from Iris Biotech GmbH (Marktredwitz, Germany), and polystyrene and NovaPEG resin were from Novabiochem (Darmstadt, Germany). Fmoc-labelled asparagine (Asn) carrying a disialylated biantennary N-glycan was initially prepared from egg yolks as described previously [1,2]. Sialic acid residues were selectively protected by esterification with benzyl bromide prior their use in SPPS [3]. The coupling of the glycosylated Asn building blocks was performed as described by Unverzagt and co-workers [4]. Synthesised glycopeptides were purified using preparative C18-RP HPLC on an Agilent 1200 series ELSD HPLC system (Agilent, Santa Clara,CA). The lyophilisate was dissolved in water and subjected to C18-RP chromatography (XBridge BEH C18 column, 130 Å, 3.5 µm, 4.6 mm x 150 mm, Waters, Milford, MA) using a linear gradient (1 mL/min) from 1% to 50% acetonitrile containing 0.1% TFA within 35 min, followed by an increase to 100% over 10 min. Fractions of interest from several runs were combined and analysed by MS to confirm their composition.

RESULTS AND DISCUSSION

Incubation Time Does Not Influence Drop-HILIC Enrichment Efficiency

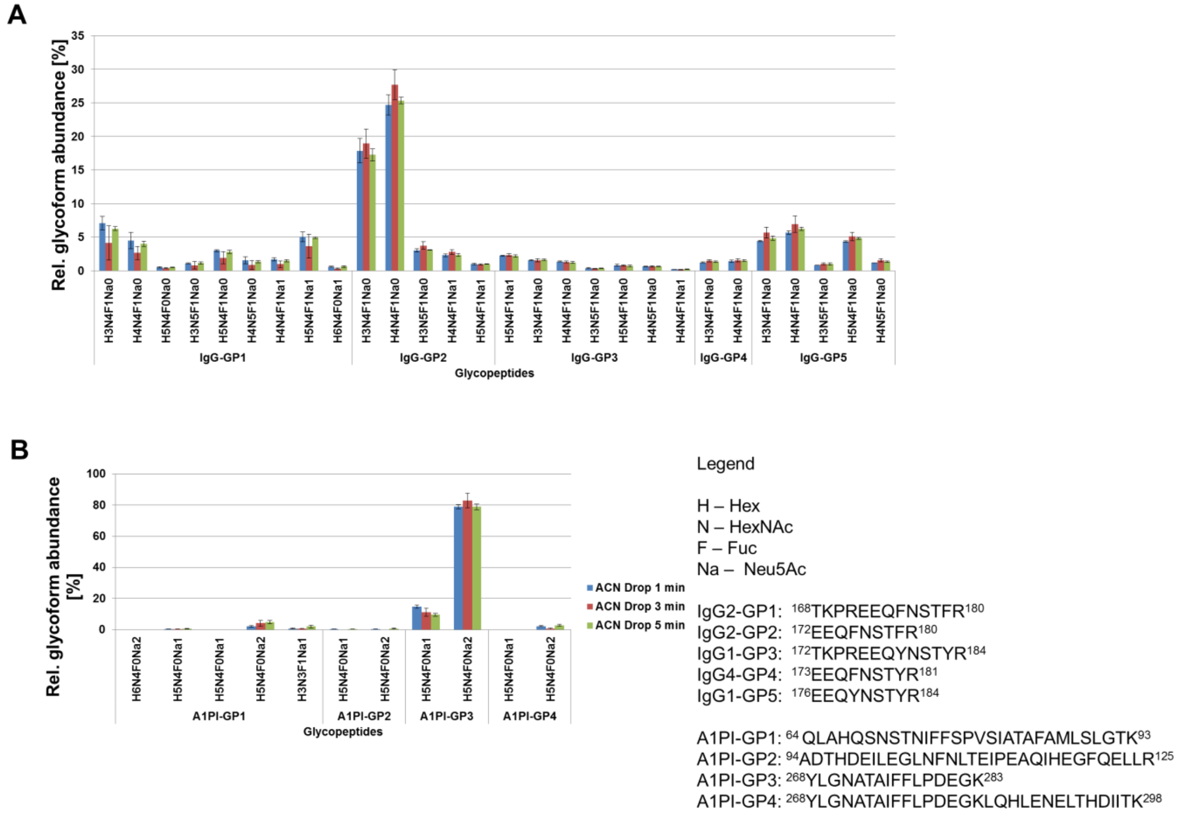

Fig. S1 Effect of incubation time on Drop HILIC efficiency

Table S2 List of IgG glycopeptides identified after HILIC enrichment

Table S2A Raw Data after quantification of identified glycopeptides

SNO	m/z	RT	Z	Calculated	Glycan	Peptide	Hex	HexNAc	H	N	F	Na	Glycan Name	ACN spin E1	ACN spin E2	ACN spin E3	ACN drop 1 min E1	ACN drop 1 min E2	ACN drop 1 min E3	ACN Drop 3 min E1	ACN Drop 3 min E2	ACN Drop 3 min E3	ACN Drop 5 min E1	ACN Drop 5 min E2	ACN Drop 5 min E3	EtOH drop E1	EtOH drop E2	EtOH drop E3	IPA drop E1	IPA drop E2	IPA drop E3
1	1028.89	17.1	3	3083.72	(HexNAc)2 (Deoxyhexose)1 + (Man)3(GlcNAc)2	168-180 TKPREEQFNSTFR	0	2	3	4	1	0	H3N4F1Na0	2.66E+08	5.00E+08	2.12E+08	6.21E+08	4.79E+08	6.61E+08	1.17E+08	2.50E+08	5.78E+08	5.12E+08	4.69E+08	4.75E+08	1.85E+08	1.37E+08	2.12E+08	4.73E+08	5.35E+08	5.95E+08
2	1082.89	17.3	3	3245.84	(Hex)1 (HexNAc)2 (Deoxyhexose)1 + (Man)3(GlcNAc)2	168-180 TKPREEQFNSTFR	1	2	4	4	1	0	H4N4F1Na0	1.97E+08	2.94E+08	1.53E+08	4.86E+08	3.08E+08	3.16E+08	1.02E+08	1.79E+08	3.05E+08	3.12E+08	3.15E+08	2.96E+08	1.53E+08	1.07E+08	1.62E+08	3.19E+08	3.40E+08	3.65E+08
3	1088.21	16.2	3	3261.59	(Hex)2 (HexNAc)2 + (Man)3(GlcNAc)2	168-180 TKPREEQFNSTFR	2	2	5	4	0	0	H5N4F0Na0	3.00E+07	3.68E+07	1.67E+07	4.53E+07	2.79E+07	4.70E+07	1.75E+07	2.95E+07	3.80E+07	4.49E+07	3.18E+07	3.90E+07	2.05E+07	1.71E+07	1.55E+07	3.16E+07	3.03E+07	4.36E+07
4	1096.52	17.3	3	3286.53	(HexNAc)3 (Deoxyhexose)1 + (Man)3(GlcNAc)2	168-180 TKPREEQFNSTFR	0	3	3	5	1	0	H3N5F1Na0	4.69E+07	6.27E+07	3.61E+07	8.82E+07	8.38E+07	9.80E+07	2.10E+07	4.42E+07	1.22E+08	1.11E+08	6.94E+07	9.09E+07	2.76E+07	3.24E+07	3.34E+07	1.12E+08	1.10E+08	1.32E+08
5	1136.84	17.1	3	3407.51	(Hex)2 (HexNAc)2 (Deoxyhexose)1 + (Man)3(GlcNAc)2	168-180 TKPREEQFNSTFR	2	2	5	4	1	0	H5N4F1Na0	1.29E+08	2.08E+08	9.97E+07	2.55E+08	2.29E+08	2.59E+08	6.14E+07	1.31E+08	2.37E+08	2.45E+08	1.81E+08	2.32E+08	9.78E+07	8.92E+07	1.18E+08	2.25E+08	2.11E+08	2.42E+08
6	1150.52	17.4	3	3448.54	(Hex)1 (HexNAc)3 (Deoxyhexose)1 + (Man)3(GlcNAc)2	168-180 TKPREEQFNSTFR	1	3	4	5	1	0	H4N5F1Na0	3.98E+07	1.22E+08	3.55E+07	1.25E+08	8.60E+07	1.75E+08	1.78E+07	4.37E+07	1.34E+08	1.21E+08	7.89E+07	1.10E+08	3.04E+07	3.25E+07	4.93E+07	8.12E+07	1.05E+08	1.31E+08
7	885.15	18.1	4	3536.58	(Hex)1 (HexNAc)2 (Deoxyhexose)1 (NeuAc)1 + (Man)3(GlcNAc)2	168-180 TKPREEQFNSTFR	1	2	4	4	1	1	H4N4F1Na1	7.49E+07	9.57E+07	5.93E+07	1.37E+08	1.20E+08	1.66E+08	3.36E+07	5.99E+07	1.27E+08	1.16E+08	1.01E+08	1.32E+08	4.39E+07	4.49E+07	4.90E+07	1.23E+08	1.29E+08	1.41E+08
8	1234.18	18.2	3	3698.67	(Hex)2 (HexNAc)2 (Deoxyhexose)1 (NeuAc)1 + (Man)3(GlcNAc)2	168-180 TKPREEQFNSTFR	2	2	5	4	1	1	H5N4F1Na1	2.38E+08	3.14E+08	1.69E+08	4.19E+08	3.46E+08	4.88E+08	1.20E+08	2.32E+08	4.64E+08	4.05E+08	3.52E+08	3.88E+08	1.68E+08	1.49E+08	1.63E+08	3.12E+08	3.43E+08	4.32E+08
9	1239.21	16.8	3	3714.6	(Hex)3 (HexNAc)2 (NeuAc)1 + (Man)3(GlcNAc)2	168-180 TKPREEQFNSTFR	3	2	6	4	0	1	H6N4F0Na1	1.69E+07	2.50E+07	1.49E+07	4.98E+07	5.35E+07	4.56E+07	1.25E+07	2.54E+07	2.85E+07	6.36E+07	3.11E+07	4.84E+07	2.81E+07	2.11E+07	2.75E+07	4.15E+07	4.86E+07	5.58E+07
10	1301.74	19.6	2	2601.15	(HexNAc)2 (Deoxyhexose)1 + (Man)3(GlcNAc)2	172-180 EEQFNSTFR	0	2	3	4	1	0	H3N4F1Na0	1.37E+09	1.31E+09	1.38E+09	1.32E+09	1.58E+09	1.53E+09	1.25E+09	1.34E+09	1.40E+09	1.55E+09	1.17E+09	1.32E+09	1.15E+09	1.06E+09	1.13E+09	1.37E+09	1.59E+09	1.68E+09
11	922.14	19.4	3	2764.05	(Hex)1 (HexNAc)2 (Deoxyhexose)1 + (Man)3(GlcNAc)2	172-180 EEQFNSTFR	1	2	4	4	1	0	H4N4F1Na0	1.94E+09	2.17E+09	1.91E+09	2.03E+09	2.11E+09	1.98E+09	1.73E+09	2.04E+09	2.09E+09	2.10E+09	1.84E+09	1.97E+09	1.58E+09	1.57E+09	1.54E+09	1.63E+09	2.07E+09	2.08E+09
12	1403.08	19.6	2	2804.15	(HexNAc)3 (Deoxyhexose)1 + (Man)3(GlcNAc)2	172-180 EEQFNSTFR	0	3	3	5	1	0	H3N5F1Na0	2.44E+08	2.52E+08	2.30E+08	2.40E+08	2.64E+08	2.44E+08	2.51E+08	2.71E+08	2.61E+08	2.62E+08	2.17E+08	2.36E+08	2.15E+08	1.97E+08	2.06E+08	2.15E+08	2.28E+08	2.47E+08
13	1019.09	21.5	3	3054.26	(Hex)1 (HexNAc)2 (Deoxyhexose)1 (NeuAc)1 + (Man)3(GlcNAc)2	172-180 EEQFNSTFR	1	2	4	4	1	1	H4N4F1Na1	1.97E+08	2.08E+08	1.72E+08	1.95E+08	1.95E+08	1.77E+08	1.80E+08	2.06E+08	2.00E+08	1.87E+08	1.82E+08	1.76E+08	1.58E+08	1.43E+08	1.52E+08	1.69E+08	1.85E+08	1.93E+08
14	1073.13	21.5	3	3216.36	(Hex)2 (HexNAc)2 (Deoxyhexose)1 (NeuAc)1 + (Man)3(GlcNAc)2	172-180 EEQFNSTFR	2	2	5	4	1	1	H5N4F1Na1	7.81E+07	7.75E+07	5.78E+07	8.19E+07	7.27E+07	9.21E+07	6.00E+07	6.54E+07	6.67E+07	8.31E+07	7.10E+07	7.72E+07	5.72E+07	5.75E+07	8.53E+07	1.31E+08	1.44E+08	1.48E+08
15	1244.84	16	3	1244.08	$(Hex)_2 (HexNAc)_2 (Deoxyhexose)_1 (NeuAc)_1 + (Man)_3(GlcNAc)_2$	172-184 TKPREEQYNSTYR	2	2	5	4	1	1	H5N4F1Na1	1.62E+08	1.70E+08	1.39E+08	1.84E+08	1.86E+08	1.85E+08	1.24E+08	1.77E+08	2.00E+08	1.81E+08	1.42E+08	1.86E+08	1.34E+08	1.51E+08	1.55E+08	1.60E+08	1.66E+08	1.85E+08
16	1039.52	15.4	3	3115.54	(HexNAc)2 (Deoxyhexose)1 + (Man)3(GlcNAc)2	172-184 TKPREEQYNSTYR	0	2	3	4	1	0	H3N4F1Na0	1.18E+08	1.43E+08	1.09E+08	1.29E+08	1.20E+08	1.38E+08	7.51E+07	1.15E+08	1.44E+08	1.28E+08	1.14E+08	1.35E+08	9.23E+07	8.52E+07	9.84E+07	1.20E+08	1.20E+08	1.61E+08
17	1093.54	15.3	3	3278.76	(Hex)1 (HexNAc)2 (Deoxyhexose)1 + (Man)3(GlcNAc)2	172-184 TKPREEQYNSTYR	1	2	4	4	1	0	H4N4F1Na0	1.10E+08	1.10E+08	8.36E+07	1.07E+08	1.12E+08	1.19E+08	6.62E+07	9.88E+07	1.09E+08	1.15E+08	7.57E+07	9.84E+07	9.22E+07	9.29E+07	8.76E+07	1.05E+08	1.57E+08	1.95E+08
18	1107.19	15.6	3	3318.54	(HexNAc)3 (Deoxyhexose)1 + (Man)3(GlcNAc)2	172-184 TKPREEQYNSTYR	0	3	3	5	1	0	H3N5F1Na0	1.88E+07	3.13E+07	1.80E+07	3.92E+07	2.61E+07	2.97E+07	1.48E+07	1.88E+07	2.76E+07	2.87E+07	2.81E+07	2.94E+07	1.81E+07	1.92E+07	2.11E+07	3.73E+07	3.73E+07	3.89E+07
19	1147.51	15.2	3	3439.49	(Hex)2 (HexNAc)2 (Deoxyhexose)1 + (Man)3(GlcNAc)2	172-184 TKPREEQYNSTYR	2	2	5	4	1	0	H5N4F1Na0	6.75E+07	6.78E+07	5.57E+07	7.44E+07	5.50E+07	7.24E+07	3.96E+07	6.40E+07	6.40E+07	5.97E+07	4.47E+07	6.24E+07	7.33E+07	6.28E+07	5.40E+07	7.42E+07	9.15E+07	7.68E+07
20	1161.19	15.4	3	3480.56	(Hex)1 (HexNAc)3 (Deoxyhexose)1 + (Man)3(GlcNAc)2	172-184 TKPREEQYNSTYR	1	3	4	5	1	0	H4N5F1Na0	4.62E+07	5.68E+07	4.06E+07	5.82E+07	5.04E+07	4.92E+07	3.28E+07	4.95E+07	5.62E+07	5.21E+07	4.93E+07	4.90E+07	3.44E+07	3.04E+07	3.51E+07	4.86E+07	4.50E+07	7.29E+07
21	1190.52	16	3	3568.54	$(Hex)_1 (HexNAc)_2 (Deoxyhexose)_1 (NeuAc)_1 + (Man)_3(GlcNAc)_2$	172-184 TKPREEQYNSTYR	1	2	4	4	1	1	H4N4F1Na1	1.53E+07	1.41E+07	1.20E+07	1.69E+07	1.41E+07	1.65E+07	7.04E+06	1.24E+07	1.48E+07	1.89E+07	1.19E+07	1.57E+07	9.54E+06	1.09E+07	1.05E+07	1.48E+07	1.92E+07	2.01E+07
22	1309.56	18	2	2617.1	(HexNAc)2 (Deoxyhexose)1 + (Man)3(GlcNAc)2	173-181 EEQFNSTYR	0	2	3	4	1	0	H3N4F1Na0	9.72E+07	9.39E+07	8.34E+07	1.05E+08	9.03E+07	1.06E+08	9.70E+07	1.03E+08	1.09E+08	1.05E+08	1.03E+08	9.93E+07	8.01E+07	5.94E+07	6.45E+07	6.74E+07	8.63E+07	9.62E+07
23	1390.57	17.7	2	2779.14	(Hex)1 (HexNAc)2 (Deoxyhexose)1 + (Man)3(GlcNAc)2	173-181 EEQFNSTYR	1	2	4	4	1	0	H4N4F1Na0	1.01E+08	1.05E+08	8.54E+07	1.29E+08	1.00E+08	1.22E+08	1.10E+08	1.01E+08	1.18E+08	1.30E+08	1.16E+08	1.04E+08	9.81E+07	7.72E+07	8.38E+07	8.58E+07	1.19E+08	1.21E+08
24	1317.65	16.4	2	2634.06	(HexNAc)2 (Deoxyhexose)1 + (Man)3(GlcNAc)2	176-184 EEQYNSTYR	0	2	3	4	1	0	H3N4F1Na0	3.61E+08	3.81E+08	3.37E+08	3.57E+08	3.61E+08	3.76E+08	3.84E+08	4.05E+08	4.03E+08	3.80E+08	3.68E+08	3.75E+08	3.23E+08	2.82E+08	3.26E+08	3.25E+08	3.48E+08	3.62E+08
25	932.73	16.3	3	2795.15	(Hex)1 (HexNAc)2 (Deoxyhexose)1 + (Man)3(GlcNAc)2	176-184 EEQYNSTYR	1	2	4	4	1	0	H4N4F1Na0	5.18E+08	5.11E+08	4.60E+08	4.75E+08	4.72E+08	4.57E+08	4.80E+08	4.98E+08	4.70E+08	5.44E+08	4.26E+08	4.89E+08	4.76E+08	4.46E+08	4.43E+08	4.91E+08	5.36E+08	5.31E+08
26	946.41	16.7	3	2836.2	(HexNAc)3 (Deoxyhexose)1 + (Man)3(GlcNAc)2	176-184 EEQYNSTYR	0	3	3	5	1	0	H3N5F1Na0	7.25E+07	7.04E+07	6.12E+07	6.61E+07	6.76E+07	6.74E+07	6.65E+07	7.44E+07	7.37E+07	8.55E+07	7.95E+07	6.36E+07	6.34E+07	6.33E+07	5.55E+07	7.34E+07	6.40E+07	6.27E+07
27	1479.59	16.5	2	2957.16	(Hex)2 (HexNAc)2 (Deoxyhexose)1 + (Man)3(GlcNAc)2	176-184 EEQYNSTYR	2	2	5	4	1	0	H5N4F1Na0	3.60E+08	3.75E+08	3.20E+08	3.55E+08	3.48E+08	3.80E+08	3.38E+08	3.62E+08	3.73E+08	3.99E+08	3.36E+08	3.85E+08	3.49E+08	2.85E+08	3.24E+08	3.35E+08	3.76E+08	3.51E+08
28	1000.4	16.6	3	2998.19	(Hex)1 (HexNAc)3 (Deoxyhexose)1 + (Man)3(GlcNAc)2	176-184 EEQYNSTYR	1	3	4	5	1	0	H4N5F1Na0	9.77E+07	1.07E+08	8.98E+07	9.61E+07	9.51E+07	9.76E+07	1.05E+08	1.13E+08	1.07E+08	1.12E+08	1.05E+08	1.00E+08	1.03E+08	7.72E+07	9.50E+07	9.09E+07	9.11E+07	9.21E+07
														7.01E+09	7.91E+09	6.44E+09	8.28E+09	8.05E+09	8.50E+09	5.91E+09	7.11E+09	8.32E+09	8.45E+09	7.11E+09	7.78E+09	5.86E+09	5.40E+09	5.80E+09	7.26E+09	8.32E+09	8.85E+09

Table S2B Normalized Data after quantification of the identified glycopeptides

SNO	m/z	RT	Z	Calculated mass	Glycan	Peptide	Hex	HexNAc	H	N	F	Na	Glycan Name	ACN spin E1	ACN spin E2	ACN spin E3	ACN drop 1 min E1	ACN drop 1 min E2	ACN drop 1 min E3	ACN Drop 3 min E1	ACN Drop 3 min E2	ACN Drop 3 min E3	ACN Drop 5 min E1	ACN Drop 5 min E2	ACN Drop 5 min E3	EtOH drop E1	EtOH drop E2	EtOH drop E3	IPA drop E1	IPA drop E2	IPA drop E3
1	1028.89	17.1	3	3083.72	(HexNAc)2 (Deoxyhexose)1 + (Man)3(GlcNAc)2	168-180 TKPREEQFNSTFR	0	2	3	4	1	0	H3N4F1Na0	3.79	6.32	3.29	7.50	5.94	7.78	1.98	3.51	6.94	6.05	6.60	6.10	3.15	2.54	3.66	6.52	6.43	6.72
2	1082.89	17.3	3	3245.84	(Hex)1 (HexNAc)2 (Deoxyhexose)1 + (Man)3(GlcNAc)2	168-180 TKPREEQFNSTFR	1	2	4	4	1	0	H4N4F1Na0	2.82	3.71	2.38	5.87	3.83	3.72	1.73	2.52	3.66	3.69	4.42	3.80	2.61	1.98	2.80	4.39	4.09	4.12
3	1088.21	16.2	3	3261.59	(Hex)2 (HexNAc)2 + (Man)3(GlcNAc)2	168-180 TKPREEQFNSTFR	2	2	5	4	0	0	H5N4F0Na0	0.43	0.47	0.26	0.55	0.35	0.55	0.30	0.41	0.46	0.53	0.45	0.50	0.35	0.32	0.27	0.44	0.36	0.49
4	1096.52	17.3	3	3286.53	(HexNAc)3 (Deoxyhexose)1 + (Man)3(GlcNAc)2	168-180 TKPREEQFNSTFR	0	3	3	5	1	0	H3N5F1Na0	0.67	0.79	0.56	1.07	1.04	1.15	0.36	0.62	1.46	1.32	0.98	1.17	0.47	0.60	0.58	1.55	1.32	1.49
5	1136.84	17.1	3	3407.51	(Hex)2 (HexNAc)2 (Deoxyhexose)1 + (Man)3(GlcNAc)2	168-180 TKPREEQFNSTFR	2	2	5	4	1	0	H5N4F1Na0	1.84	2.62	1.55	3.08	2.84	3.05	1.04	1.84	2.85	2.90	2.54	2.98	1.67	1.65	2.03	3.10	2.54	2.74
6	1150.52	17.4	3	3448.54	(Hex)1 (HexNAc)3 (Deoxyhexose)1 + (Man)3(GlcNAc)2	168-180 TKPREEQFNSTFR	1	3	4	5	1	0	H4N5F1Na0	0.57	1.54	0.55	1.51	1.07	2.06	0.30	0.62	1.62	1.44	1.11	1.41	0.52	0.60	0.85	1.12	1.27	1.48
7	885.15	18.1	4	3536.58	(Hex)1 (HexNAc)2 (Deoxyhexose)1 (NeuAc)1 + (Man)3(GlcNAc)2	168-180 TKPREEQFNSTFR	1	2	4	4	1	1	H4N4F1Na1	1.07	1.21	0.92	1.65	1.49	1.96	0.57	0.84	1.53	1.37	1.42	1.69	0.75	0.83	0.85	1.69	1.55	1.59
8	1234.18	18.2	3	3698.67	(Hex)2 (HexNAc)2 (Deoxyhexose)1 (NeuAc)1 + (Man)3(GlcNAc)2	168-180 TKPREEQFNSTFR	2	2	5	4	1	1	H5N4F1Na1	3.39	3.97	2.62	5.06	4.29	5.74	2.03	3.27	5.58	4.79	4.95	4.98	2.87	2.75	2.80	4.29	4.13	4.88
9	1239.21	16.8	3	3714.6	(Hex)3 (HexNAc)2 (NeuAc)1 + (Man)3(GlcNAc)2	168-180 TKPREEQFNSTFR	3	2	6	4	0	1	H6N4F0Na1	0.24	0.32	0.23	0.60	0.66	0.54	0.21	0.36	0.34	0.75	0.44	0.62	0.48	0.39	0.47	0.57	0.58	0.63
10	1301.74	19.6	2	2601.15	(HexNAc)2 (Deoxyhexose)1 + (Man)3(GlcNAc)2	172-180 EEQFNSTFR	0	2	3	4	1	0	H3N4F1Na0	19.52	16.59	21.43	15.94	19.60	18.02	21.08	18.90	16.80	18.29	16.50	16.97	19.58	19.62	19.44	18.83	19.07	18.97
11	922.14	19.4	3	2764.05	(Hex)1 (HexNAc)2 (Deoxyhexose)1 + (Man)3(GlcNAc)2	172-180 EEQFNSTFR	1	2	4	4	1	0	H4N4F1Na0	27.65	27.40	29.63	24.47	26.25	23.31	29.25	28.66	25.15	24.90	25.87	25.27	26.98	29.08	26.62	22.48	24.85	23.52
12	1403.08	19.6	2	2804.15	(HexNAc)3 (Deoxyhexose)1 + (Man)3(GlcNAc)2	172-180 EEQFNSTFR	0	3	3	5	1	0	H3N5F1Na0	3.48	3.18	3.57	2.90	3.28	2.87	4.24	3.81	3.14	3.10	3.05	3.04	3.66	3.66	3.56	2.96	2.74	2.79
13	1019.09	21.5	3	3054.26	(Hex)1 (HexNAc)2 (Deoxyhexose)1 (NeuAc)1 + (Man)3(GlcNAc)2	172-180 EEQFNSTFR	1	2	4	4	1	1	H4N4F1Na1	2.81	2.63	2.68	2.36	2.42	2.08	3.05	2.90	2.40	2.21	2.56	2.26	2.70	2.64	2.62	2.32	2.23	2.18
14	1073.13	21.5	3	3216.36	(Hex)2 (HexNAc)2 (Deoxyhexose)1 (NeuAc)1 + (Man)3(GlcNAc)2	172-180 EEQFNSTFR	2	2	5	4	1	1	H5N4F1Na1	1.11	0.98	0.90	0.99	0.90	1.08	1.02	0.92	0.80	0.98	1.00	0.99	0.98	1.07	1.47	1.80	1.73	1.67
15	1244.84	16	3	1244.08	$(Hex)_2 (HexNAc)_2 (Deoxyhexose)_1 (NeuAc)_1 + (Man)_3(GlcNAc)_2$	172-184 TKPREEQYNSTYR	2	2	5	4	1	1	H5N4F1Na1	2.31	2.15	2.16	2.23	2.31	2.18	2.10	2.48	2.40	2.14	1.99	2.40	2.29	2.79	2.68	2.20	1.99	2.09
16	1039.52	15.4	3	3115.54	(HexNAc)2 (Deoxyhexose)1 + (Man)3(GlcNAc)2	172-184 TKPREEQYNSTYR	0	2	3	4	1	0	H3N4F1Na0	1.69	1.80	1.69	1.56	1.49	1.63	1.27	1.62	1.74	1.51	1.60	1.74	1.58	1.58	1.70	1.66	1.45	1.81
17	1093.54	15.3	3	3278.76	(Hex)1 (HexNAc)2 (Deoxyhexose)1 + (Man)3(GlcNAc)2	172-184 TKPREEQYNSTYR	1	2	4	4	1	0	H4N4F1Na0	1.57	1.39	1.30	1.29	1.39	1.40	1.12	1.39	1.31	1.36	1.06	1.26	1.57	1.72	1.51	1.45	1.89	2.20
18	1107.19	15.6	3	3318.54	(HexNAc)3 (Deoxyhexose)1 + (Man)3(GlcNAc)2	172-184 TKPREEQYNSTYR	0	3	3	5	1	0	H3N5F1Na0	0.27	0.40	0.28	0.47	0.32	0.35	0.25	0.26	0.33	0.34	0.40	0.38	0.31	0.36	0.36	0.51	0.45	0.44
19	1147.51	15.2	3	3439.49	(Hex)2 (HexNAc)2 (Deoxyhexose)1 + (Man)3(GlcNAc)2	172-184 TKPREEQYNSTYR	2	2	5	4	1	0	H5N4F1Na0	0.96	0.86	0.86	0.90	0.68	0.85	0.67	0.90	0.77	0.71	0.63	0.80	1.25	1.16	0.93	1.02	1.10	0.87
20	1161.19	15.4	3	3480.56	(Hex)1 (HexNAc)3 (Deoxyhexose)1 + (Man)3(GlcNAc)2	172-184 TKPREEQYNSTYR	1	3	4	5	1	0	H4N5F1Na0	0.66	0.72	0.63	0.70	0.63	0.58	0.56	0.70	0.68	0.62	0.69	0.63	0.59	0.56	0.61	0.67	0.54	0.82
21	1190.52	16	3	3568.54	$(Hex)_1 (HexNAc)_2 (Deoxyhexose)_1 (NeuAc)_1 + (Man)_3(GlcNAc)_2$	172-184 TKPREEQYNSTYR	1	2	4	4	1	1	H4N4F1Na1	0.22	0.18	0.19	0.20	0.17	0.19	0.12	0.17	0.18	0.22	0.17	0.20	0.16	0.20	0.18	0.20	0.23	0.23
22	1309.56	18	2	2617.1	(HexNAc)2 (Deoxyhexose)1 + (Man)3(GlcNAc)2	173-181 EEQFNSTYR	0	2	3	4	1	0	H3N4F1Na0	1.39	1.19	1.30	1.27	1.12	1.25	1.64	1.45	1.31	1.24	1.45	1.28	1.37	1.10	1.11	0.93	1.04	1.09
23	1390.57	17.7	2	2779.14	(Hex)1 (HexNAc)2 (Deoxyhexose)1 + (Man)3(GlcNAc)2	173-181 EEQFNSTYR	1	2	4	4	1	0	H4N4F1Na0	1.44	1.33	1.33	1.56	1.25	1.43	1.87	1.42	1.42	1.54	1.63	1.34	1.67	1.43	1.45	1.18	1.43	1.37
24	1317.65	16.4	2	2634.06	(HexNAc)2 (Deoxyhexose)1 + (Man)3(GlcNAc)2	176-184 EEQYNSTYR	0	2	3	4	1	0	H3N4F1Na0	5.15	4.81	5.24	4.31	4.48	4.43	6.50	5.70	4.85	4.49	5.18	4.82	5.52	5.22	5.63	4.48	4.18	4.09
25	932.73	16.3	3	2795.15	(Hex)1 (HexNAc)2 (Deoxyhexose)1 + (Man)3(GlcNAc)2	176-184 EEQYNSTYR	1	2	4	4	1	0	H4N4F1Na0	7.39	6.46	7.15	5.73	5.86	5.38	8.12	7.00	5.64	6.44	5.99	6.29	8.12	8.26	7.63	6.76	6.45	6.00
26	946.41	16.7	3	2836.2	(HexNAc)3 (Deoxyhexose)1 + (Man)3(GlcNAc)2	176-184 EEQYNSTYR	0	3	3	5	1	0	H3N5F1Na0	1.03	0.89	0.95	0.80	0.84	0.79	1.13	1.05	0.89	1.01	1.12	0.82	1.08	1.17	0.96	1.01	0.77	0.71
27	1479.59	16.5	2	2957.16	(Hex)2 (HexNAc)2 (Deoxyhexose)1 + (Man)3(GlcNAc)2	176-184 EEQYNSTYR	2	2	5	4	1	0	H5N4F1Na0	5.14	4.74	4.97	4.28	4.33	4.47	5.72	5.08	4.48	4.72	4.73	4.95	5.96	5.29	5.59	4.61	4.52	3.97
28	1000.4	16.6	3	2998.19	(Hex)1 (HexNAc)3 (Deoxyhexose)1 + (Man)3(GlcNAc)2	176-184 EEQYNSTYR	1	3	4	5	1	0	H4N5F1Na0	1.39	1.36	1.40	1.16	1.18	1.15	1.78	1.59	1.29	1.33	1.48	1.29	1.76	1.43	1.64	1.25	1.09	1.04
														100.00	100.00	100.00	100.00	100.00	100.00	100.00	100.00	100.00	100.00	100.00	100.00	100.00	100.00	100.00	100.00	100.00	100.00

Table S3 List of A1PI glycopeptides identified after HILIC enrichment

Table S3A Raw Data after quantification of identified glycopeptides

SNO	m/z	RT	Z	Calculated mass	Glycan	PEP	Hex	HexNAc	H	N	F	Na	Glycan Name	ACN spin E1	ACN spin E2	ACN spin E3	ACN drop 1 min E1	ACN drop 1 min E2	ACN drop 1 min E3	ACN Drop 3 min E1	ACN Drop 3 min E2	ACN Drop 3 min E3	ACN Drop 5 min E1	ACN Drop 5 min E2	ACN Drop 5 min E3	EtOH drop E1	EtOH drop E2	EtOH drop E3	IPA drop E1	IPA drop E2	IPA drop E3
1	1388.55	40.8	4	5547.36	(Hex)3 (HexNAc)2 (NeuAc)2 + (Man)3(GlcNAc)2	64-93 QLAHQSNSTNIFFSPVSIATAFAMLSLGTK	3	2	6	4	0	2	H6N4F0Na2	2.97E+07	1.47E+07	1.59E+07	0.00E+00	0.00E+00	0.00E+00	0.00E+00	0.00E+00	0.00E+00	0.00E+00	0.00E+00	0.00E+00	0.00E+00	0.00E+00	0.00E+00	1.41E+08	8.32E+07	7.76E+07
2	1352.08	40.9	4	5401.4	(Hex)2 (HexNAc)2 (NeuAc)2 + (Man)3(GlcNAc)2	64-93 QLAHQSNSTNIFFSPVSIATAFAMLSLGTK	2	2	5	4	0	1	H5N4F0Na1	6.53E+08	3.90E+08	3.14E+08	2.11E+07	3.22E+07	1.11E+08	3.57E+07	4.21E+07	2.52E+07	4.08E+08	8.36E+07	8.98E+07	5.74E+07	1.43E+08	1.36E+08	2.94E+09	2.24E+09	1.93E+09
3	1279.38	38.4	4	5110.52	(Hex)2 (HexNAc)2 (NeuAc)1 + (Man)3(GlcNAc)2	64-93 QLAHQSNSTNIFFSPVSIATAFAMLSLGTK	2	2	5	4	0	1	H5N4F0Na1	5.97E+07	6.28E+07	5.91E+07	2.88E+06	8.83E+06	1.40E+07	3.48E+06	6.64E+06	2.45E+06	4.51E+07	9.30E+06	1.18E+07	7.59E+06	1.53E+07	1.68E+07	2.80E+08	1.97E+08	1.97E+08
4	1275.36	39.7	4	5094.56	(Hex)2 (HexNAc)2 (NeuAc)1 + (Man)3(GlcNAc)2	64-93 QLAHQSNSTNIFFSPVSIATAFAMLSLGTK	2	2	5	4	0	2	H5N4F0Na2	1.45E+07	2.01E+07	1.79E+07	0.00E+00	0.00E+00	0.00E+00	0.00E+00	0.00E+00	0.00E+00	0.00E+00	0.00E+00	0.00E+00	3.09E+06	6.61E+06	7.39E+06	3.19E+07	2.89E+07	3.06E+07
5	1475.63	38.1	3	4422.39	(HexNAc)1 (Deoxyhexose)1 + (Man)3(GlcNAc)2	64-93 QLAHQSNSTNIFFSPVSIATAFAMLSLGTK	0	1	3	3	1	1	H3N3F1Na1	2.99E+08	2.24E+08	1.68E+08	4.40E+06	1.24E+07	4.09E+07	4.33E+06	3.65E+06	3.79E+06	2.14E+08	3.04E+07	3.31E+07	3.67E+07	7.75E+07	8.38E+07	1.76E+09	1.06E+09	9.47E+08
6	1402.85	36	4	5604.36	(Hex)2 (HexNAc)2 (NeuAc)1 + (Man)3(GlcNAc)2	94-125 ADTHDEILEGLNFNLTEIPEAQIHEGFQELLR	2	2	5	4	0	1	H5N4F0Na1	2.66E+07	3.18E+07	4.12E+07	3.32E+06	6.90E+06	7.84E+06	0.00E+00	0.00E+00	0.00E+00	3.43E+07	5.16E+06	5.89E+06	6.26E+06	1.32E+07	1.21E+07	1.52E+08	9.19E+07	1.21E+08
7	1180.83	38	5	5896.05	(Hex)2 (HexNAc)2 (NeuAc)2 + (Man)3(GlcNAc)2	94-125 ADTHDEILEGLNFNLTEIPEAQIHEGFQELLR	2	2	5	4	0	2	H5N4F0Na2	9.37E+07	6.59E+07	6.22E+07	1.19E+06	6.55E+06	1.61E+07	0.00E+00	0.00E+00	0.00E+00	7.09E+07	1.28E+07	1.20E+07	1.29E+07	2.23E+07	2.63E+07	4.40E+08	2.83E+08	2.30E+08
8	1224.6	33	3	3668.7	(Hex)2 (HexNAc)2 (NeuAc)1 + (Man)3(GlcNAc)2	268-283 YLGNATAIFFLPDEGK	2	2	5	4	0	1	H5N4F0Na1	2.77E+08	4.02E+08	3.72E+08	1.68E+08	2.44E+08	6.08E+08	1.21E+08	1.45E+08	4.79E+07	5.92E+08	1.63E+08	2.47E+08	1.16E+08	2.22E+08	2.85E+08	7.13E+08	6.85E+08	6.88E+08
9	1321.29	34.7	3	3959.67	(Hex)2 (HexNAc)2 (NeuAc)2 + (Man)3(GlcNAc)2	268-283 YLGNATAIFFLPDEGK	2	2	5	4	0	2	H5N4F0Na2	3.56E+09	2.92E+09	2.36E+09	9.21E+08	1.19E+09	3.44E+09	7.22E+08	1.55E+09	3.30E+08	5.24E+09	1.41E+09	1.83E+09	1.35E+09	2.28E+09	2.77E+09	8.12E+09	8.20E+09	7.02E+09
10	1092.17	34.6	5	5453.8	(Hex)2 (HexNAc)2 (NeuAc)1 + (Man)3(GlcNAc)2	268-298 YLGNATAIFFLPDEGKLQHLENELTHDIITK	2	2	5	4	0	1	H5N4F0Na1	1.64E+07	6.15E+07	4.29E+07	0.00E+00	0.00E+00	0.00E+00	0.00E+00	0.00E+00	0.00E+00	0.00E+00	0.00E+00	0.00E+00	0.00E+00	0.00E+00	0.00E+00	3.63E+07	2.71E+07	3.27E+07
11	1150.36	35.9	5	5744.95	(Hex)2 (HexNAc)2 (NeuAc)2 + (Man)3(GlcNAc)2	268-298 YLGNATAIFFLPDEGKLQHLENELTHDIITK	2	2	5	4	0	2	H5N4F0Na2	2.89E+08	5.49E+08	3.75E+08	1.88E+07	2.69E+07	9.62E+07	6.43E+06	4.73E+06	4.32E+06	1.66E+08	3.58E+07	6.92E+07	4.10E+07	8.86E+07	7.69E+07	6.33E+08	3.33E+08	6.00E+08
12	1438.8	36	4	5744.24	(Hex)2 (HexNAc)2 (NeuAc)2 + (Man)3(GlcNAc)2	268-298 YLGNATAIFFLPDEGKLQHLENELTHDIITK	2	2	5	4	0	2	H5N4F0Na2	5.88E+07	8.11E+07	5.53E+07	5.47E+06	4.18E+06	1.87E+07	0.00E+00	0.00E+00	0.00E+00	3.03E+07	2.74E+06	1.05E+07	7.15E+06	1.84E+07	1.48E+07	1.36E+08	6.96E+07	1.27E+08
														5.38E+09	4.83E+09	3.89E+09	1.15E+09	1.53E+09	4.36E+09	8.93E+08	1.75E+09	4.14E+08	6.81E+09	1.75E+09	2.31E+09	1.64E+09	2.89E+09	3.43E+09	1.54E+10	1.33E+10	1.20E+10

Table S3B Normalized Data after quantification of the identified glycopeptides

	m/z	RT	Z	Calculated mass	Glycan	PEP	Hex	HexNAc	H	N	F	Na	Glycan Name	ACN spin E1	ACN spin E2	ACN spin E3	ACN drop 1 min E1	ACN drop 1 min E2	ACN drop 1 min E3	ACN Drop 3 min E1	ACN Drop 3 min E2	ACN Drop 3 min E3	ACN Drop 5 min E1	ACN Drop 5 min E2	ACN Drop 5 min E3	EtOH drop E1	EtOH drop E2	EtOH drop E3	IPA drop E1	IPA drop E2	IPA drop E3
1	1388.55	40.8	4	5547.36	(Hex)3 (HexNAc)2 (NeuAc)2 + (Man)3(GlcNAc)2	64-93 QLAHQSNSTNIFFSPVSIATAFAMLSLGTK	3	2	6	4	0	2	H6N4F0Na2	0.55	0.30	0.41	0.00	0.00	0.00	0.00	0.00	0.00	0.00	0.00	0.00	0.00	0.00	0.00	0.92	0.63	0.65
2	1352.08	40.9	4	5401.4	(Hex)2 (HexNAc)2 (NeuAc)2 + (Man)3(GlcNAc)2	64-93 QLAHQSNSTNIFFSPVSIATAFAMLSLGTK	2	2	5	4	0	1	H5N4F0Na1	12.14	8.09	8.08	1.84	2.10	2.54	3.99	2.41	6.10	5.99	4.77	3.89	3.50	4.93	3.95	19.09	16.84	16.12
3	1279.38	38.4	4	5110.52	(Hex)2 (HexNAc)2 (NeuAc)1 + (Man)3(GlcNAc)2	64-93 QLAHQSNSTNIFFSPVSIATAFAMLSLGTK	2	2	5	4	0	1	H5N4F0Na1	1.11	1.30	1.52	0.25	0.58	0.32	0.39	0.38	0.59	0.66	0.53	0.51	0.46	0.53	0.49	1.82	1.48	1.64
4	1275.36	39.7	4	5094.56	(Hex)2 (HexNAc)2 (NeuAc)1 + (Man)3(GlcNAc)2	64-93 QLAHQSNSTNIFFSPVSIATAFAMLSLGTK	2	2	5	4	0	2	H5N4F0Na2	0.27	0.42	0.46	0.00	0.00	0.00	0.00	0.00	0.00	0.00	0.00	0.00	0.19	0.23	0.22	0.21	0.22	0.26
5	1475.63	38.1	3	4422.39	(HexNAc)1 (Deoxyhexose)1 + (Man)3(GlcNAc)2	64-93 QLAHQSNSTNIFFSPVSIATAFAMLSLGTK	0	1	3	3	1	1	H3N3F1Na1	5.56	4.63	4.32	0.38	0.81	0.94	0.49	0.21	0.92	3.15	1.73	1.43	2.24	2.68	2.44	11.46	8.00	7.89
6	1402.85	36	4	5604.36	(Hex)2 (HexNAc)2 (NeuAc)1 + (Man)3(GlcNAc)2	94-125 ADTHDEILEGLNFNLTEIPEAQIHEGFQELLR	2	2	5	4	0	1	H5N4F0Na1	0.49	0.66	1.06	0.29	0.45	0.18	0.00	0.00	0.00	0.50	0.29	0.25	0.38	0.46	0.35	0.99	0.69	1.01
7	1180.83	38	5	5896.05	(Hex)2 (HexNAc)2 (NeuAc)2 + (Man)3(GlcNAc)2	94-125 ADTHDEILEGLNFNLTEIPEAQIHEGFQELLR	2	2	5	4	0	2	H5N4F0Na2	1.74	1.37	1.60	0.10	0.43	0.37	0.00	0.00	0.00	1.04	0.73	0.52	0.79	0.77	0.77	2.86	2.12	1.91
8	1224.6	33	3	3668.7	(Hex)2 (HexNAc)2 (NeuAc)1 + (Man)3(GlcNAc)2	268-283 YLGNATAIFFLPDEGK	2	2	5	4	0	1	H5N4F0Na1	5.14	8.34	9.58	14.63	15.94	13.96	13.56	8.29	11.57	8.70	9.30	10.69	7.09	7.68	8.30	4.64	5.15	5.73
9	1321.29	34.7	3	3959.67	(Hex)2 (HexNAc)2 (NeuAc)2 + (Man)3(GlcNAc)2	268-283 YLGNATAIFFLPDEGK	2	2	5	4	0	2	H5N4F0Na2	66.21	60.55	60.80	80.39	77.67	79.05	80.85	88.44	79.78	77.06	80.45	79.26	82.42	79.01	80.80	52.79	61.65	58.46
10	1092.17	34.6	5	5453.8	(Hex)2 (HexNAc)2 (NeuAc)1 + (Man)3(GlcNAc)2	268-298 YLGNATAIFFLPDEGKLQHLENELTHDIITK	2	2	5	4	0	1	H5N4F0Na1	0.30	1.27	1.10	0.00	0.00	0.00	0.00	0.00	0.00	0.00	0.00	0.00	0.00	0.00	0.00	0.24	0.20	0.27
11	1150.36	35.9	5	5744.95	(Hex)2 (HexNAc)2 (NeuAc)2 + (Man)3(GlcNAc)2	268-298 YLGNATAIFFLPDEGKLQHLENELTHDIITK	2	2	5	4	0	2	H5N4F0Na2	5.38	11.38	9.64	1.64	1.75	2.21	0.72	0.27	1.04	2.45	2.04	3.00	2.50	3.07	2.24	4.11	2.50	5.00
12	1438.8	36	4	5744.24	(Hex)2 (HexNAc)2 (NeuAc)2 + (Man)3(GlcNAc)2	268-298 YLGNATAIFFLPDEGKLQHLENELTHDIITK	2	2	5	4	0	2	H5N4F0Na2	1.09	1.68	1.42	0.48	0.27	0.43	0.00	0.00	0.00	0.45	0.16	0.45	0.44	0.64	0.43	0.88	0.52	1.05

Glycopeptide synthesis

Target glycopeptides were synthesised using the 9-fluorenylmethyl chloroformate (Fmoc) based solid-phase peptide synthesis. Major steps involved in the glycopeptides synthesis are outlined in the figure 6.1, which includes (i) Isolation of glycoamino acid building block (ii) F-moc protection of the Nα amino group of the glycosylated asparagine (iii) Selective Benzyl Esterification of Sialic acid and (iv) Solid phase glycopeptide synthesis (Fig. S2A).

Fmoc protection of glycosylated amino acids

The Nα amino group of the glycosylated asparagine (50 mg) was protected using 9-fluorenylmethyl-succinimidyl-carbonate (FmocOsu) and trimethyl amine in a mixture of dioxane/water (1:1). The reaction was completed within two hours at pH 9. In order to prevent any possible cleavage of the Fmoc group in later stages, the reaction mixture was neutralized using acetic acid and the solvents were removed by rotovap evaporation and subsequent lyophilisation. The Fmoc protected N-glycan-Asn building blocks were purified by solid phase extraction (SPE) using 5 g C18 Sep-Pack Cartridges. The highly hydrophilic nature of the unprotected glycosylated molecule prevents its binding to the C18 materials. In contrary, the hydrophobic nature of the Fmoc group can also act as an affinity tag during this separation, since it imparts a unique hydrophobic group to the highly hydrophilic glycosylated amino acid. The compounds were eluted using 5 and 10% Acetonitrile and lyophilized yielding the Fmoc protected biantennary disialyated aspagine building block (80% yield). The presence of the desired compound was further verified by ESI-MS/MS analysis (Fig. S2-B).

Esterification of sialic acid

The sialic acid residues present on the purified glycosylated amino acid building block from egg yolk was selectively protected using benzyl esterification as described by Kajihara *et al [2].* The amino acid was dissolved in water, titrated with a solution of cesium carbonate and then lyophilized. The dry cesium salt obtained was converted to the corresponding allyl ester by reaction with allyl bromide in DMF at room temperature under argon atmosphere. After the completion of the reaction (24 h), the glycosyl amino acid ester was precipitated using cold dietheyl ether from the reaction mixture (61 % yield), purified and analyzed via ESI-MS/MS analysis (Fig. S2C). Target synthetic

glycopeptides were there further purified and analyzed by LC-ESI-MS/MS (Fig. S3A-S3C).

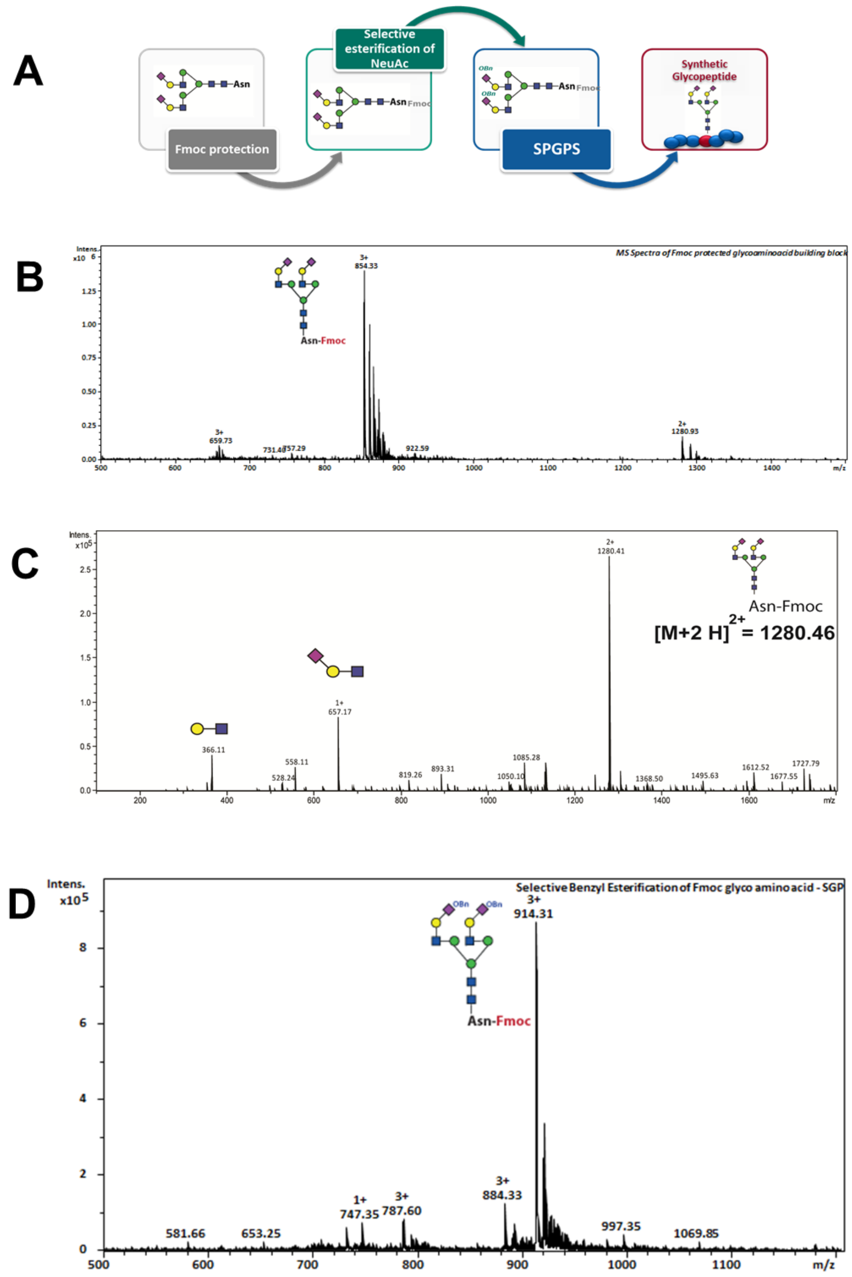

Fig. S2 (A) Schematic representation of major steps involved in solid phase glycopeptide synthesis. (B) MS spectra of Fmoc protected Asn carrying a disialylated, biantennary N-glycan. The signals at m/z 854.02 $[M+3H]^{3+}$ and 1280.46 $[M+2H]^{2+}$correspond to the Fmoc protected glycosylated Asn. Other peaks observed correspond to sodium and potassium adducts of the target compound. (C) CID MSMS spectra of m/z 1280.46 $[M+2H]^{2+}$corresponding to the Fmoc protected glycosylated Asn (D) MS spectra of selective benzyl esterified Fmoc protected Asn carrying a disialylated, biantennary N-glycan (m/z 914.06 $[M+3H]^{3+}$)

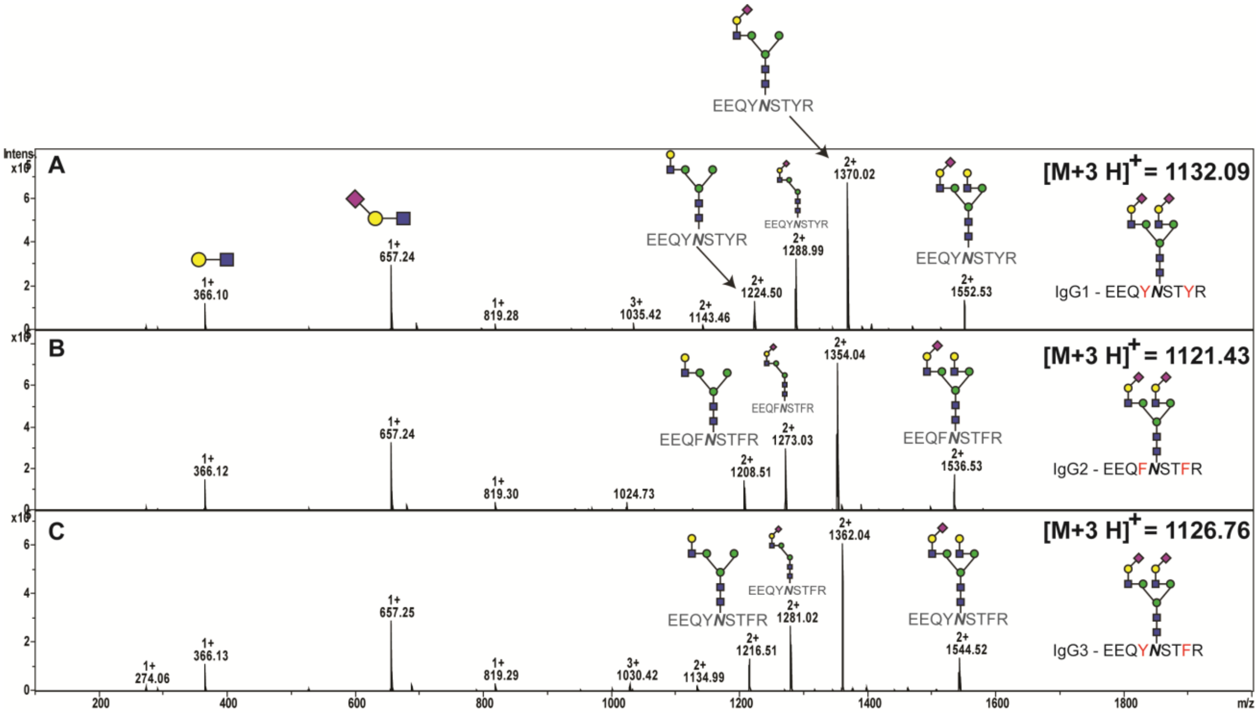

Fig. S3 MS/MS spectrum of a synthetic glycopeptide corresponding to tryptic peptide sequence derived from human Immunoglobulin G (A) IgG-1 (B) IgG-2 (C) IgG-3 subclass. In each case the triply charged precursor ion was isolated and fragmented by collision-induced dissociation

Table S4 GRAVY scores of non-glycosylated peptides identified by RP-nLC

Protein	Sequence NO	Sequence	Gravy Score
A1AT	64-93	QLAHQSNSTNIFFSPVSIATAFAMLSLGTK	0.43
A1AT	94-125	ADTHDEILEGLNFNLTEIPEAQIHEGFQELLR	-0.47
A1AT	268-283	YLGNATAIFFLPDEGK	0.16
A1AT	268-298	YLGNATAIFFLPDEGKLQHLENELTHDIITK	-0.20
IgG2	168-180	TKPREEQFNSTFR	-1.93
IgG2	172-180	EEQFNSTFR	-1.60
IgG1	172-184	TKPREEQYNSTYR	-2.56
IgG1	176-184	EEQYNSTYR	-2.51
IgG4	173-181	EEQFNSTYR	-2.06

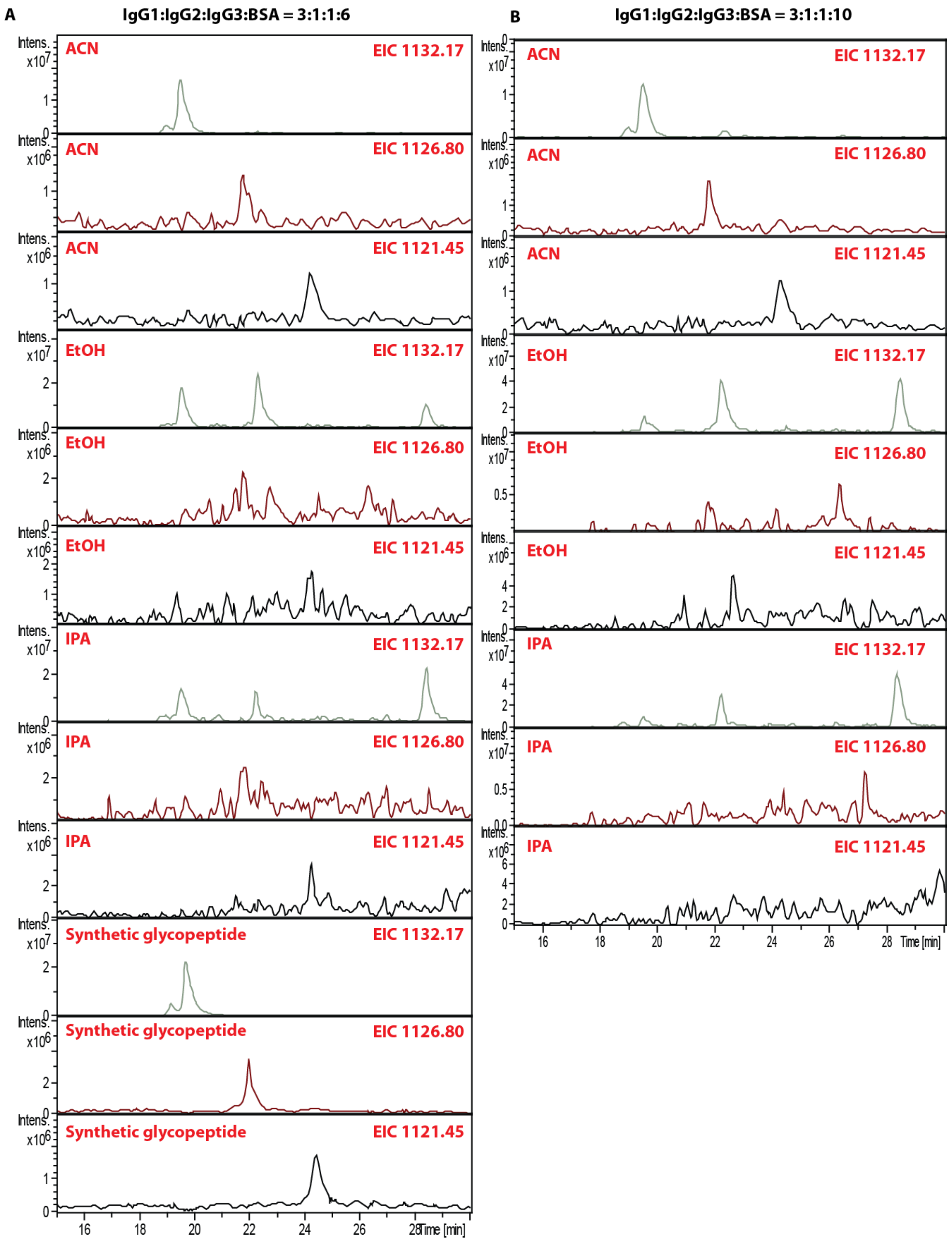

Fig. S4 (A-B) Glycopeptide enrichment efficiency evaluation using the synthetic glycopeptide. When Excess molar ratio of BSA was applied, the enrichment efficiency was significantly compromised in the case of EtOH and IPA as evident from the EIC of the synthetic glycopeptides.

Table S5 List of BSA peptides co-enriched after HILIC enrichment as reported by MASCOT/ProteinScape search for IgG and A1PI

Project Info

**Name:**	Kathir	**Date:**	December 21, 2011
**Sample Info & Protocols**		**Date:**	August 20, 2016
**Name:** abc efficiency check			

Search Result Info

Search Result	Location	Search Engine	Database	Ident. Compounds
Amazon Mammalia_SPJuly2013_2016-08-21 00:28:58	/Kathir/abc efficiency check/KA BSA IgG SGP 3ug ACN_RA2_01_8625.d	Mascot, 2.3.02	SwissProt_July2013, uniprotsprotJuly2013.fasta	31/250 (FDR:9.09%)
Amazon Mammalia_SPJuly2013_2016-08-21 00:28:58	/Kathir/abc efficiency check/KA BSA IgG SGP 3ug ACN_RA2_01_8628.d	Mascot, 2.3.02	SwissProt_July2013, uniprotsprotJuly2013.fasta	16/250 (FDR:0.00%)
Amazon Mammalia_SPJuly2013_2016-08-21 00:28:58	/Kathir/abc efficiency check/KA BSA IgG SGP 3ug ACN T2_RA2_01_8621.d	Mascot, 2.3.02	SwissProt_July2013, uniprotsprotJuly2013.fasta	34/250 (FDR:5.26%)

Protein 1: Serum albumin OS=Bos taurus GN=ALB PE=1 SV=4

**Accession:**	ALBU_BOVIN	**Score:**	2277.02
**Database:**	SwissProt_July2013	**MW [kDa]:**	69.20
**Seq. Coverage [%]:**	58.60 %	**pI:**	5.78
		**No. of Peptides:**	38

Modification(s): Carbamidomethyl, Oxidation, Deamidated

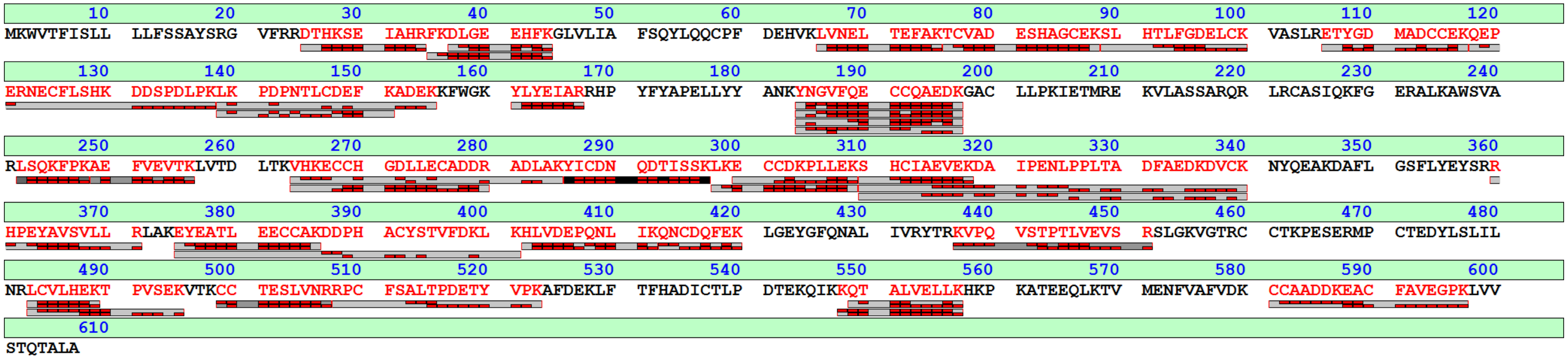

Cmpd.	No. of Cmpds.	m/z meas.	Δ m/z [ppm]	z	Rt [min]	Score	P	Range	Sequence	Modification
3	3	597.3400	59.07	2	14.5	63.3	1	25-34	R.DTHKSEIAHR.F	
42	3	625.4220	172.36	2	17.8	71.0	1	35-44	R.FKDLGEEHFK.G	
41	3	487.8410	222.39	2	17.5	49.3	0	37-44	K.DLGEEHFK.G	
151	2	582.4180	170.06	2	25.0	79.3	0	66-75	K.LVNELTEFAK.T	
7	7	732.4000	139.13	2	14.8	98.2	0	76-88	K.TCVADESHAGCEK.S	Carbamidomethyl: 2, 11
140	1	473.9820	167.24	3	24.0	48.5	0	89-100	K.SLHTLFGDELCK.V	Carbamidomethyl: 11
23	4	747.8430	107.36	2	16.6	81.2	0	106-117	R.ETYGDMADCCEK.Q	Carbamidomethyl: 9, 10; Oxidation: 6
62	1	636.1120	101.69	4	19.5	21.8	2	118-138	K.QEPERNECFLSHKDDSPDLPK.L	Carbamidomethyl: 8
128	1	789.0130	159.18	2	22.9	38.4	0	139-151	K.LKPDPNTLCDEFK.A	Carbamidomethyl: 9

115	2	674.0740	117.84	3	22.3	23.8	1	139-155	K.LKPDPNTLCDEFKADEK.K	Carbamidomethyl: 9
108	3	464.3410	195.24	2	22.1	48.0	0	161-167	K.YLYEIAR.R	
104	3	874.9410	106.11	2	21.7	52.6	0	184-197	K.YNGVFQECCQAEDK.G	Carbamidomethyl: 8, 9; Deamidated: 6
100	3	874.9400	104.97	2	21.7	89.5	0	184-197	K.YNGVFQECCQAEDK.G	Carbamidomethyl: 8, 9; Deamidated: 2
78	3	874.9630	131.26	2	20.5	32.1	0	184-197	K.YNGVFQECCQAEDK.G	Carbamidomethyl: 8, 9; Deamidated: 10
75	5	874.4390	94.74	2	20.5	83.5	0	184-197	K.YNGVFQECCQAEDK.G	Carbamidomethyl: 8, 9
20	4	424.3490	220.51	2	16.0	41.5	1	242-248	R.LSQKFPK.A	
53	3	461.8320	182.67	2	19.3	46.3	0	249-256	K.AEFVEVTK.L	
34	2	1057.5490	97.36	2	17.0	93.4	1	264-280	K.VHKECCHGDLLECADDR.A	Carbamidomethyl: 5, 6, 13
57	1	871.4810	100.77	3	18.9	55.7	2	264-285	K.VHKECCHGDLLECADDRADLAK.Y	Carbamidomethyl: 5, 6, 13
49	6	722.4250	138.92	2	18.2	90.8	0	286-297	K.YICDNQDTISSK.L	Carbamidomethyl: 3
30	3	767.0040	143.18	2	16.9	65.4	1	298-309	K.LKECCDKPLLEK.S	Carbamidomethyl: 4, 5
28	2	646.3910	133.56	2	16.8	57.1	0	300-309	K.ECCDKPLLEK.S	Carbamidomethyl: 2, 3
19	3	536.8760	219.42	2	15.8	47.9	0	310-318	K.SHCIAEVEK.D	Carbamidomethyl: 3
186	4	1171.2790	42.83	3	27.3	102.6	2	310-340	K.SHCIAEVEKDAIPENLPPLTADFAEDKDVCK.N	Carbamidomethyl: 3, 30
190	3	1171.4960	-51.93	3	27.4	53.0	2	310-340	K.SHCIAEVEKDAIPENLPPLTADFAEDKDVCK.N	Carbamidomethyl: 3, 30; Deamidated: 15
96	2	480.7060	202.30	3	21.6	70.1	1	360-371	R.RHPEYAVSVLLR.L	
70	2	751.9260	153.60	2	19.8	77.9	0	375-386	K.EYEATLEECCAK.D	Carbamidomethyl: 9, 10
156	1	1094.2250	375.61	3	24.7	54.5	2	375-401	K.EYEATLEECCAKDDPHACYSTVFDKLK.H	Carbamidomethyl: 9, 10, 18
98	1	653.4550	142.80	2	21.4	59.9	0	402-412	K.HLVDEPQNLIK.Q	
17	2	534.8180	175.08	2	15.8	52.7	0	413-420	K.QNCDQFEK.L	Carbamidomethyl: 3
120	2	547.3940	139.89	3	22.7	73.2	1	437-451	R.KVPQVSTPTLVEVSR.S	
26	2	449.8320	194.80	2	16.8	43.2	0	483-489	R.LCVLHEK.T	Carbamidomethyl: 2
40	2	514.0300	165.72	3	17.8	51.0	1	483-495	R.LCVLHEKTPVSEK.V	Carbamidomethyl: 2
36	3	569.8390	151.62	2	17.5	67.8	0	499-507	K.CCTESLVNR.R	Carbamidomethyl: 1, 2
143	1	628.0330	617.83	3	24.1	59.5	0	508-523	R.RPCFSALTPDETYVPK.A	Carbamidomethyl: 3
113	2	571.9780	204.93	2	22.3	77.4	1	548-557	K.KQTALVELLK.H	

173	1	507.9300	229.76	2	26.2	52.2	0	549-557	K.QTALVELLK.H	
67	1	643.3430	112.01	3	19.9	84.0	1	581-597	K.CCAADDKEACFAVEGPK.L	Carbamidomethyl: 1, 2, 10

Table S6A List of peptides identified in the glycopeptide enriched fraction as reported by MASCOT/ProteinScape search for IgG and A1PI

MASCOT results provided as separate File.

Table S6B Number of identifed peptides in the glycopeptide enriched fraction as reported by MASCOT/ProteinScape search for IgG and A1PI

	**IgG**		
	EtOH	ISO	ACN
**NO of Peptides**	35	40	41
	25	45	36
	31	47	40
**Mean**	30.33	44	39
**SD**	5.03	3.61	2.65
	**A1PI**		
	EtOH	ISO	ACN
**NO of Peptides**	63	72	57
	58	69	49
	54	68	58
**Mean**	58.33	69.67	54.67
**SD**	4.51	2.08	4.93

Table S6C P value score from statistical t-test performed using sigmaPlot

	***p*-value**	
**Solvent**	**IgG**	**A1AT**
**Acetonitrile and Ethanol**	0.058 (No)	0.008 (Yes)
**Acetonitrile and isopropanol**	0.125 (No)	0.396 (NO)
**Isopropanol and Ethanol**	0.019 (Yes)	0.017 (Yes)

Table S6D Description of search parameters used in ProteinScape for protein identification

Peptide Identification

**Search Method:**	Amazon Human_SPJuly2013	**Version:**	1.2
**Submitted Spectra:**	ALL		
**Precursor from Classification:**	OFF		

Mascot

**Database:**	SwissProt_July2013	**Enzyme:**	Trypsin
**Fixed Modifications:**	Carbamidomethyl (C)	**Var. Modifications:**	Deamidated (NQ) Oxidation (M)
**Missed Cleavages:**	2		
**Instrument Type CID:**	ESI-TRAP	**Taxonomy:**	. . . . Homo sapiens (human)
**Peptide Tolerance:**	0.3 Da		
**Peptide Charge:**	2+, 3+ and 4+	**Instrument Type ETD:**	ETD-TRAP
**Mass:**	Monoisotopic	**MS/MS Tolerance:**	0.5 Da
**Peptide Decoy:**	ON	**#^{13}C:**	1
**Significance Threshold:**	0.05	**Percolator:**	OFF

Protein List Compilation by Protein Extractor

**<u>Protein Filter</u>**	
**Min. No. of Significant Peptide IDs:**	1
**Max. MW [KDA]:**	
**<u>Mascot</u>**	
**Ions Score Cut-off:**	15
**Ions Score Threshold for Significant Peptide IDs:**	60
**Identity Threshold (only Mascot):**	ON
**Peptide Rank Cut-off:**	10
**Min. Peptide Length:**	5

Assessment

Accept proteins if: Mascot > 40.0

Accept peptides if: Mascot > 20.0

For each peptide, accept: top hit compound only

In case of MS/MS spectra matching peptides from more than one identified protein, accept: peptide assigned to highest scoring protein

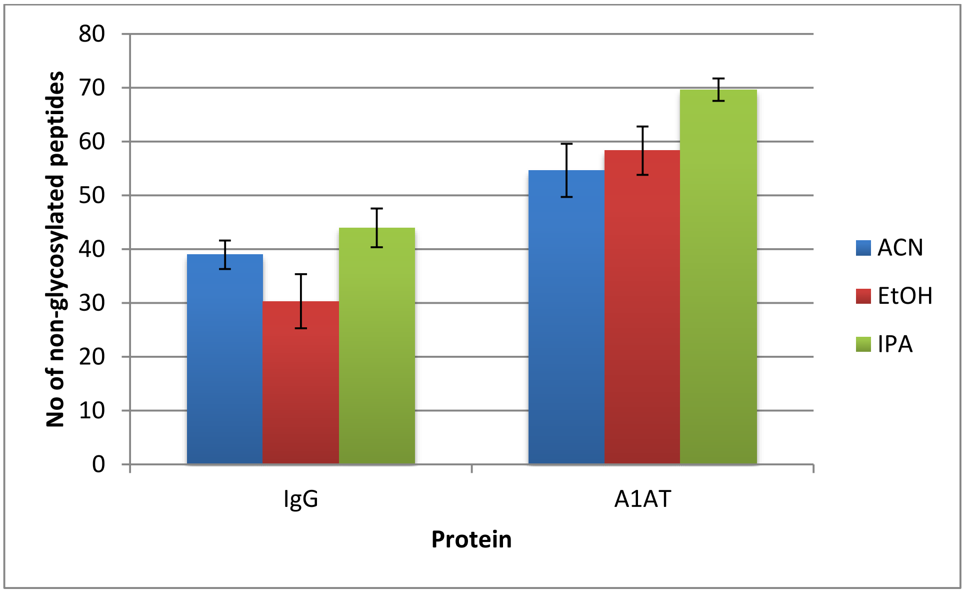

Fig. S5 Number of identified peptides co-enriched along with the glycopeptides using various solvents. As evident from the results, the number of non-glycosylated peptides co-enriched largely depends upon the mobile phase used

Table S7 Number of peptides peptides and glycopeptides identified in the human serum

**High Abundance protein**									
ACN	Trial 1	Trial 2	Trial 3		Trial 1	Trial 2	Trial 3	Mean	SD
No of GP	47.00	54.00	59.00		45.19	54.00	52.68	50.62	4.75
No of pep	57.00	46.00	53.00		54.81	46.00	47.32	49.38	4.75
	104.00	100.00	112.00						
EtOH									
No of GP	46.00	47.00	49.00		43.81	43.52	44.55	43.96	0.53
No of pep	59.00	61.00	61.00		56.19	56.48	55.45	56.04	0.53
	105.00	108.00	110.00						
ISO									
No of GP	23.00	23.00	22.00		22.33	24.21	23.66	23.40	0.97
No of pep	80.00	72.00	71.00		77.67	75.79	76.34	76.60	0.97
	103.00	95.00	93.00						
	**Mean**								
Relative percentage	ACN	EtOH	IPA	SD	SD	SD			
Glycopeptides	50.62	43.96	23.40	4.75	0.53	0.97			
Peptides	49.38	56.04	76.60	4.75	0.53	0.97			
**serum depleted**									
ACN	Trial 1	Trial 2	Trial 3		Trial 1	Trial 2	Trial 3	Mean	SD
No of GP	88.00	105.00	80.00		71.54	80.15	76.92	76.21	4.35
No of pep	35.00	26.00	24.00		28.46	19.85	23.08	23.79	4.35
	123.00	131.00	104.00						
EtOH									
No of GP	104.00	99.00	98.00		64.20	68.28	62.82	65.10	2.84
No of pep	58.00	46.00	58.00		35.80	31.72	37.18	34.90	2.84
	162.00	145.00	156.00						
ISO									
No of Glycopeptides	81.00	96.00	87.00		56.64	61.15	61.70	59.83	2.77
No of peptides	62.00	61.00	54.00		43.36	38.85	38.30	40.17	2.77
	143.00	157.00	141.00						
	**Mean**								
Relative percentage	ACN	EtOH	IPA	SD	SD	SD			
Glycopeptides	76.21	65.10	59.83	4.35	2.84	2.77			
Peptides	23.79	34.90	40.17	4.35	2.84	2.77			
**serum non-depleted**									
ACN	Trial 1	Trial 2	Trial 3		Trial 1	Trial 2	Trial 3	Mean	SD
No of Glycopeptides	80.00	74.00	74.00		74.77	68.52	73.27	72.18	3.26
No of peptides	27.00	34.00	27.00		25.23	31.48	26.73	27.82	3.26
	107.00	108.00	101.00		100.00	100.00	100.00	100.00	0.00
ETOH									
No of Glycopeptides	72.00	46.00	75.00		61.02	52.27	60.98	58.09	5.04
No of peptides	46.00	42.00	48.00		38.98	47.73	39.02	41.91	5.04
	118.00	88.00	123.00						
IPA									
No of Glycopeptides	66.00	25.00	35.00		49.25	23.15	26.92	33.11	14.11
No of peptides	68.00	83.00	95.00		50.75	76.85	73.08	66.89	14.11
	134.00	108.00	130.00						
	**Mean**								
Relative percentage	ACN	EtOH	IPA	SD	SD	SD			
Glycopeptides	72.18	58.09	33.11	3.26	5.04	14.11			
Peptides	27.82	41.91	66.89	3.26	5.04	14.11			

References

1. Seko A, Koketsu M, Nishizono M, Enoki Y, Ibrahim HR, Juneja LR, Kim M, Yamamoto T (1997) Occurrence of a sialylglycopeptide and free sialylglycans in hen's egg yolk. Bba-Gen Subjects 1335 (1-2):23-32. doi:Doi 10.1016/S0304-4165(96)00118-3

2. Kajihara Y, Suzuki Y, Yamamoto N, Sasaki K, Sakakibara T, Juneja LR (2004) Prompt chemoenzymatic synthesis of diverse complex-type oligosaccharides and its application to the solid-phase synthesis of a glycopeptide with asn-linked sialyl-undeca- and asialo-nonasaccharides. Chem-Eur J 10 (4):971-985. doi:DOI 10.1002/chem.200305115

3. Yamamoto N, Ohmori Y, Sakakibara T, Sasaki K, Juneja LR, Kajihara Y (2003) Solid-phase synthesis of sialylglycopeptides through selective esterification of the sialic acid residues of an Asn-linked complex-type sialyloligosaccharide. Angew Chem Int Edit 42 (22):2537-2540. doi:DOI 10.1002/anie.200250572

4. Piontek C, Silva DV, Heinlein C, Pohner C, Mezzato S, Ring P, Martin A, Schmid FX, Unverzagt C (2009) Semisynthesis of a Homogeneous Glycoprotein Enzyme: Ribonuclease C: Part 2. Angew Chem Int Edit 48 (11):1941-1945. doi:DOI 10.1002/anie.200804735
