# Supplementary material for: It is all about the solvent: on the importance of the mobile phase for ZIC-HILIC glycopeptide enrichment
Source: Anal Bioanal Chem. 2016 Dec 1;409(2):529–38. doi: 10.1007/s00216-016-0051-6 (PMC5203826; doi:10.1007/s00216-016-0051-6)
Supplement: Supplementary file 2 — (PDF 2.27 mb) [file 216_2016_51_MOESM2_ESM.pdf]

Table S6A List of peptides identified in the glycopeptide enriched fraction as reported by MASCOT/ProteinScape search for IgG and A1PI

Accumulated Protein Report

Project Info

Name: Kathir **Date:** December 21, 2011

Sample Info & Protocols

Date: August 8, 2016

Name: ABC 2016 IgG And A1AT

Search Type Combined MS/MS - ProteinExtractor

Search Result Amazon Human_SPJuly2013_2016-08-08 18:23:29

Search Location /Kathir/ABC 2016 IgG And A1AT/KA A1AT ACN Drop 1min E1_RA4_01_8263.d/

Protein 1: Serum albumin OS=Homo sapiens GN=ALB PE=1 SV=2

Accession: ALBU_HUMAN **Score:** 2050.44

Database: SwissProt_July2013 **MW [kDa]:** 69.30

Seq. Coverage [%]: 57.60 % **pI:** 5.88

No. of Peptides: 31

Modification(s): Carbamidomethyl, Oxidation

Cmpd.	No. of Cmpds.	m/z meas.	Δ m/z [ppm]	z	Rt [min]	Score	P	Range	Sequence	Modification
42	1	575.3120	35.44	2	14.4	64.2	1	25-34	R.DAHKSEVAHR.F	
100	1	613.8740	110.41	2	19.6	75.5	1	35-44	R.FKDLGEENFK.A	
131	1	575.3580	81.44	2	22.8	75.7	0	66-75	K.LVNEVTEFAK.T	
58	1	749.8410	64.20	2	16.0	112.3	0	76-88	K.TCVADESAENCDK.S	Carbamidomethyl: 2, 11
154	1	1137.9450	65.24	3	26.3	47.5	2	76-105	K.TCVADESAENCDKSLHTLFGDKL CTVATLR.E	Carbamidomethyl: 2, 11, 24
74	2	725.8240	77.43	2	17.3	66.1	0	106-117	R.ETYGEMADCCAK.Q	Carbamidomethyl: 9, 10; Oxidation: 6
66	1	572.3130	74.48	3	16.8	23.1	1	118-130	K.QEPERNECFLQHK.D	Carbamidomethyl: 8
97	1	879.4680	61.57	3	19.3	28.9	2	118-138	K.QEPERNECFLQHKDDNPNLPR.L	Carbamidomethyl: 8
81	1	470.7590	66.32	2	17.9	49.3	0	131-138	K.DDNPNLPR.L	
149	1	926.8780	93.69	3	25.9	34.5	1	139-161	R.LVRPEVDVMCTAFHDNEETFLKK .Y	Carbamidomethyl: 10
104	1	528.3700	136.59	2	19.8	37.3	1	161-168	K.KYLYEIAR.R	
124	1	464.3160	141.39	2	22.2	45.0	0	162-168	K.YLYEIAR.R	
144	2	709.0550	76.61	3	24.5	62.4	1	187-205	K.AAFTECCQAADKAACLLPK.L	Carbamidomethyl: 6, 7, 15

57	1	506.9650	68.79	3	15.8	49.4	2	206-219	K.LDELRDEGKASSAK.Q	
79	1	440.7520	63.13	2	17.7	55.9	0	250-257	K.AEFAEVSK.L	
87	1	1043.9770	52.30	2	18.4	98.6	0	265-281	K.VHTECCHGDLLECADDR.A	Carbamidomethyl: 5, 6, 13
111	2	862.4470	80.67	3	20.6	70.8	1	265-286	K.VHTECCHGDLLECADDRADLAK.Y	Carbamidomethyl: 5, 6, 13
82	1	722.3770	72.47	2	18.0	85.9	0	287-298	K.YICENQDSISSK.L	Carbamidomethyl: 3
67	1	773.9810	102.06	2	16.9	64.5	1	299-310	K.LKECCEKPLLEK.S	Carbamidomethyl: 4, 5
172	2	997.5080	56.83	3	30.3	121.4	0	311-337	K.SHCIAEVENDEMPADLPSLAADFVESK.D	Carbamidomethyl: 3; Oxidation: 12
175	1	992.1660	46.69	3	31.9	92.7	0	311-337	K.SHCIAEVENDEMPADLPSLAADFVESK.D	Carbamidomethyl: 3
143	1	490.0230	143.82	3	24.5	70.3	1	361-372	R.RHPDYSVVLLLR.L	
169	1	1023.1440	90.59	2	29.6	74.3	0	397-413	K.VFDEFKPLVEEPQNLIK.Q	
156	1	829.4610	97.71	2	26.9	93.9	0	414-426	K.QNCELFEQLGEYK.F	Carbamidomethyl: 3
128	1	480.8560	147.88	2	22.5	67.2	0	427-434	K.FQNALLVR.Y	
129	1	820.5440	87.13	2	22.7	78.1	1	438-452	K.KVPQVSTPTLVEVSR.N	
48	1	501.8280	90.57	2	14.9	39.4	1	491-499	K.TPVSDRVTK.C	
148	1	956.0290	62.27	2	25.2	72.6	0	509-524	R.RPCFSALEVDETYVPK.E	Carbamidomethyl: 3
108	1	564.9210	120.42	2	20.2	66.5	1	549-558	K.KQTALVELVK.H	
140	1	500.8670	122.80	2	23.6	68.3	0	550-558	K.QTALVELVK.H	
55	1	814.4130	56.42	2	15.7	59.0	2	585-598	K.ADDKETCFAEEGKK.L	Carbamidomethyl: 7

Protein 2: Alpha-1-antitrypsin OS=Homo sapiens GN=SERPINA1 PE=1 SV=3

Accession: A1AT_HUMAN

Database: SwissProt_July2013

Seq. Coverage [%]: 47.80 %

Score: 1648.77

MW [kDa]: 46.70

pI: 5.27

No. of Peptides: 26

Modification(s): Carbamidomethyl, Oxidation, Deamidated

Cmpd.	No. of Cmpds.	m/z meas.	Δ m/z [ppm]	z	Rt [min]	Score	P	Range	Sequence	Modification
45	1	593.9510	39.47	3	14.7	56.9	0	35-49	K.TDTSHHDQDHPTFNK.I	
167	1	851.4680	69.41	4	29.1	49.0	1	35-63	K.TDTSHHDQDHPTFNKITPNLAEFAFSLYR.Q	
176	1	1288.1800	10.77	2	32.0	124.0	0	126-149	R.TLNQPDSQLQLTTGNGLFLSEGLK.L	Deamidated: 15
135	1	603.4050	103.68	2	23.1	48.9	1	150-159	K.LVDKFLEDVK.K	

114	1	667.4640	110.99	2	20.9	64.8	2	150-160	K.LVDKFLEDVKK.L	
64	1	568.8660	114.37	2	16.7	66.1	1	179-187	K.KQINDYVEK.G	
62	1	804.4890	82.02	2	16.5	89.7	2	179-192	K.KQINDYVEKGTQGK.I	
83	1	504.8000	92.20	2	18.1	43.5	0	180-187	K.QINDYVEK.G	
77	1	740.4070	42.50	2	17.6	33.9	1	180-192	K.QINDYVEKGTQGK.I	
89	1	638.4040	91.09	2	18.6	42.6	1	216-225	K.GKWERPFEVK.D	
139	1	787.9350	62.19	4	23.5	47.9	2	216-241	K.GKWERPFEVKDTEEEDFHVDQVTTVK.V	
110	1	545.8610	134.41	2	20.4	34.5	0	218-225	K.WERPFEVK.D	
133	1	946.5010	73.50	2	23.0	97.0	0	226-241	K.DTEEEDFHVDQVTTVK.V	
141	1	837.4340	57.69	3	23.7	52.9	1	226-246	K.DTEEEDFHVDQVTTVKVPMMK.R	Oxidation: 19, 20
91	1	632.3580	88.13	2	18.7	37.3	0	248-257	R.LGMFNIQHCK.K	Carbamidomethyl: 9; Oxidation: 3
72	1	464.6180	105.62	3	17.0	27.1	1	248-258	R.LGMFNIQHCKK.L	Carbamidomethyl: 9; Oxidation: 3
126	1	602.0520	100.50	3	22.5	52.3	0	284-298	K.LQHLENELTHDIITK.F	
61	1	539.7930	47.36	2	16.3	29.6	1	299-306	K.FLENEDRR.S	
76	1	426.7610	24.54	2	17.5	58.5	0	307-314	R.SASLHLPK.L	
142	1	555.8730	121.09	2	24.1	85.3	0	315-324	K.LSITGTYDLK.S	
147	1	508.3700	116.17	2	25.1	81.8	0	325-334	K.SVLGQLGITK.V	
155	2	918.0180	66.36	2	26.9	133.2	0	335-352	K.VFSNGADLSGVTEEAPLK.L	Deamidated: 4
150	3	917.5370	78.39	2	26.1	97.3	0	335-352	K.VFSNGADLSGVTEEAPLK.L	
151	2	1081.6680	90.05	2	26.2	92.0	1	335-355	K.VFSNGADLSGVTEEAPLKLSK.A	
159	1	721.7740	87.52	3	27.4	54.9	1	335-355	K.VFSNGADLSGVTEEAPLKLSK.A	Deamidated: 4
105	1	444.8000	100.11	2	19.9	47.9	0	360-367	K.AVLTIDEK.G	

Protein 3: Haptoglobin OS=Homo sapiens GN=HP PE=1 SV=1
Accession: HPT_HUMAN
Database: SwissProt_July2013
Seq. Coverage [%]: 2.70 %
Score: 54.73
MW [kDa]: 45.20
pI: 6.13
No. of Peptides: 1

Cmpd.	No. of Cmpds.	m/z meas.	Δ m/z [ppm]	z	Rt [min]	Score	P	Range	Sequence	Modification
44	1	637.8980	118.51	2	14.6	54.7	1	312-322	R.HYEGSTVPEKK.T	

Accumulated Protein Report

Search Type Combined MS/MS - ProteinExtractor
Search Result Amazon Human_SPJuly2013_2016-08-08 18:23:29
Search Location /Kathir/ABC 2016 IgG And A1AT/KA A1AT ACN Drop 1min E1_RA4_01_8296.d/

Protein 1: Serum albumin OS=Homo sapiens GN=ALB PE=1 SV=2
Accession: ALBU_HUMAN
Database: SwissProt_July2013
Seq. Coverage [%]: 47.80 %
Score: 1764.01
MW [kDa]: 69.30
pI: 5.88
No. of Peptides: 28

Modification(s): Carbamidomethyl, Oxidation

Cmpd.	No. of Cmpds.	m/z meas.	Δ m/z [ppm]	z	Rt [min]	Score	P	Range	Sequence	Modification
6	1	575.3810	155.38	2	14.3	59.0	1	25-34	R.DAHKSEVAHR.F	
63	1	613.8800	120.19	2	19.7	75.3	1	35-44	R.FKDLGEENFK.A	
96	1	575.3670	97.08	2	22.9	69.1	0	66-75	K.LVNEVTEFAK.T	
21	1	749.8530	80.21	2	16.1	112.3	0	76-88	K.TCVADESAENCDK.S	Carbamidomethyl: 2, 11
38	1	725.8200	71.92	2	17.4	66.0	0	106-117	R.ETYGEMADCCAK.Q	Carbamidomethyl: 9, 10; Oxidation: 6
30	1	572.3270	98.94	3	16.9	32.8	1	118-130	K.QEPERNECFLQHK.D	Carbamidomethyl: 8
67	1	659.8490	55.75	4	19.9	24.9	2	118-138	K.QEPERNECFLQHKDDNPNLPR.L	Carbamidomethyl: 8
46	1	470.7630	74.82	2	18.0	45.8	0	131-138	K.DDNPNLPR.L	
66	1	528.3730	142.26	2	19.8	34.1	1	161-168	K.KYLYEIAR.R	
88	1	464.3160	141.39	2	22.2	48.0	0	162-168	K.YLYEIAR.R	
108	1	709.0940	131.62	3	24.6	70.0	1	187-205	K.AAFTECCQAADKAACLLPK.L	Carbamidomethyl: 6, 7, 15
20	1	759.9550	83.50	2	15.9	53.6	2	206-219	K.LDELRDEGKASSAK.Q	
45	1	440.7670	97.17	2	17.9	52.4	0	250-257	K.AEFAEVSK.L	
53	1	696.3270	61.71	3	18.5	72.1	0	265-281	K.VHTECCHGDLLECADDR.A	Carbamidomethyl: 5, 6, 13
70	1	862.4390	71.40	3	20.2	61.3	1	265-286	K.VHTECCHGDLLECADDRADLAK.Y	Carbamidomethyl: 5, 6, 13
47	1	722.3820	79.39	2	18.2	82.0	0	287-298	K.YICENQDSISSK.L	Carbamidomethyl: 3
32	1	773.9990	125.31	2	17.0	52.1	1	299-310	K.LKECCEKPLLEK.S	Carbamidomethyl: 4, 5
161	1	997.5360	84.90	3	30.5	115.0	0	311-337	K.SHCIAEVENDEMPADLPSLAADF	Carbamidomethyl: 3; Oxidation:

									VESK.D	12
41	1	492.7830	71.34	2	17.7	45.0	0	376-383	K.TYETTLEK.C	
124	2	829.5100	156.79	2	27.0	106.0	0	414-426	K.QNCELFEQLGEYK.F	Carbamidomethyl: 3
93	1	480.8680	172.84	2	22.6	67.3	0	427-434	K.FQNALLVR.Y	
94	1	547.3760	107.01	3	22.7	94.0	1	438-452	K.KVPQVSTPTLVEVSR.N	
11	1	501.8460	126.44	2	15.1	38.5	1	491-499	K.TPVSDRVTK.C	
112	1	637.7330	132.14	3	25.3	62.8	0	509-524	R.RPCFSALEVDETYVPK.E	Carbamidomethyl: 3
73	1	564.9240	125.73	2	20.3	63.1	1	549-558	K.KQTALVELVK.H	
105	1	500.8930	174.72	2	23.7	48.7	0	550-558	K.QTALVELVK.H	
28	1	750.3750	73.87	2	16.8	60.7	1	585-597	K.ADDKETCFAEEGK.K	Carbamidomethyl: 7
18	1	814.4230	68.70	2	15.8	52.3	2	585-598	K.ADDKETCFAEEGKK.L	Carbamidomethyl: 7

Protein 2: Alpha-1-antitrypsin OS=Homo sapiens GN=SERPINA1 PE=1 SV=3

Accession: A1AT_HUMAN

Score: 1293.34

Database: SwissProt_July2013

MW [kDa]: 46.70

Seq. Coverage [%]: 37.30 %

pI: 5.27

No. of Peptides: 20

Modification(s): Carbamidomethyl, Oxidation, Deamidated

Cmpd.	No. of Cmpds.	m/z meas.	Δ m/z [ppm]	z	Rt [min]	Score	P	Range	Sequence	Modification
15	1	890.4660	87.94	2	15.6	83.6	0	35-49	K.TDTSHHDQDHPTFNK.I	
98	1	603.4220	131.86	2	23.1	48.7	1	150-159	K.LVDKFLEDVK.K	
77	1	667.4760	128.97	2	20.9	71.4	2	150-160	K.LVDKFLEDVKK.L	
27	1	568.8710	123.16	2	16.8	66.0	1	179-187	K.KQINDYVEK.G	
26	1	536.6640	86.15	3	16.6	57.0	2	179-192	K.KQINDYVEKGTQGK.I	
48	1	504.8090	110.03	2	18.2	41.6	0	180-187	K.QINDYVEK.G	
43	1	740.4280	70.86	2	17.7	52.5	1	180-192	K.QINDYVEKGTQGK.I	
55	1	638.4150	108.32	2	18.7	42.2	1	216-225	K.GKWERPFEVK.D	
74	1	545.8500	114.25	2	20.4	34.3	0	218-225	K.WERPFEVK.D	
99	1	946.5090	81.96	2	23.1	101.1	0	226-241	K.DTEEEDFHVDQVTTVK.V	
56	1	632.3790	121.34	2	18.9	43.6	0	248-257	R.LGMFNIQHCK.K	Carbamidomethyl: 9; Oxidation: 3
92	1	602.0500	97.18	3	22.6	48.6	0	284-298	K.LQHLENELTHDIITK.F	
24	1	539.7920	45.51	2	16.4	33.9	1	299-306	K.FLENEDRR.S	

40	1	426.7630	29.23	2	17.6	52.2	0	307-314	R.SASLHLPK.L	
106	1	555.8870	146.28	2	24.2	81.2	0	315-324	K.LSITGTYDLK.S	
110	1	508.3980	171.26	2	25.1	76.9	0	325-334	K.SVLGQLGITK.V	
132	2	918.0660	118.65	2	27.7	129.1	0	335-352	K.VFSNGADLSGVTEEAPLK.L	Deamidated: 4
116	3	917.5700	114.36	2	26.2	118.1	0	335-352	K.VFSNGADLSGVTEEAPLK.L	
133	1	721.8100	137.40	3	27.8	30.0	1	335-355	K.VFSNGADLSGVTEEAPLKLSK.A	Deamidated: 4
68	1	444.8050	111.35	2	20.0	62.9	0	360-367	K.AVLTIDEK.G	

Protein 3: Haptoglobin OS=Homo sapiens GN=HP PE=1 SV=1
Accession: HPT_HUMAN
Database: SwissProt_July2013
Seq. Coverage [%]: 2.70 %
Score: 57.24
MW [kDa]: 45.20
pI: 6.13
No. of Peptides: 1

Cmpd.	No. of Cmpds.	m/z meas.	Δ m/z [ppm]	z	Rt [min]	Score	P	Range	Sequence	Modification
8	1	637.9170	148.30	2	14.6	57.2	1	312-322	R.HYEGSTVPEKK.T	

Search Type Combined MS/MS - ProteinExtractor
Search Result Amazon Human_SPJuly2013_2016-08-08 18:23:29
Search Location /Kathir/ABC 2016 IgG And A1AT/KA A1AT EtOH Drop 1min E1_RD4_01_8281.d/

Protein 1: Serum albumin OS=Homo sapiens GN=ALB PE=1 SV=2
Accession: ALBU_HUMAN
Database: SwissProt_July2013
Seq. Coverage [%]: 48.60 %
Score: 1932.74
MW [kDa]: 69.30
pI: 5.88
No. of Peptides: 28

Modification(s): Carbamidomethyl, Oxidation

Cmpd.	No. of Cmpds.	m/z meas.	Δ m/z [ppm]	z	Rt [min]	Score	P	Range	Sequence	Modification
26	1	575.3480	98.02	2	14.4	59.0	1	25-34	R.DAHKSEVAHR.F	

86	2	613.8570	82.72	2	19.7	75.5	1	35-44	R.FKDLGEENFK.A	
116	1	575.3740	109.25	2	22.8	75.1	0	66-75	K.LVNEVTEFAK.T	
47	1	749.8350	56.20	2	16.0	107.2	0	76-88	K.TCVADESAENCDK.S	Carbamidomethyl: 2, 11
63	1	725.8160	66.41	2	17.4	56.6	0	106-117	R.ETYGEMADCCAK.Q	Carbamidomethyl: 9, 10; Oxidation: 6
84	2	879.4530	44.51	3	19.4	22.6	2	118-138	K.QEPERNECFLQHKDDNPNLPR.L	Carbamidomethyl: 8
71	1	470.7900	132.18	2	18.0	54.5	0	131-138	K.DDNPNLPR.L	
89	1	528.3520	102.51	2	19.8	51.8	1	161-168	K.KYLYEIAR.R	
108	1	464.3110	130.62	2	22.1	45.0	0	162-168	K.YLYEIAR.R	
45	1	759.9480	74.28	2	15.9	60.9	2	206-219	K.LDELRDEGKASSAK.Q	
77	1	1043.9700	45.59	2	18.5	86.9	0	265-281	K.VHTECCHGDLLECADDR.A	Carbamidomethyl: 5, 6, 13
92	1	862.4300	60.96	3	20.2	59.8	1	265-286	K.VHTECCHGDLLECADDRADLAK.Y	Carbamidomethyl: 5, 6, 13
72	1	722.3640	54.47	2	18.2	93.5	0	287-298	K.YICENQDSISSK.L	Carbamidomethyl: 3
59	1	773.9810	102.06	2	17.0	62.0	1	299-310	K.LKECCEKPLLEK.S	Carbamidomethyl: 4, 5
189	4	997.5460	94.93	3	30.5	128.0	0	311-337	K.SHCIAEVENDEMPADLPSLAADFVESK.D	Carbamidomethyl: 3; Oxidation: 12
196	1	992.2020	82.98	3	32.0	109.2	0	311-337	K.SHCIAEVENDEMPADLPSLAADFVESK.D	Carbamidomethyl: 3
42	1	599.3170	58.30	2	15.7	46.7	1	338-347	K.DVCKNYAEAK.D	Carbamidomethyl: 3
131	1	490.0520	203.00	3	24.6	73.0	1	361-372	R.RHPDYSVVLLLR.L	
184	1	682.4560	126.07	3	29.6	63.0	0	397-413	K.VFDEFKPLVEEPQNLIK.Q	
154	1	829.4770	117.00	2	27.0	97.1	0	414-426	K.QNCELFEQLGEYK.F	Carbamidomethyl: 3
115	1	820.5470	90.79	2	22.7	71.3	1	438-452	K.KVPQVSTPTLVEVSR.N	
32	1	501.8200	74.62	2	15.0	33.9	1	491-499	K.TPVSDRVTK.C	
65	1	569.7810	49.82	2	17.7	46.4	0	500-508	K.CCTESLVNR.R	Carbamidomethyl: 1, 2
135	1	637.7220	114.89	3	25.3	85.6	0	509-524	R.RPCFSALEVDETYVPK.E	Carbamidomethyl: 3
93	1	564.9120	104.49	2	20.2	66.6	1	549-558	K.KQTALVELVK.H	
123	1	500.8900	168.73	2	23.6	54.1	0	550-558	K.QTALVELVK.H	
56	1	750.3840	85.87	2	16.8	69.5	1	585-597	K.ADDKETCFAEEGK.K	Carbamidomethyl: 7
44	1	814.4130	56.42	2	15.8	59.7	2	585-598	K.ADDKETCFAEEGKK.L	Carbamidomethyl: 7

Protein 2: Alpha-1-antitrypsin OS=Homo sapiens GN=SERPINA1 PE=1 SV=3

Accession: A1AT_HUMAN

Database: SwissProt_July2013

Seq. Coverage [%]: 42.10 %

Score: 1571.51

MW [kDa]: 46.70

pI: 5.27

No. of Peptides: 24

Accumulated Protein Report

Modification(s): Carbamidomethyl, Oxidation, Deamidated

Cmpd.	No. of Cmpds.	m/z meas.	Δ m/z [ppm]	z	Rt [min]	Score	P	Range	Sequence	Modification
30	4	890.5040	130.62	2	14.8	80.0	0	35-49	K.TDTSHHDQDHPTFNK.I	
98	1	667.4590	103.50	2	20.9	68.2	2	150-160	K.LVDKFLEDVKK.L	
101	1	772.1100	77.91	3	21.1	95.7	2	160-179	K.KLYHSEAFTVNFGDTEEAKK.Q	
132	1	686.7160	91.44	3	24.7	63.1	0	161-178	K.LYHSEAFTVNFGDTEEAK.K	
113	1	729.3970	62.34	3	22.6	65.8	1	161-179	K.LYHSEAFTVNFGDTEEAKK.Q	
54	1	568.8610	105.58	2	16.8	73.1	1	179-187	K.KQINDYVEK.G	
53	1	536.6670	91.74	3	16.6	60.1	2	179-192	K.KQINDYVEKGTQGK.I	
73	1	504.7990	90.21	2	18.2	40.8	0	180-187	K.QINDYVEK.G	
66	1	740.4200	60.05	2	17.7	40.8	1	180-192	K.QINDYVEKGTQGK.I	
79	1	638.3690	36.26	2	18.7	44.1	1	216-225	K.GKWERPFEVK.D	
126	1	1050.5680	58.15	3	23.8	105.1	2	216-241	K.GKWERPFEVKDTEEEDFHVDQVT TVK.V	Deamidated: 21
94	1	545.8570	127.08	2	20.4	34.4	0	218-225	K.WERPFEVK.D	
119	1	946.5080	80.90	2	23.1	81.0	0	226-241	K.DTEEEDFHVDQVTTVK.V	
69	1	473.9460	87.97	3	17.8	22.0	1	247-257	K.RLGMFNIQHCK.K	Carbamidomethyl: 10; Oxidation: 4
61	1	464.6120	92.71	3	17.1	31.0	1	248-258	R.LGMFNIQHCKK.L	Carbamidomethyl: 9; Oxidation: 3
111	1	902.5710	96.84	2	22.6	100.3	0	284-298	K.LQHLENELTHDIITK.F	
50	1	539.8000	60.33	2	16.4	31.5	1	299-306	K.FLENEDRR.S	
64	1	426.7750	57.35	2	17.6	48.8	0	307-314	R.SASLHLPK.L	
129	1	555.9000	169.67	2	24.2	83.2	0	315-324	K.LSITGTYDLK.S	
134	1	508.4000	175.19	2	25.1	81.8	0	325-334	K.SVLGQLGITK.V	
163	1	918.0490	100.13	2	27.6	118.7	0	335-352	K.VFSNGADLSGVTEEAPLK.L	Deamidated: 4
141	2	917.5580	101.28	2	26.2	93.8	0	335-352	K.VFSNGADLSGVTEEAPLK.L	
160	2	1082.1440	75.21	2	27.4	61.1	1	335-355	K.VFSNGADLSGVTEEAPLKLSK.A	Deamidated: 4
90	1	444.8030	106.86	2	20.0	46.9	0	360-367	K.AVLTIDEK.G	

Protein 3: Haptoglobin OS=Homo sapiens GN=HP PE=1 SV=1
Accession: HPT_HUMAN **Score:** 118.07
Database: SwissProt_July2013 **MW [kDa]:** 45.20

Accumulated Protein Report

Seq. Coverage [%]: 4.90 %

pI: 6.13
No. of Peptides: 2

Cmpd.	No. of Cmpds.	m/z meas.	Δ m/z [ppm]	z	Rt [min]	Score	P	Range	Sequence	Modification
107	1	490.7980	95.65	2	21.9	58.1	0	278-286	R.VGYVSGWGR.N	
28	1	637.8900	105.97	2	14.6	59.9	1	312-322	R.HYEGSTVPEKK.T	

Search Type Combined MS/MS - ProteinExtractor
Search Result Amazon Human_SPJuly2013_2016-08-08 18:23:29
Search Location /Kathir/ABC 2016 IgG And A1AT/KA A1AT ACN Drop 1min E2_RA5_01_8252.d/

Protein 1: Serum albumin OS=Homo sapiens GN=ALB PE=1 SV=2
Accession: ALBU_HUMAN
Database: SwissProt_July2013
Seq. Coverage [%]: 54.40 %

Score: 1960.71
MW [kDa]: 69.30
pI: 5.88
No. of Peptides: 28

Modification(s): Carbamidomethyl, Oxidation

Cmpd.	No. of Cmpds.	m/z meas.	Δ m/z [ppm]	z	Rt [min]	Score	P	Range	Sequence	Modification
21	1	575.3230	54.56	2	14.4	62.5	1	25-34	R.DAHKSEVAHR.F	
76	1	613.8800	120.19	2	19.7	75.4	1	35-44	R.FKDLGEENFK.A	
113	1	575.3610	86.66	2	22.8	68.7	0	66-75	K.LVNEVTEFAK.T	
36	1	749.8420	65.53	2	16.0	103.1	0	76-88	K.TCVADESAENCDK.S	Carbamidomethyl: 2, 11
141	1	1137.9580	76.66	3	26.4	75.5	2	76-105	K.TCVADESAENCDKSLHTLFGDKL CTVATLR.E	Carbamidomethyl: 2, 11, 24
72	1	879.4740	68.39	3	19.4	28.6	2	118-138	K.QEPERNECFLQHKDDNPNLPR.L	Carbamidomethyl: 8
56	1	470.7810	113.06	2	18.0	43.3	0	131-138	K.DDNPNLPR.L	
136	1	695.4180	104.70	4	26.0	51.1	1	139-161	R.LVRPEVDVMCTAFHDNEETFLKK .Y	Carbamidomethyl: 10
104	1	464.3140	137.08	2	22.2	45.0	0	162-168	K.YLYEIAR.R	
128	1	709.0600	83.66	3	24.6	66.8	1	187-205	K.AAFTECCQAADKAACLLPK.L	Carbamidomethyl: 6, 7, 15

35	1	759.9560	84.81	2	15.8	67.9	2	206-219	K.LDELRDEGKASSAK.Q	
55	1	440.7670	97.17	2	17.8	59.1	0	250-257	K.AEFAEVSK.L	
64	2	1043.9990	73.37	2	18.5	79.3	0	265-281	K.VHTECCHGDLLECADDR.A	Carbamidomethyl: 5, 6, 13
80	2	862.4440	77.20	3	20.1	61.2	1	265-286	K.VHTECCHGDLLECADDRADLAK.Y	Carbamidomethyl: 5, 6, 13
57	1	722.3860	84.93	2	18.1	88.1	0	287-298	K.YICENQDSISSK.L	Carbamidomethyl: 3
45	1	773.9960	121.44	2	16.9	58.7	1	299-310	K.LKECCEKPLLEK.S	Carbamidomethyl: 4, 5
166	7	997.5200	68.86	3	30.4	133.7	0	311-337	K.SHCIAEVENDEMPADLPSLAADFVESK.D	Carbamidomethyl: 3; Oxidation: 12
176	1	992.1790	59.79	3	32.0	98.1	0	311-337	K.SHCIAEVENDEMPADLPSLAADFVESK.D	Carbamidomethyl: 3
129	1	490.0180	133.61	3	24.6	73.2	1	361-372	R.RHPDYSVVLLLR.L	
163	2	682.4370	98.22	3	29.8	81.0	0	397-413	K.VFDEFKPLVEEPQNLIK.Q	
144	1	829.4640	101.33	2	26.9	101.3	0	414-426	K.QNCELFEQLGEYK.F	Carbamidomethyl: 3
112	1	820.5460	89.57	2	22.7	72.5	1	438-452	K.KVPQVSTPTLVEVSR.N	
26	1	501.8250	84.59	2	14.9	30.9	1	491-499	K.TPVSDRVTK.C	
132	1	956.0360	69.59	2	25.2	73.7	0	509-524	R.RPCFSALEVDETYVPK.E	Carbamidomethyl: 3
83	1	564.9260	129.27	2	20.3	68.2	1	549-558	K.KQTALVELVK.H	
125	1	500.8760	140.77	2	23.8	60.7	0	550-558	K.QTALVELVK.H	
43	1	750.3780	77.87	2	16.8	66.5	1	585-597	K.ADDKETCFAEEGK.K	Carbamidomethyl: 7
32	1	814.4210	66.25	2	15.7	66.5	2	585-598	K.ADDKETCFAEEGKK.L	Carbamidomethyl: 7

Protein 2: Alpha-1-antitrypsin OS=Homo sapiens GN=SERPINA1 PE=1 SV=3

Accession: A1AT_HUMAN
Database: SwissProt_July2013
Seq. Coverage [%]: 51.90 %

Score: 1892.10
MW [kDa]: 46.70
pI: 5.27
No. of Peptides: 28

Modification(s): Carbamidomethyl, Oxidation, Deamidated

Cmpd.	No. of Cmpds.	m/z meas.	Δ m/z [ppm]	z	Rt [min]	Score	P	Range	Sequence	Modification
30	1	890.4710	93.56	2	15.4	62.3	0	35-49	K.TDTSHHDQDHPTFNK.I	
159	1	851.7340	381.83	4	29.2	28.2	1	35-63	K.TDTSHHDQDHPTFNKITPNLAEFAFSLYR.Q	
177	1	859.1420	33.55	3	32.0	97.3	0	126-149	R.TLNQPDSQLQLTTGNGLFLSEGLK.L	Deamidated: 15
171	2	1287.7150	31.74	2	31.1	142.3	0	126-149	R.TLNQPDSQLQLTTGNGLFLSEGL	

									K.L	
118	1	603.4020	98.71	2	23.1	42.4	1	150-159	K.LVDKFLEDVK.K	
94	1	667.4690	118.48	2	21.0	64.5	2	150-160	K.LVDKFLEDVKK.L	
98	1	772.1090	76.61	3	21.1	83.3	2	160-179	K.KLYHSEAFTVNFGDTEEAKK.Q	
108	1	729.4300	107.59	3	22.6	57.1	1	161-179	K.LYHSEAFTVNFGDTEEAKK.Q	
42	1	568.8720	124.92	2	16.7	68.3	1	179-187	K.KQINDYVEK.G	
41	1	804.4930	87.00	2	16.6	87.2	2	179-192	K.KQINDYVEKGTQGK.I	
58	1	504.8080	108.04	2	18.2	43.4	0	180-187	K.QINDYVEK.G	
52	1	740.4300	73.56	2	17.7	30.0	1	180-192	K.QINDYVEKGTQGK.I	
67	1	638.4220	119.29	2	18.7	52.3	1	216-225	K.GKWERPFEVK.D	
123	1	787.9360	63.46	4	23.6	44.3	2	216-241	K.GKWERPFEVKDTEEEDFHVDQVT TVK.V	
86	1	545.8520	117.92	2	20.5	34.5	0	218-225	K.WERPFEVK.D	
115	1	946.5000	72.45	2	23.1	97.8	0	226-241	K.DTEEEDFHVDQVTTVK.V	
124	1	837.4480	74.40	3	23.7	65.2	1	226-246	K.DTEEEDFHVDQVTTVKVPMMK.R	Oxidation: 19, 20
66	1	632.3630	96.04	2	18.7	39.6	0	248-257	R.LGMFNIQHCK.K	Carbamidomethyl: 9; Oxidation: 3
109	1	602.0520	100.50	3	22.6	59.2	0	284-298	K.LQHLENELTHDIITK.F	
39	1	539.7860	34.39	2	16.3	31.9	1	299-306	K.FLENEDRR.S	
51	1	426.7710	47.97	2	17.6	52.4	0	307-314	R.SASLHLPK.L	
126	1	555.8710	117.49	2	24.2	74.0	0	315-324	K.LSITGTYDLK.S	
131	1	508.3680	112.24	2	25.1	81.8	0	325-334	K.SVLGQLGITK.V	
143	2	918.0300	79.43	2	26.9	130.5	0	335-352	K.VFSNGADLSGVTEEAPLK.L	Deamidated: 4
137	3	917.5360	77.30	2	26.2	103.2	0	335-352	K.VFSNGADLSGVTEEAPLK.L	
139	3	1081.6730	94.67	2	26.3	67.8	1	335-355	K.VFSNGADLSGVTEEAPLKLSK.A	
142	2	1082.1470	77.98	2	26.8	88.8	1	335-355	K.VFSNGADLSGVTEEAPLKLSK.A	Deamidated: 4
79	1	444.8030	106.86	2	20.0	62.5	0	360-367	K.AVLTIDEK.G	

Protein 3: Haptoglobin OS=Homo sapiens GN=HP PE=1 SV=1

**Accession:**	HPT_HUMAN	**Score:**	56.67
**Database:**	SwissProt_July2013	**MW [kDa]:**	45.20
**Seq. Coverage [%]:**	2.70 %	**pI:**	6.13
		**No. of Peptides:**	1

Cmpd.	No. of Cmpds.	m/z meas.	Δ m/z [ppm]	z	Rt [min]	Score	P	Range	Sequence	Modification
23	1	637.9070	132.62	2	14.6	56.7	1	312-322	R.HYEGSTVPEKK.T	

Search Type Combined MS/MS - ProteinExtractor
Search Result Amazon Human_SPJuly2013_2016-08-08 18:23:29
Search Location /Kathir/ABC 2016 IgG And A1AT/KA A1AT ACN Drop 3min E2_RB3_01_8253.d/

Protein 1: Serum albumin OS=Homo sapiens GN=ALB PE=1 SV=2
Accession: ALBU_HUMAN
Database: SwissProt_July2013
Seq. Coverage [%]: 36.00 %
Score: 1246.00
MW [kDa]: 69.30
pI: 5.88
No. of Peptides: 20
Modification(s): Carbamidomethyl

Cmpd.	No. of Cmpds.	m/z meas.	Δ m/z [ppm]	z	Rt [min]	Score	P	Range	Sequence	Modification
1	1	575.3380	80.64	2	14.4	58.9	1	25-34	R.DAHKSEVAHR.F	
46	1	613.8850	128.33	2	19.7	75.5	1	35-44	R.FKDLGEENFK.A	
76	1	575.3560	77.96	2	22.8	68.5	0	66-75	K.LVNEVTEFAK.T	
13	1	749.8410	64.20	2	16.0	106.6	0	76-88	K.TCVADESAENCDK.S	Carbamidomethyl: 2, 11
93	1	1137.9510	70.51	3	26.4	56.3	2	76-105	K.TCVADESAENCDKSLHTLFGDKL CTVATLR.E	Carbamidomethyl: 2, 11, 24
42	1	879.4980	95.68	3	19.3	26.4	2	118-138	K.QEPERNECFLQHKDDNPNLPR.L	Carbamidomethyl: 8
45	1	666.3620	71.07	3	19.6	39.8	1	123-138	R.NECFLQHKDDNPNLPR.L	Carbamidomethyl: 3
29	1	470.7500	47.20	2	18.0	54.7	0	131-138	K.DDNPNLPR.L	
68	1	464.3210	152.16	2	22.2	49.8	0	162-168	K.YLYEIAR.R	
88	1	709.0680	94.95	3	24.7	62.6	1	187-205	K.AAFTECCQAADKAACLLPK.L	Carbamidomethyl: 6, 7, 15
11	1	759.9600	90.08	2	15.8	61.3	2	206-219	K.LDELRDEGKASSAK.Q	
36	1	1043.9800	55.17	2	18.6	79.8	0	265-281	K.VHTECCHGDLLECADDR.A	Carbamidomethyl: 5, 6, 13
51	1	862.4540	88.79	3	20.3	47.5	1	265-286	K.VHTECCHGDLLECADDRADLAK. Y	Carbamidomethyl: 5, 6, 13
30	1	722.3800	76.62	2	18.1	83.4	0	287-298	K.YICENQDSISSK.L	Carbamidomethyl: 3
21	1	516.3250	105.68	3	17.1	38.2	1	299-310	K.LKECCEKPLLEK.S	Carbamidomethyl: 4, 5

96	1	829.3890	10.90	2	27.0	80.8	0	414-426	K.QNCELFEQLGEYK.F	Carbamidomethyl: 3
74	1	547.3880	128.93	3	22.6	61.5	1	438-452	K.KVPQVSTPTLVEVSR.N	
53	1	564.9200	118.65	2	20.5	64.0	1	549-558	K.KQTALVELVK.H	
81	1	500.8740	136.78	2	23.7	42.7	0	550-558	K.QTALVELVK.H	
9	1	543.3110	117.58	3	15.6	68.2	2	585-598	K.ADDKETCFAEEGKK.L	Carbamidomethyl: 7

Protein 2: Alpha-1-antitrypsin OS=Homo sapiens GN=SERPINA1 PE=1 SV=3
Accession: A1AT_HUMAN **Score:** 1217.92
Database: SwissProt_July2013 **MW [kDa]:** 46.70
Seq. Coverage [%]: 30.60 % **pI:** 5.27
No. of Peptides: 16
Modification(s): Oxidation, Deamidated

Cmpd.	No. of Cmpds.	m/z meas.	Δ m/z [ppm]	z	Rt [min]	Score	P	Range	Sequence	Modification
6	1	890.4060	20.56	2	15.1	71.5	0	35-49	K.TDTSHHDQDHPTFNK.I	
61	1	772.1110	79.20	3	21.3	84.4	2	160-179	K.KLYHSEAFTVNFGDTEEAKK.Q	
73	1	729.4440	126.78	3	22.5	54.6	1	161-179	K.LYHSEAFTVNFGDTEEAKK.Q	
17	1	568.8700	121.40	2	16.7	73.1	1	179-187	K.KQINDYVEK.G	
16	1	804.4970	91.97	2	16.6	106.0	2	179-192	K.KQINDYVEKGTQGK.I	
31	1	504.8050	102.10	2	18.1	42.5	0	180-187	K.QINDYVEK.G	
26	1	740.4460	95.17	2	17.7	34.8	1	180-192	K.QINDYVEKGTQGK.I	
39	1	638.4140	106.75	2	18.8	53.3	1	216-225	K.GKWERPFEVK.D	
78	1	946.5240	97.80	2	23.1	93.7	0	226-241	K.DTEEEDFHVDQVTTVK.V	
82	1	837.4570	85.15	3	23.7	68.6	1	226-246	K.DTEEEDFHVDQVTTVKVPMMK.R	Oxidation: 19, 20
25	1	426.8020	120.62	2	17.5	59.0	0	307-314	R.SASLHLPK.L	
86	1	555.8730	121.09	2	24.2	89.6	0	315-324	K.LSITGTYDLK.S	
89	1	508.3800	135.84	2	25.1	81.8	0	325-334	K.SVLGQLGITK.V	
100	1	918.0000	46.75	2	27.6	121.9	0	335-352	K.VFSNGADLSGVTEEAPLK.L	Deamidated: 4
91	2	917.5380	79.48	2	26.1	109.6	0	335-352	K.VFSNGADLSGVTEEAPLK.L	
98	1	1081.9930	-64.34	2	27.4	48.9	1	335-355	K.VFSNGADLSGVTEEAPLKLSK.A	Deamidated: 4

Protein 3: Haptoglobin OS=Homo sapiens GN=HP PE=1 SV=1
Accession: HPT_HUMAN **Score:** 58.55
Database: SwissProt_July2013 **MW [kDa]:** 45.20

**Seq. Coverage [%]:**	2.70 %	**pI:**	6.13
		**No. of Peptides:**	1

Cmpd.	No. of Cmpds.	m/z meas.	Δ m/z [ppm]	z	Rt [min]	Score	P	Range	Sequence	Modification
3	1	637.9080	134.19	2	14.6	58.5	1	312-322	R.HYEGSTVPEKK.T	

**Search Type**	Combined MS/MS - ProteinExtractor
**Search Result**	Amazon Human_SPJuly2013_2016-08-08 18:23:29
**Search Location**	/Kathir/ABC 2016 IgG And A1AT/KA A1AT ACN Spin E3_RE7_01_8316.d/

**Protein 1:**	Alpha-1-antitrypsin OS=Homo sapiens GN=SERPINA1 PE=1 SV=3		
**Accession:**	A1AT_HUMAN	**Score:**	310.41
**Database:**	SwissProt_July2013	**MW [kDa]:**	46.70
**Seq. Coverage [%]:**	13.90 %	**pI:**	5.27
		**No. of Peptides:**	5

Cmpd.	No. of Cmpds.	m/z meas.	Δ m/z [ppm]	z	Rt [min]	Score	P	Range	Sequence	Modification
5	2	890.4170	32.91	2	14.8	68.1	0	35-49	K.TDTSHHDQDHPTFNK.I	
72	1	445.3560	210.26	3	20.9	36.7	2	150-160	K.LVDKFLEDVKK.L	
24	1	536.6880	130.87	3	16.6	60.0	2	179-192	K.KQINDYVEKGTQGK.I	
39	1	426.8100	139.36	2	17.5	63.8	0	307-314	R.SASLHLPK.L	
82	1	508.4360	246.01	2	25.1	81.8	0	325-334	K.SVLGQLGITK.V	

**Protein 2:**	Serum albumin OS=Homo sapiens GN=ALB PE=1 SV=2		
**Accession:**	ALBU_HUMAN	**Score:**	159.82
**Database:**	SwissProt_July2013	**MW [kDa]:**	69.30
**Seq. Coverage [%]:**	5.60 %	**pI:**	5.88
		**No. of Peptides:**	3
**Modification(s):**	Carbamidomethyl		

Cmpd.	No. of Cmpds.	m/z meas.	Δ m/z [ppm]	z	Rt [min]	Score	P	Range	Sequence	Modification
2	1	575.3310	68.47	2	14.4	58.9	1	25-34	R.DAHKSEVAHR.F	
65	1	564.9690	205.40	2	20.2	45.0	1	549-558	K.KQTALVELVK.H	
19	1	543.3150	124.94	3	15.8	55.9	2	585-598	K.ADDKETCFAEEGKK.L	Carbamidomethyl: 7

Search Type Combined MS/MS - ProteinExtractor
Search Result Amazon Human_SPJuly2013_2016-08-08 18:23:29
Search Location /Kathir/ABC 2016 IgG And A1AT/KA IgG ACN Drop 1min E1_RA1_01_8261.d/

Protein 1: Ig gamma-1 chain C region OS=Homo sapiens GN=IGHG1 PE=1 SV=1
Accession: IGHG1_HUMAN **Score:** 1006.03
Database: SwissProt_July2013 **MW [kDa]:** 36.10
Seq. Coverage [%]: 53.60 % **pI:** 9.36
No. of Peptides: 14

Modification(s): Carbamidomethyl, Deamidated

Cmpd.	No. of Cmpds.	m/z meas.	Δ m/z [ppm]	z	Rt [min]	Score	P	Range	Sequence	Modification
143	2	593.8670	67.42	2	24.6	84.0	0	5-16	K.GPSVFPLAPSSK.S	
129	1	661.3930	76.13	2	22.5	99.0	0	17-30	K.STSGGTAALGCLVK.D	Carbamidomethyl: 11
169	1	1112.3070	79.22	3	27.8	83.6	1	102-131	K.SCDKTHTCPPCPAPELLGGPSVF LFPPKPK.D	Carbamidomethyl: 2, 8, 11
175	1	948.9150	95.88	3	28.7	76.8	0	106-131	K.THTCPPCPAPELLGGPSVFLFPP KPK.D	Carbamidomethyl: 4, 7
149	1	713.7280	66.33	3	25.1	77.6	0	139-157	R.TPEVTCVVVDVSHEDPEVK.F	Carbamidomethyl: 6
146	1	839.4790	88.60	2	24.8	79.6	0	158-171	K.FNWYVDGVEVHNAK.T	
126	1	643.9040	98.47	2	22.2	34.1	0	228-238	R.EPQVYTLPPSR.D	
131	1	937.0520	67.53	2	22.6	56.1	1	228-243	R.EPQVYTLPPSRDELTK.N	
138	1	581.3960	133.41	2	23.7	76.5	0	244-253	K.NQVSLTCLVK.G	Carbamidomethyl: 7
181	3	1272.5730	2.89	2	29.7	88.4	0	254-275	K.GFYPSDIAVEWESNGQPENNYK. T	
182	2	1273.0780	13.09	2	30.1	118.3	0	254-275	K.GFYPSDIAVEWESNGQPENNYK.	Deamidated: 14

									T	
184	2	1273.0660	3.67	2	30.6	88.1	0	254-275	K.GFYPSDIAVEWESNGQPENNYK.T	Deamidated: 19
183	1	937.5460	86.88	2	30.4	88.7	0	276-292	K.TTPPVLDSDGSFFLYSK.L	
136	1	934.4940	71.46	3	23.6	43.3	0	300-322	R.WQQGNVFSCSVMHEALHNHYTQK.S	Carbamidomethyl: 9

Protein 2: Ig kappa chain C region OS=Homo sapiens GN=IGKC PE=1 SV=1

Accession: IGKC_HUMAN **Score:** 978.45

Database: SwissProt_July2013 **MW [kDa]:** 11.60

Seq. Coverage [%]: 90.60 % **pI:** 5.53

No. of Peptides: 10

Modification(s): Carbamidomethyl, Deamidated

Cmpd.	No. of Cmpds.	m/z meas.	Δ m/z [ppm]	z	Rt [min]	Score	P	Range	Sequence	Modification
185	1	973.5860	70.76	2	30.9	92.8	0	1-18	-.TVAAPSVFIFPPSDEQLK.S	
187	1	899.5210	77.54	2	32.2	63.3	0	19-34	K.SGTASVVCLLNNFYPR.E	Carbamidomethyl: 8
127	1	893.1240	32.64	3	22.4	107.6	1	38-61	K.VQWKVDNALQSGNSQESVTEQDSK.D	
97	4	1068.9350	418.35	2	19.0	136.9	0	42-61	K.VDNALQSGNSQESVTEQDSK.D	
103	2	1069.0500	65.47	2	19.5	109.8	0	42-61	K.VDNALQSGNSQESVTEQDSK.D	Deamidated: 6
155	2	1207.5860	285.53	3	26.1	127.8	1	42-75	K.VDNALQSGNSQESVTEQDSKDSTYSLSSTLTLSK.A	
161	1	751.9630	106.58	2	27.0	103.1	0	62-75	K.DSTYSLSSTLTLSK.A	
45	1	445.7500	62.89	2	14.4	26.7	1	76-82	K.ADYEKHK.V	
92	1	1071.1520	100.80	2	18.4	138.7	1	81-99	K.HKVYACEVTHQGLSSPVTK.S	Carbamidomethyl: 6
108	1	626.0340	85.48	3	20.3	71.7	0	83-99	K.VYACEVTHQGLSSPVTK.S	Carbamidomethyl: 4

Protein 3: Ig gamma-2 chain C region OS=Homo sapiens GN=IGHG2 PE=1 SV=2

Accession: IGHG2_HUMAN **Score:** 478.09

Database: SwissProt_July2013 **MW [kDa]:** 35.90

Seq. Coverage [%]: 26.10 % **pI:** 8.80

No. of Peptides: 6

Modification(s): Carbamidomethyl, Oxidation

Accumulated Protein Report

Cmpd.	No. of Cmpds.	m/z meas.	Δ m/z [ppm]	z	Rt [min]	Score	P	Range	Sequence	Modification
152	1	644.3870	89.48	2	25.4	52.3	0	5-16	K.GPSVFPLAPCSR.S	Carbamidomethyl: 10
134	1	712.4140	77.91	2	23.2	77.6	0	17-30	R.STSESTAALGCLVK.D	Carbamidomethyl: 11
178	2	970.2120	75.51	3	29.3	68.8	0	102-127	K.CCVECPPCPAPPVAGPSVFLFPP KPK.D	Carbamidomethyl: 1, 2, 5, 8
112	4	961.0560	87.19	2	20.8	38.6	1	224-239	R.EPQVYTLPPSREEMTK.N	Oxidation: 14
123	1	953.0670	96.79	2	21.6	46.0	1	224-239	R.EPQVYTLPPSREEMTK.N	
180	1	961.5180	72.76	2	29.2	41.0	0	272-288	K.TTPPMLDSDGSFFLYSK.L	Oxidation: 5

Protein 4: Ig gamma-4 chain C region OS=Homo sapiens GN=IGHG4 PE=1 SV=1
Accession: IGHG4_HUMAN **Score:** 462.53
Database: SwissProt_July2013 **MW [kDa]:** 35.90
Seq. Coverage [%]: 4.30 % **pI:** 7.80
No. of Peptides: 1
Modification(s): Carbamidomethyl, Deamidated

Cmpd.	No. of Cmpds.	m/z meas.	Δ m/z [ppm]	z	Rt [min]	Score	P	Range	Sequence	Modification
48	1	832.9780	107.47	2	14.7	49.5	0	80-93	K.TYTCNVDHKPSNTK.V	Carbamidomethyl: 4

Protein 5: Ig gamma-3 chain C region OS=Homo sapiens GN=IGHG3 PE=1 SV=2
Accession: IGHG3_HUMAN **Score:** 407.07
Database: SwissProt_July2013 **MW [kDa]:** 41.30
Seq. Coverage [%]: 2.70 % **pI:** 9.41
No. of Peptides: 1
Modification(s): Carbamidomethyl, Oxidation

Cmpd.	No. of Cmpds.	m/z meas.	Δ m/z [ppm]	z	Rt [min]	Score	P	Range	Sequence	Modification
69	1	593.8090	94.96	2	16.4	60.6	0	119-128	K.SCDTPPPCPR.C	Carbamidomethyl: 2, 8

Protein 6: Ig lambda-2 chain C regions OS=Homo sapiens GN=IGLC2 PE=1 SV=1
Accession: LAC2_HUMAN **Score:** 357.87
Database: SwissProt_July2013 **MW [kDa]:** 11.30
Seq. Coverage [%]: 62.30 % **pI:** 7.73
No. of Peptides: 5

Modification(s): Carbamidomethyl

Cmpd.	No. of Cmpds.	m/z meas.	Δ m/z [ppm]	z	Rt [min]	Score	P	Range	Sequence	Modification
163	1	993.5830	70.90	2	27.2	85.8	0	5-23	K.AAPSVTLFPPSSEELQANK.A	
80	1	838.0260	113.53	2	17.3	54.4	1	44-60	K.ADSSPVKAGVETTTPSK.Q	
54	1	495.7990	81.20	2	15.3	69.6	0	51-60	K.AGVETTTPSK.Q	
172	1	872.5040	81.32	2	28.4	90.8	0	66-80	K.YAASSYLSLTPEQWK.S	
81	1	856.4490	76.80	2	17.4	57.3	0	84-98	R.SYSCQVTHEGSTVEK.T	Carbamidomethyl: 4

Protein 7: Ig lambda-1 chain C regions OS=Homo sapiens GN=IGLC1 PE=1 SV=1

**Accession:**	LAC1_HUMAN	**Score:**	208.52
**Database:**	SwissProt_July2013	**MW [kDa]:**	11.30
**Seq. Coverage [%]:**	17.90 %	**pI:**	9.16
		**No. of Peptides:**	1

Modification(s): Carbamidomethyl

Cmpd.	No. of Cmpds.	m/z meas.	Δ m/z [ppm]	z	Rt [min]	Score	P	Range	Sequence	Modification
164	1	1022.0700	45.70	2	27.2	60.5	0	5-23	K.ANPTVTLFPPSSEELQANK.A	

Protein 8: Ig kappa chain V-III region NG9 (Fragment) OS=Homo sapiens PE=1 SV=1

**Accession:**	KV303_HUMAN	**Score:**	86.39
**Database:**	SwissProt_July2013	**MW [kDa]:**	10.70
**Seq. Coverage [%]:**	22.00 %	**pI:**	7.08
		**No. of Peptides:**	1

Cmpd.	No. of Cmpds.	m/z meas.	Δ m/z [ppm]	z	Rt [min]	Score	P	Range	Sequence	Modification
139	1	813.7900	61.57	3	24.0	86.4	0	29-50	R.ASQSVSSSYLAWYQQKPGQAPR.L	

Protein 9: Ig heavy chain V-III region HIL OS=Homo sapiens PE=1 SV=1

**Accession:**	HV310_HUMAN	**Score:**	58.30
**Database:**	SwissProt_July2013	**MW [kDa]:**	13.60

Accumulated Protein Report

Seq. Coverage [%]: 13.20 %

pI: 9.93
No. of Peptides: 1

Cmpd.	No. of Cmpds.	m/z meas.	Δ m/z [ppm]	z	Rt [min]	Score	P	Range	Sequence	Modification
171	1	796.9910	32.39	2	28.4	58.3	1	1-16	-.QVKLVQAGGGVVQPGR.S	

Search Type Combined MS/MS - ProteinExtractor
Search Result Amazon Human_SPJuly2013_2016-08-08 18:23:29
Search Location /Kathir/ABC 2016 IgG And A1AT/KA IgG ACN Drop 1min E2_RA2_01_8248.d/

Protein 1: Ig gamma-1 chain C region OS=Homo sapiens GN=IGHG1 PE=1 SV=1
Accession: IGHG1_HUMAN
Database: SwissProt_July2013
Seq. Coverage [%]: 48.80 %

Score: 902.26
MW [kDa]: 36.10
pI: 9.36
No. of Peptides: 12

Modification(s): Carbamidomethyl, Oxidation, Deamidated

Cmpd.	No. of Cmpds.	m/z meas.	Δ m/z [ppm]	z	Rt [min]	Score	P	Range	Sequence	Modification
115	1	593.8660	65.74	2	24.6	67.2	0	5-16	K.GPSVFPLAPSSK.S	
107	1	661.3950	79.15	2	22.5	94.3	0	17-30	K.STSGGTAALGCLVK.D	Carbamidomethyl: 11
133	1	834.4310	17.99	4	27.6	63.9	1	102-131	K.SCDKTHTCPPCPAPELLGGPSVF LFPPKPK.D	Carbamidomethyl: 2, 8, 11
80	1	426.2710	123.87	2	19.9	44.3	0	132-138	K.DTLMISR.T	Oxidation: 4
119	1	713.7320	71.94	3	25.3	68.0	0	139-157	R.TPEVTCVVVDVSHEDPEVK.F	Carbamidomethyl: 6
116	1	839.4720	80.26	2	24.8	89.3	0	158-171	K.FNWYVDGVEVHNAK.T	
108	1	937.0620	78.20	2	22.7	42.3	1	228-243	R.EPQVYTLPPSRDELTK.N	
113	1	581.3960	133.41	2	23.8	79.3	0	244-253	K.NQVSLTCLVK.G	Carbamidomethyl: 7
166	2	1273.1070	35.87	2	30.2	102.6	0	254-275	K.GFYPSDIAVEWESNGQPENNYK. T	Deamidated: 14
172	2	1273.6070	428.63	2	30.6	55.4	0	254-275	K.GFYPSDIAVEWESNGQPENNYK. T	Deamidated: 19

162	2	1272.6060	28.82	2	29.7	85.0	0	254-275	K.GFYPSDIAVEWESNGQPENNYK.T	
169	1	937.5680	110.35	2	30.4	83.1	0	276-292	K.TTPPVLDSDGSFFLYSK.L	

Protein 2: Ig kappa chain C region OS=Homo sapiens GN=IGKC PE=1 SV=1

Accession: IGKC_HUMAN — **Score:** 749.97

Database: SwissProt_July2013 — **MW [kDa]:** 11.60

Seq. Coverage [%]: 75.50 % — **pI:** 5.53

No. of Peptides: 8

Modification(s): Carbamidomethyl, Deamidated

Cmpd.	No. of Cmpds.	m/z meas.	Δ m/z [ppm]	z	Rt [min]	Score	P	Range	Sequence	Modification
174	1	973.6200	105.68	2	31.0	82.7	0	1-18	-.TVAAPSVFIFPPSDEQLK.S	
105	1	893.1320	41.60	3	22.4	87.9	1	38-61	K.VQWKVDNALQSGNSQESVTEQDSK.D	
70	6	1068.8840	370.62	2	18.9	140.8	0	42-61	K.VDNALQSGNSQESVTEQDSK.D	
127	1	1207.2630	17.98	3	26.7	124.1	1	42-75	K.VDNALQSGNSQESVTEQDSKDSTYSLSSTLTLSK.A	
128	1	751.9720	118.55	2	27.0	92.2	0	62-75	K.DSTYSLSSTLTLSK.A	
17	1	445.7670	101.03	2	14.4	25.4	1	76-82	K.ADYEKHK.V	
64	1	1071.1560	104.54	2	18.3	113.0	1	81-99	K.HKVYACEVTHQGLSSPVTK.S	Carbamidomethyl: 6
84	1	938.5600	98.99	2	20.4	83.9	0	83-99	K.VYACEVTHQGLSSPVTK.S	Carbamidomethyl: 4

Protein 3: Ig gamma-4 chain C region OS=Homo sapiens GN=IGHG4 PE=1 SV=1

Accession: IGHG4_HUMAN — **Score:** 541.36

Database: SwissProt_July2013 — **MW [kDa]:** 35.90

Seq. Coverage [%]: 12.20 % — **pI:** 7.80

No. of Peptides: 3

Modification(s): Carbamidomethyl, Oxidation, Deamidated

Cmpd.	No. of Cmpds.	m/z meas.	Δ m/z [ppm]	z	Rt [min]	Score	P	Range	Sequence	Modification
120	1	644.3950	101.89	2	25.5	64.4	0	5-16	K.GPSVFPLAPCSR.S	Carbamidomethyl: 10
111	1	712.4210	87.74	2	23.2	64.8	0	17-30	R.STSESTAALGCLVK.D	Carbamidomethyl: 11
21	1	832.9680	95.47	2	14.6	36.2	0	80-93	K.TYTCNVDHKPSNTK.V	Carbamidomethyl: 4

Protein 4: Ig gamma-2 chain C region OS=Homo sapiens GN=IGHG2 PE=1 SV=2
Accession: IGHG2_HUMAN **Score:** 486.69
Database: SwissProt_July2013 **MW [kDa]:** 35.90
Seq. Coverage [%]: 18.10 % **pI:** 8.80
No. of Peptides: 4
Modification(s): Carbamidomethyl, Oxidation

Cmpd.	No. of Cmpds.	m/z meas.	Δ m/z [ppm]	z	Rt [min]	Score	P	Range	Sequence	Modification
155	1	970.1810	43.56	3	29.2	60.3	0	102-127	K.CCVECPPCPAPPVAGPSVFLFPP KPK.D	Carbamidomethyl: 1, 2, 5, 8
88	3	961.0620	93.43	2	20.8	56.2	1	224-239	R.EPQVYTLPPSREEMTK.N	Oxidation: 14
97	1	953.0730	103.09	2	21.7	34.0	1	224-239	R.EPQVYTLPPSREEMTK.N	
156	2	961.5300	85.24	2	29.3	65.2	0	272-288	K.TTPPMLDSDGSFFLYSK.L	Oxidation: 5

Protein 5: Ig lambda-2 chain C regions OS=Homo sapiens GN=IGLC2 PE=1 SV=1
Accession: LAC2_HUMAN **Score:** 375.35
Database: SwissProt_July2013 **MW [kDa]:** 11.30
Seq. Coverage [%]: 62.30 % **pI:** 7.73
No. of Peptides: 5
Modification(s): Carbamidomethyl

Cmpd.	No. of Cmpds.	m/z meas.	Δ m/z [ppm]	z	Rt [min]	Score	P	Range	Sequence	Modification
131	1	993.6030	91.03	2	27.3	78.2	0	5-23	K.AAPSVTLFPPSSEELQANK.A	
54	1	838.0160	101.59	2	17.4	51.9	1	44-60	K.ADSSPVKAGVETTTPSK.Q	
28	1	495.8010	85.23	2	15.3	73.2	0	51-60	K.AGVETTTPSK.Q	
143	1	872.5190	98.52	2	28.4	88.4	0	66-80	K.YAASSYLSLTPEQWK.S	
53	1	856.4630	93.14	2	17.3	83.6	0	84-98	R.SYSCQVTHEGSTVEK.T	Carbamidomethyl: 4

Protein 6: Immunoglobulin lambda-like polypeptide 5 OS=Homo sapiens GN=IGLL5 PE=2 SV=2
Accession: IGLL5_HUMAN **Score:** 347.56
Database: SwissProt_July2013 **MW [kDa]:** 23.00
Seq. Coverage [%]: 17.30 % **pI:** 10.03
No. of Peptides: 3
Modification(s): Carbamidomethyl

Cmpd.	No. of Cmpds.	m/z meas.	Δ m/z [ppm]	z	Rt [min]	Score	P	Range	Sequence	Modification
67	1	421.3090	114.59	2	18.3	39.0	0	105-112	K.VTVLGQPK.A	
130	2	1022.0870	62.33	2	27.2	65.6	0	113-131	K.ANPTVTLFPPSSEELQANK.A	
18	1	509.3480	128.83	2	14.5	70.9	0	159-168	K.AGVETTKPSK.Q	

Protein 7: Ig kappa chain V-I region Lay OS=Homo sapiens PE=1 SV=1
Accession: KV113_HUMAN
Database: SwissProt_July2013
Seq. Coverage [%]: 8.30 %

Score: 57.52
MW [kDa]: 11.80
pI: 9.17
No. of Peptides: 1

Cmpd.	No. of Cmpds.	m/z meas.	Δ m/z [ppm]	z	Rt [min]	Score	P	Range	Sequence	Modification
102	1	497.3470	114.59	2	22.1	57.5	0	46-54	K.LLIYGASTR.E	

Search Type Combined MS/MS - ProteinExtractor
Search Result Amazon Human_SPJuly2013_2016-08-08 18:23:29
Search Location /Kathir/ABC 2016 IgG And A1AT/KA A1AT EtOH Drop 1min E2_RD5_01_8282.d/

Protein 1: Serum albumin OS=Homo sapiens GN=ALB PE=1 SV=2
Accession: ALBU_HUMAN
Database: SwissProt_July2013
Seq. Coverage [%]: 54.80 %

Score: 1977.31
MW [kDa]: 69.30
pI: 5.88
No. of Peptides: 31

Modification(s): Carbamidomethyl, Oxidation

Cmpd.	No. of Cmpds.	m/z meas.	Δ m/z [ppm]	z	Rt [min]	Score	P	Range	Sequence	Modification
19	1	575.3600	118.88	2	14.4	58.9	1	25-34	R.DAHKSEVAHR.F	
69	2	613.8610	89.23	2	19.7	75.5	1	35-44	R.FKDLGEENFK.A	

107	1	575.3720	105.78	2	22.8	77.1	0	66-75	K.LVNEVTEFAK.T	
32	1	749.8450	69.54	2	16.1	112.3	0	76-88	K.TCVADESAENCDK.S	Carbamidomethyl: 2, 11
138	1	1137.9270	49.42	3	26.4	58.4	2	76-105	K.TCVADESAENCDKSLHTLFGDKL CTVATLR.E	Carbamidomethyl: 2, 11, 24
47	1	725.8310	87.08	2	17.4	64.1	0	106-117	R.ETYGEMADCCAK.Q	Carbamidomethyl: 9, 10; Oxidation: 6
41	1	572.3160	79.72	3	17.0	23.4	1	118-130	K.QEPERNECFLQHK.D	Carbamidomethyl: 8
71	1	659.8340	33.02	4	19.8	24.2	2	118-138	K.QEPERNECFLQHKDDNPNLPR.L	Carbamidomethyl: 8
54	1	470.7890	130.05	2	18.0	42.5	0	131-138	K.DDNPNLPR.L	
72	1	528.3660	129.01	2	19.8	48.4	1	161-168	K.KYLYEIAR.R	
97	1	464.3160	141.39	2	22.2	40.5	0	162-168	K.YLYEIAR.R	
31	1	506.9670	72.74	3	15.9	56.3	2	206-219	K.LDELRDEGKASSAK.Q	
60	1	1043.9650	40.80	2	18.5	80.5	0	265-281	K.VHTECCHGDLLECADDR.A	Carbamidomethyl: 5, 6, 13
74	2	862.4320	63.28	3	20.1	73.8	1	265-286	K.VHTECCHGDLLECADDRADLAK. Y	Carbamidomethyl: 5, 6, 13
55	1	722.3680	60.01	2	18.2	91.0	0	287-298	K.YICENQDSISSK.L	Carbamidomethyl: 3
43	1	773.9790	99.47	2	17.0	61.7	1	299-310	K.LKECCEKPLLEK.S	Carbamidomethyl: 4, 5
182	5	997.5440	92.92	3	30.8	117.9	0	311-337	K.SHCIAEVENDEMPADLPSLAADF VESK.D	Carbamidomethyl: 3; Oxidation: 12
190	1	992.2180	99.10	3	31.9	103.3	0	311-337	K.SHCIAEVENDEMPADLPSLAADF VESK.D	Carbamidomethyl: 3
27	1	599.3310	81.66	2	15.7	32.7	1	338-347	K.DVCKNYAEAK.D	Carbamidomethyl: 3
124	1	490.0350	168.31	3	24.6	67.8	1	361-372	R.RHPDYSVVLLLR.L	
176	1	682.4540	123.14	3	29.6	62.3	0	397-413	K.VFDEFKPLVEEPQNLIK.Q	
148	1	829.4810	121.82	2	27.0	80.9	0	414-426	K.QNCELFEQLGEYK.F	Carbamidomethyl: 3
106	1	820.5490	93.22	2	22.7	78.0	1	438-452	K.KVPQVSTPTLVEVSR.N	
23	1	501.8260	86.58	2	15.1	28.8	1	491-499	K.TPVSDRVTK.C	
49	1	569.7840	55.08	2	17.7	55.4	0	500-508	K.CCTESLVNR.R	Carbamidomethyl: 1, 2
128	1	637.7290	125.86	3	25.2	58.3	0	509-524	R.RPCFSALEVDETYVPK.E	Carbamidomethyl: 3
145	1	849.1200	70.30	3	26.8	57.4	1	525-545	K.EFNAETFTFHADICTLSEKER.Q	Carbamidomethyl: 14
77	1	564.9180	115.11	2	20.2	63.1	1	549-558	K.KQTALVELVK.H	
118	1	500.8940	176.72	2	23.7	49.2	0	550-558	K.QTALVELVK.H	
40	1	750.3680	64.55	2	16.9	61.6	1	585-597	K.ADDKETCFAEEGK.K	Carbamidomethyl: 7
29	1	814.4140	57.65	2	15.8	72.0	2	585-598	K.ADDKETCFAEEGKK.L	Carbamidomethyl: 7

Protein 2: Alpha-1-antitrypsin OS=Homo sapiens GN=SERPINA1 PE=1 SV=3
Accession: A1AT_HUMAN **Score:** 1586.31

Accumulated Protein Report

Database: SwissProt_July2013
Seq. Coverage [%]: 50.70 %
MW [kDa]: 46.70
pI: 5.27
No. of Peptides: 26
Modification(s): Carbamidomethyl, Oxidation, Deamidated

Cmpd.	No. of Cmpds.	m/z meas.	Δ m/z [ppm]	z	Rt [min]	Score	P	Range	Sequence	Modification
21	1	593.9640	61.36	3	14.8	54.3	0	35-49	K.TDTSHHDQDHPTFNK.I	
173	1	1135.2810	67.72	3	29.4	39.3	1	35-63	K.TDTSHHDQDHPTFNKITPNLAEF AFSLYR.Q	Deamidated: 14
111	1	603.4250	136.83	2	23.1	39.1	1	150-159	K.LVDKFLEDVK.K	
85	1	667.4540	96.00	2	21.0	64.7	2	150-160	K.LVDKFLEDVKK.L	
88	1	772.1100	77.91	3	21.2	99.6	2	160-179	K.KLYHSEAFTVNFGDTEEAKK.Q	
125	1	686.7310	113.29	3	24.6	49.1	0	161-178	K.LYHSEAFTVNFGDTEEAK.K	
104	2	729.4010	67.83	3	22.7	63.0	1	161-179	K.LYHSEAFTVNFGDTEEAKK.Q	
38	1	568.8610	105.58	2	16.8	68.4	1	179-187	K.KQINDYVEK.G	
37	1	804.4880	80.78	2	16.6	72.4	2	179-192	K.KQINDYVEKGTQGK.I	
56	1	504.7960	84.27	2	18.2	40.5	0	180-187	K.QINDYVEK.G	
51	1	740.4220	62.76	2	17.7	35.1	1	180-192	K.QINDYVEKGTQGK.I	
63	1	638.3690	36.26	2	18.6	41.0	1	216-225	K.GKWERPFEVK.D	
78	1	545.8550	123.42	2	20.4	31.8	0	218-225	K.WERPFEVK.D	
110	1	946.5120	85.13	2	23.1	83.8	0	226-241	K.DTEEEDFHVDQVTTVK.V	
50	1	710.4180	91.74	2	17.7	29.9	1	247-257	K.RLGMFNIQHCK.K	Carbamidomethyl: 10; Oxidation: 4
45	1	464.6170	103.47	3	17.2	30.3	1	248-258	R.LGMFNIQHCKK.L	Carbamidomethyl: 9; Oxidation: 3
101	1	902.5780	104.59	2	22.5	78.9	0	284-298	K.LQHLENELTHDIITK.F	
35	1	539.8020	64.03	2	16.4	30.3	1	299-306	K.FLENEDRR.S	
48	1	426.7770	62.03	2	17.6	54.9	0	307-314	R.SASLHLPK.L	
121	1	555.8900	151.68	2	24.2	78.8	0	315-324	K.LSITGTYDLK.S	
126	1	508.3890	153.55	2	25.1	81.8	0	325-334	K.SVLGQLGITK.V	
156	1	918.0590	111.02	2	27.6	129.3	0	335-352	K.VFSNGADLSGVTEEAPLK.L	Deamidated: 4
134	2	917.5520	94.74	2	26.1	114.5	0	335-352	K.VFSNGADLSGVTEEAPLK.L	
153	3	1082.1920	119.57	2	27.4	61.1	1	335-355	K.VFSNGADLSGVTEEAPLKLSK.A	Deamidated: 4
73	1	444.7930	84.37	2	19.9	48.9	0	360-367	K.AVLTIDEK.G	

193	1	1146.6390	497.76	2	32.2	36.1	0	368-389	K.GTEAAGAMFLEAIPMSIPPEVK.F	Oxidation: 8, 15

Protein 3: Haptoglobin OS=Homo sapiens GN=HP PE=1 SV=1
Accession: HPT_HUMAN
Database: SwissProt_July2013
Seq. Coverage [%]: 2.70 %
Score: 56.88
MW [kDa]: 45.20
pI: 6.13
No. of Peptides: 1

Cmpd.	No. of Cmpds.	m/z meas.	Δ m/z [ppm]	z	Rt [min]	Score	P	Range	Sequence	Modification
20	1	637.9010	123.22	2	14.6	56.9	1	312-322	R.HYEGSTVPEKK.T	

Search Type Combined MS/MS - ProteinExtractor
Search Result Amazon Human_SPJuly2013_2016-08-08 18:23:29
Search Location /Kathir/ABC 2016 IgG And A1AT/KA A1AT EtOH Drop 1min E2_RD5_01_8299.d/

Protein 1: Serum albumin OS=Homo sapiens GN=ALB PE=1 SV=2
Accession: ALBU_HUMAN
Database: SwissProt_July2013
Seq. Coverage [%]: 45.60 %
Score: 1645.74
MW [kDa]: 69.30
pI: 5.88
No. of Peptides: 27
Modification(s): Carbamidomethyl, Oxidation

Cmpd.	No. of Cmpds.	m/z meas.	Δ m/z [ppm]	z	Rt [min]	Score	P	Range	Sequence	Modification
15	1	575.3710	138.00	2	14.4	68.1	1	25-34	R.DAHKSEVAHR.F	
70	3	613.8540	77.83	2	19.7	75.5	1	35-44	R.FKDLGEENFK.A	
102	1	575.3790	117.94	2	22.9	75.4	0	66-75	K.LVNEVTEFAK.T	
31	1	749.8460	70.87	2	16.1	110.7	0	76-88	K.TCVADESAENCDK.S	Carbamidomethyl: 2, 11
45	1	725.8260	80.19	2	17.4	59.8	0	106-117	R.ETYGEMADCCAK.Q	Carbamidomethyl: 9, 10; Oxidation: 6
39	1	572.3160	79.72	3	17.0	23.5	1	118-130	K.QEPERNECFLQHK.D	Carbamidomethyl: 8

68	1	879.4620	54.74	3	19.3	36.4	2	118-138	K.QEPERNECFLQHKDDNPNLPR.L	Carbamidomethyl: 8
53	1	470.8180	191.66	2	18.1	35.8	0	131-138	K.DDNPNLPR.L	
72	1	528.3590	115.76	2	19.9	40.9	1	161-168	K.KYLYEIAR.R	
95	1	464.3190	147.85	2	22.3	48.0	0	162-168	K.YLYEIAR.R	
29	1	759.9500	76.92	2	15.9	67.4	2	206-219	K.LDELRDEGKASSAK.Q	
80	1	418.2590	83.87	3	20.6	47.9	1	247-257	R.FPKAEFAEVSK.L	
58	1	696.2910	10.01	3	18.6	61.8	0	265-281	K.VHTECCHGDLLECADDR.A	Carbamidomethyl: 5, 6, 13
75	1	862.4330	64.44	3	20.1	61.4	1	265-286	K.VHTECCHGDLLECADDRADLAK.Y	Carbamidomethyl: 5, 6, 13
54	1	722.3710	64.16	2	18.3	74.8	0	287-298	K.YICENQDSISSK.L	Carbamidomethyl: 3
41	1	773.9930	117.56	2	17.0	61.5	1	299-310	K.LKECCEKPLLEK.S	Carbamidomethyl: 4, 5
179	1	992.1960	76.93	3	32.0	107.4	0	311-337	K.SHCIAEVENDEMPADLPSLAADFVESK.D	Carbamidomethyl: 3
26	1	599.3340	86.67	2	15.8	37.2	1	338-347	K.DVCKNYAEAK.D	Carbamidomethyl: 3
139	1	829.4760	115.79	2	27.1	85.7	0	414-426	K.QNCELFEQLGEYK.F	Carbamidomethyl: 3
99	1	480.8570	149.96	2	22.6	67.5	0	427-434	K.FQNALLVR.Y	
101	1	820.5510	95.66	2	22.8	83.9	1	438-452	K.KVPQVSTPTLVEVSR.N	
14	1	515.3140	155.54	2	14.4	33.5	1	461-468	K.CCKHPEAK.R	Carbamidomethyl: 1, 2
19	1	501.8420	118.47	2	15.1	37.0	1	491-499	K.TPVSDRVTK.C	
47	1	569.7920	69.13	2	17.7	50.2	0	500-508	K.CCTESLVNR.R	Carbamidomethyl: 1, 2
76	2	564.9240	125.73	2	20.2	77.1	1	549-558	K.KQTALVELVK.H	
109	1	500.8830	154.75	2	23.8	58.3	0	550-558	K.QTALVELVK.H	
27	1	543.2780	56.83	3	15.8	59.2	2	585-598	K.ADDKETCFAEEGKK.L	Carbamidomethyl: 7

Protein 2: Alpha-1-antitrypsin OS=Homo sapiens GN=SERPINA1 PE=1 SV=3

**Accession:**	A1AT_HUMAN	**Score:**	1098.81
**Database:**	SwissProt_July2013	**MW [kDa]:**	46.70
**Seq. Coverage [%]:**	37.10 %	**pI:**	5.27
		**No. of Peptides:**	19

Modification(s): Carbamidomethyl, Oxidation, Deamidated

Cmpd.	No. of Cmpds.	m/z meas.	Δ m/z [ppm]	z	Rt [min]	Score	P	Range	Sequence	Modification
22	1	890.4660	87.94	2	15.5	49.3	0	35-49	K.TDTSHHDQDHPTFNK.I	
84	1	667.4710	121.48	2	20.9	69.6	2	150-160	K.LVDKFLEDVKK.L	
38	1	568.8550	95.03	2	16.8	73.2	1	179-187	K.KQINDYVEK.G	

36	1	804.4970	91.97	2	16.7	81.2	2	179-192	K.KQINDYVEKGTQGK.I	
55	1	504.7990	90.21	2	18.3	43.2	0	180-187	K.QINDYVEK.G	
49	1	740.4250	66.81	2	17.8	38.6	1	180-192	K.QINDYVEKGTQGK.I	
60	1	638.3690	36.26	2	18.7	54.7	1	216-225	K.GKWERPFEVK.D	
78	1	545.8530	119.75	2	20.4	34.4	0	218-225	K.WERPFEVK.D	
104	1	946.5290	103.09	2	23.2	97.4	0	226-241	K.DTEEEDFHVDQVTTVK.V	
51	1	473.9550	106.96	3	18.0	27.0	1	247-257	K.RLGMFNIQHCK.K	Carbamidomethyl: 10; Oxidation: 4
62	1	632.3690	105.53	2	19.1	41.7	0	248-257	R.LGMFNIQHCK.K	Carbamidomethyl: 9; Oxidation: 3
43	1	464.6200	109.93	3	17.2	21.7	1	248-258	R.LGMFNIQHCKK.L	Carbamidomethyl: 9; Oxidation: 3
98	1	602.0400	80.57	3	22.6	50.0	0	284-298	K.LQHLENELTHDIITK.F	
34	1	539.8030	65.89	2	16.4	32.6	1	299-306	K.FLENEDRR.S	
46	1	426.7700	45.63	2	17.6	48.8	0	307-314	R.SASLHLPK.L	
110	1	555.9050	178.67	2	24.3	79.3	0	315-324	K.LSITGTYDLK.S	
118	1	508.4080	190.93	2	25.2	81.6	0	325-334	K.SVLGQLGITK.V	
148	1	918.0700	123.00	2	27.7	125.1	0	335-352	K.VFSNGADLSGVTEEAPLK.L	Deamidated: 4
73	1	444.8080	118.10	2	20.0	49.4	0	360-367	K.AVLTIDEK.G	

Protein 3: Ig kappa chain C region OS=Homo sapiens GN=IGKC PE=1 SV=1
Accession: IGKC_HUMAN
Database: SwissProt_July2013
Seq. Coverage [%]: 32.10 %
Score: 128.24
MW [kDa]: 11.60
pI: 5.53
No. of Peptides: 1

Cmpd.	No. of Cmpds.	m/z meas.	Δ m/z [ppm]	z	Rt [min]	Score	P	Range	Sequence	Modification
130	1	1207.2820	33.72	3	26.1	128.2	1	42-75	K.VDNALQSGNSQESVTEQDSKDST YSLSSTLTLSK.A	

Protein 4: Ig gamma-1 chain C region OS=Homo sapiens GN=IGHG1 PE=1 SV=1
Accession: IGHG1_HUMAN
Database: SwissProt_July2013
Seq. Coverage [%]: 6.70 %
Score: 126.88
MW [kDa]: 36.10
pI: 9.36

No. of Peptides:	1
Modification(s):	Carbamidomethyl, Deamidated

Cmpd.	No. of Cmpds.	m/z meas.	Δ m/z [ppm]	z	Rt [min]	Score	P	Range	Sequence	Modification
170	1	1273.0750	10.74	2	30.3	102.2	0	254-275	K.GFYPSDIAVEWESNGQPENNYK.T	Deamidated: 14

**Protein 5:**	Haptoglobin OS=Homo sapiens GN=HP PE=1 SV=1		
**Accession:**	HPT_HUMAN	**Score:**	48.84
**Database:**	SwissProt_July2013	**MW [kDa]:**	45.20
**Seq. Coverage [%]:**	2.70 %	**pI:**	6.13
		**No. of Peptides:**	1

Cmpd.	No. of Cmpds.	m/z meas.	Δ m/z [ppm]	z	Rt [min]	Score	P	Range	Sequence	Modification
16	1	637.9110	138.90	2	14.7	48.8	1	312-322	R.HYEGSTVPEKK.T	

**Search Type**	Combined MS/MS - ProteinExtractor
**Search Result**	Amazon Human_SPJuly2013_2016-08-08 18:23:29
**Search Location**	/Kathir/ABC 2016 IgG And A1AT/KA A1AT EtOH Drop 1min E3_RD6_01_8283.d/

**Protein 1:**	Serum albumin OS=Homo sapiens GN=ALB PE=1 SV=2		
**Accession:**	ALBU_HUMAN	**Score:**	2114.60
**Database:**	SwissProt_July2013	**MW [kDa]:**	69.30
**Seq. Coverage [%]:**	56.70 %	**pI:**	5.88
		**No. of Peptides:**	34
**Modification(s):**	Carbamidomethyl, Oxidation, Deamidated		

Cmpd.	No. of Cmpds.	m/z meas.	Δ m/z [ppm]	z	Rt [min]	Score	P	Range	Sequence	Modification
9	1	575.3510	103.23	2	14.5	58.3	1	25-34	R.DAHKSEVAHR.F	

63	2	613.8680	100.64	2	19.7	75.5	1	35-44	R.FKDLGEENFK.A	
104	1	575.3760	112.73	2	22.9	72.0	0	66-75	K.LVNEVTEFAK.T	
23	1	749.8490	74.87	2	16.0	106.7	0	76-88	K.TCVADESAENCDK.S	Carbamidomethyl: 2, 11
136	1	1138.2820	361.40	3	26.3	51.6	2	76-105	K.TCVADESAENCDKSLHTLFGDKL CTVATLR.E	Carbamidomethyl: 2, 11, 24
38	1	725.8180	69.16	2	17.2	66.1	0	106-117	R.ETYGEMADCCAK.Q	Carbamidomethyl: 9, 10; Oxidation: 6
33	1	572.3160	79.72	3	16.9	27.1	1	118-130	K.QEPERNECFLQHK.D	Carbamidomethyl: 8
67	4	659.8570	67.88	4	19.9	30.6	2	118-138	K.QEPERNECFLQHKDDNPNLPR.L	Carbamidomethyl: 8
61	1	666.3630	72.57	3	19.5	52.2	1	123-138	R.NECFLQHKDDNPNLPR.L	Carbamidomethyl: 3
47	1	470.7870	125.80	2	18.0	52.2	0	131-138	K.DDNPNLPR.L	
66	1	528.3550	108.19	2	19.8	48.3	1	161-168	K.KYLYEIAR.R	
94	1	464.3210	152.16	2	22.2	48.0	0	162-168	K.YLYEIAR.R	
21	1	759.9530	80.86	2	15.9	58.5	2	206-219	K.LDELRDEGKASSAK.Q	
52	1	696.3040	28.68	3	18.5	76.6	0	265-281	K.VHTECCHGDLLECADDR.A	Carbamidomethyl: 5, 6, 13
70	2	862.4450	78.36	3	20.1	60.7	1	265-286	K.VHTECCHGDLLECADDRADLAK. Y	Carbamidomethyl: 5, 6, 13
48	1	722.3780	73.85	2	18.2	94.7	0	287-298	K.YICENQDSISSK.L	Carbamidomethyl: 3
35	1	773.9810	102.06	2	17.0	53.4	1	299-310	K.LKECCEKPLLEK.S	Carbamidomethyl: 4, 5
186	4	997.5490	97.93	3	30.7	100.3	0	311-337	K.SHCIAEVENDEMPADLPSLAADF VESK.D	Carbamidomethyl: 3; Oxidation: 12
194	1	992.2120	93.06	3	31.9	121.2	0	311-337	K.SHCIAEVENDEMPADLPSLAADF VESK.D	Carbamidomethyl: 3
18	1	599.3160	56.63	2	15.8	32.0	1	338-347	K.DVCKNYAEAK.D	Carbamidomethyl: 3
120	1	490.0390	176.47	3	24.6	70.4	1	361-372	R.RHPDYSVVLLLR.L	
182	1	1023.1810	126.76	2	29.7	68.8	0	397-413	K.VFDEFKPLVEEPQNLIK.Q	
181	1	682.7900	134.79	3	29.6	47.5	0	397-413	K.VFDEFKPLVEEPQNLIK.Q	Deamidated: 13
144	1	829.4760	115.79	2	27.0	88.6	0	414-426	K.QNCELFEQLGEYK.F	Carbamidomethyl: 3
103	1	820.5430	85.91	2	22.7	81.3	1	438-452	K.KVPQVSTPTLVEVSR.N	
13	1	501.8320	98.54	2	15.0	35.6	1	491-499	K.TPVSDRVTK.C	
41	1	569.7940	72.64	2	17.6	47.0	0	500-508	K.CCTESLVNR.R	Carbamidomethyl: 1, 2
125	1	637.7220	114.89	3	25.3	78.7	0	509-524	R.RPCFSALEVDETYVPK.E	Carbamidomethyl: 3
143	1	849.1570	113.88	3	26.8	75.0	1	525-545	K.EFNAETFTFHADICTLSEKER.Q	Carbamidomethyl: 14
73	1	564.9190	116.88	2	20.2	68.4	1	549-558	K.KQTALVELVK.H	
113	1	500.8720	132.79	2	23.7	52.7	0	550-558	K.QTALVELVK.H	
32	1	750.3710	68.54	2	16.8	60.1	1	585-597	K.ADDKETCFAEEGK.K	Carbamidomethyl: 7
19	1	543.2810	62.35	3	15.8	69.7	2	585-598	K.ADDKETCFAEEGKK.L	Carbamidomethyl: 7

157	1	1013.7670	165.77	1	28.0	32.5	0	599-609	K.LVAASQAALGL.-	

Protein 2: Alpha-1-antitrypsin OS=Homo sapiens GN=SERPINA1 PE=1 SV=3
Accession: A1AT_HUMAN
Database: SwissProt_July2013
Seq. Coverage [%]: 52.90 %
Score: 1878.47
MW [kDa]: 46.70
pI: 5.27
No. of Peptides: 28

Modification(s): Carbamidomethyl, Oxidation, Deamidated

Cmpd.	No. of Cmpds.	m/z meas.	Δ m/z [ppm]	z	Rt [min]	Score	P	Range	Sequence	Modification
11	1	593.9650	63.04	3	14.8	53.5	0	35-49	K.TDTSHHDQDHPTFNK.I	
177	1	851.4810	84.68	4	29.4	46.5	1	35-63	K.TDTSHHDQDHPTFNKITPNLAEF AFSLYR.Q	
195	1	1288.2210	42.59	2	32.2	124.3	0	126-149	R.TLNQPDSQLQLTTGNGLFLSEGL K.L	Deamidated: 15
108	1	603.4150	120.25	2	23.3	41.0	1	150-159	K.LVDKFLEDVK.K	
82	1	667.4640	110.99	2	20.9	71.3	2	150-160	K.LVDKFLEDVKK.L	
86	1	772.1180	88.27	3	21.2	95.1	2	160-179	K.KLYHSEAFTVNFGDTEEAKK.Q	
121	1	686.7310	113.29	3	24.7	62.9	0	161-178	K.LYHSEAFTVNFGDTEEAK.K	
100	1	729.3990	65.08	3	22.6	65.5	1	161-179	K.LYHSEAFTVNFGDTEEAKK.Q	
31	1	568.8620	107.34	2	16.8	68.4	1	179-187	K.KQINDYVEK.G	
29	1	804.5020	98.18	2	16.6	90.1	2	179-192	K.KQINDYVEKGTQGK.I	
49	1	504.8070	106.06	2	18.2	40.9	0	180-187	K.QINDYVEK.G	
42	1	740.4250	66.81	2	17.7	40.5	1	180-192	K.QINDYVEKGTQGK.I	
54	1	638.3730	42.52	2	18.7	43.9	1	216-225	K.GKWERPFEVK.D	
112	1	1050.2620	79.12	3	23.7	114.6	2	216-241	K.GKWERPFEVKDTEEEDFHVDQVT TVK.V	
76	1	545.8510	116.09	2	20.4	34.4	0	218-225	K.WERPFEVK.D	
106	1	946.5000	72.45	2	23.1	88.4	0	226-241	K.DTEEEDFHVDQVTTVK.V	
45	1	473.9490	94.30	3	17.8	23.3	1	247-257	K.RLGMFNIQHCK.K	Carbamidomethyl: 10; Oxidation: 4
37	1	464.6230	116.39	3	17.1	26.4	1	248-258	R.LGMFNIQHCKK.L	Carbamidomethyl: 9; Oxidation: 3
99	1	902.5740	100.16	2	22.6	105.2	0	284-298	K.LQHLENELTHDIITK.F	
26	1	539.7990	58.47	2	16.3	33.8	1	299-306	K.FLENEDRR.S	

40	1	426.7840	78.44	2	17.6	46.8	0	307-314	R.SASLHLPK.L	
117	1	555.8970	164.27	2	24.2	81.2	0	315-324	K.LSITGTYDLK.S	
122	1	508.4020	179.12	2	25.1	78.7	0	325-334	K.SVLGQLGITK.V	
155	1	918.0500	101.22	2	27.6	121.6	0	335-352	K.VFSNGADLSGVTEEAPLK.L	Deamidated: 4
134	2	917.5630	106.73	2	26.2	113.8	0	335-352	K.VFSNGADLSGVTEEAPLK.L	
135	1	721.4780	131.93	3	26.2	68.1	1	335-355	K.VFSNGADLSGVTEEAPLKLSK.A	
154	1	1082.1870	114.95	2	27.5	58.1	1	335-355	K.VFSNGADLSGVTEEAPLKLSK.A	Deamidated: 4
156	1	624.7460	133.95	3	27.8	39.9	0	390-404	K.FNKPFVFLMIEQNTK.S	Oxidation: 9

Protein 3: Haptoglobin OS=Homo sapiens GN=HP PE=1 SV=1
Accession: HPT_HUMAN
Database: SwissProt_July2013
Seq. Coverage [%]: 2.70 %

Score: 56.94
MW [kDa]: 45.20
pI: 6.13
No. of Peptides: 1

Cmpd.	No. of Cmpds.	m/z meas.	Δ m/z [ppm]	z	Rt [min]	Score	P	Range	Sequence	Modification
10	1	637.8810	91.86	2	14.6	56.9	1	312-322	R.HYEGSTVPEKK.T	

Search Type Combined MS/MS - ProteinExtractor
Search Result Amazon Human_SPJuly2013_2016-08-08 18:23:29
Search Location /Kathir/ABC 2016 IgG And A1AT/KA A1AT ISo Drop 1min E1_RC6_01_8275.d/

Protein 1: Alpha-1-antitrypsin OS=Homo sapiens GN=SERPINA1 PE=1 SV=3
Accession: A1AT_HUMAN
Database: SwissProt_July2013
Seq. Coverage [%]: 61.20 %

Score: 2109.44
MW [kDa]: 46.70
pI: 5.27
No. of Peptides: 34

Modification(s): Carbamidomethyl, Oxidation, Deamidated

Cmpd.	No. of Cmpds.	m/z meas.	Δ m/z [ppm]	z	Rt [min]	Score	P	Range	Sequence	Modification

5	1	890.4240	40.77	2	14.8	57.1	0	35-49	K.TDTSHHDQDHPTFNK.I	
214	1	821.5330	118.82	2	33.9	89.8	0	50-63	K.ITPNLAEFAFSLYR.Q	
198	1	1288.2100	34.05	2	32.1	135.7	0	126-149	R.TLNQPDSQLQLTTGNGLFLSEGL K.L	Deamidated: 15
199	1	859.1410	32.38	3	32.2	29.5	0	126-149	R.TLNQPDSQLQLTTGNGLFLSEGL K.L	Deamidated: 3
189	2	1287.7170	33.30	2	31.2	123.5	0	126-149	R.TLNQPDSQLQLTTGNGLFLSEGL K.L	
96	1	603.4080	108.65	2	23.1	34.1	1	150-159	K.LVDKFLEDVK.K	
72	1	667.4140	36.07	2	21.0	73.1	2	150-160	K.LVDKFLEDVKK.L	
77	4	772.1070	74.02	3	21.3	93.1	2	160-179	K.KLYHSEAFTVNFGDTEEAKK.Q	
117	1	686.7180	94.36	3	24.7	55.8	0	161-178	K.LYHSEAFTVNFGDTEEAK.K	
90	2	729.3820	41.78	3	22.6	53.9	1	161-179	K.LYHSEAFTVNFGDTEEAKK.Q	
24	1	568.8590	102.06	2	16.8	70.8	1	179-187	K.KQINDYVEK.G	
21	1	536.6650	88.01	3	16.6	54.3	2	179-192	K.KQINDYVEKGTQGK.I	
41	1	504.8040	100.12	2	18.2	40.5	0	180-187	K.QINDYVEK.G	
35	1	740.4000	33.04	2	17.8	45.7	1	180-192	K.QINDYVEKGTQGK.I	
48	1	638.3650	29.99	2	18.7	46.1	1	216-225	K.GKWERPFEVK.D	
105	1	787.9450	74.88	4	23.7	62.0	2	216-241	K.GKWERPFEVKDTEEEDFHVDQVT TVK.V	
65	1	545.8490	112.42	2	20.4	32.8	0	218-225	K.WERPFEVK.D	
94	1	946.4960	68.22	2	23.1	88.3	0	226-241	K.DTEEEDFHVDQVTTVK.V	
103	2	837.4510	77.99	3	23.6	77.3	1	226-246	K.DTEEEDFHVDQVTTVKVPMMK.R	Oxidation: 19, 20
87	1	889.4940	83.88	3	22.3	50.9	2	226-247	K.DTEEEDFHVDQVTTVKVPMMKR. L	Oxidation: 19, 20
37	2	473.9550	106.96	3	18.0	21.9	1	247-257	K.RLGMFNIQHCK.K	Carbamidomethyl: 10; Oxidation: 4
30	1	464.6210	112.08	3	17.2	31.2	1	248-258	R.LGMFNIQHCKK.L	Carbamidomethyl: 9; Oxidation: 3
89	1	602.0440	87.22	3	22.6	63.5	0	284-298	K.LQHLENELTHDIITK.F	
19	1	539.7990	58.47	2	16.4	31.1	1	299-306	K.FLENEDRR.S	
32	1	426.7620	26.88	2	17.6	58.5	0	307-314	R.SASLHLPK.L	
110	1	555.8710	117.49	2	24.2	83.2	0	315-324	K.LSITGTYDLK.S	
120	1	508.3850	145.68	2	25.1	81.8	0	325-334	K.SVLGQLGITK.V	
155	1	918.0400	90.32	2	27.7	118.8	0	335-352	K.VFSNGADLSGVTEEAPLK.L	Deamidated: 4
153	1	1082.1790	107.56	2	27.5	63.0	1	335-355	K.VFSNGADLSGVTEEAPLKLSK.A	Deamidated: 4

135	2	721.4630	111.13	3	26.3	55.3	1	335-355	K.VFSNGADLSGVTEEAPLKLSK.A	
200	1	1054.2790	65.40	3	32.2	47.5	1	360-389	K.AVLTIDEKGTEAAGAMFLEAIPM SIPPEVK.F	Oxidation: 16, 23
192	2	1146.1320	55.38	2	31.4	69.0	0	368-389	K.GTEAAGAMFLEAIPMSIPPEVK. F	Oxidation: 8, 15
151	1	936.6050	122.96	2	27.3	71.0	0	390-404	K.FNKPFVFLMIEQNTK.S	Oxidation: 9
152	1	620.0590	116.62	3	27.3	28.9	0	390-404	K.FNKPFVFLMIEQNTK.S	Deamidated: 12, 13

Protein 2: Serum albumin OS=Homo sapiens GN=ALB PE=1 SV=2
Accession: ALBU_HUMAN
Database: SwissProt_July2013
Seq. Coverage [%]: 63.40 %
Score: 2076.92
MW [kDa]: 69.30
pI: 5.88
No. of Peptides: 33
Modification(s): Carbamidomethyl, Oxidation, Deamidated

Cmpd.	No. of Cmpds.	m/z meas.	Δ m/z [ppm]	z	Rt [min]	Score	P	Range	Sequence	Modification
1	1	575.3760	146.69	2	14.4	58.9	1	25-34	R.DAHKSEVAHR.F	
56	1	613.8590	85.98	2	19.8	75.4	1	35-44	R.FKDLGEENFK.A	
15	1	749.8370	58.87	2	16.1	102.5	0	76-88	K.TCVADESAENCDK.S	Carbamidomethyl: 2, 11
138	2	1138.2430	38.86	3	26.6	62.7	2	76-105	K.TCVADESAENCDKSLHTLFGDKL CTVATLR.E	Carbamidomethyl: 2, 11, 24; Deamidated: 10
20	1	725.8110	59.52	2	16.5	59.7	0	106-117	R.ETYGEMADCCAK.Q	Carbamidomethyl: 9, 10; Oxidation: 6
29	1	572.3250	95.45	3	17.2	25.2	1	118-130	K.QEPERNECFLQHK.D	Carbamidomethyl: 8
54	1	879.4650	58.15	3	19.5	22.7	2	118-138	K.QEPERNECFLQHKDDNPNLPR.L	Carbamidomethyl: 8
38	1	470.7700	89.69	2	18.0	49.0	0	131-138	K.DDNPNLPR.L	
106	3	699.4130	98.77	4	23.8	49.3	1	139-161	R.LVRPEVDVMCTAFHDNEETFLKK .Y	Carbamidomethyl: 10; Oxidation: 9
118	1	699.7710	258.83	4	24.8	28.7	1	139-161	R.LVRPEVDVMCTAFHDNEETFLKK .Y	Carbamidomethyl: 10; Oxidation: 9; Deamidated: 16
57	1	528.3570	111.98	2	19.9	37.8	1	161-168	K.KYLYEIAR.R	
176	1	633.7640	148.47	3	29.5	76.4	1	169-183	R.RHPYFYAPELLFFAK.R	
115	1	709.0670	93.54	3	24.6	74.9	1	187-205	K.AAFTECCQAADKAACLLPK.L	Carbamidomethyl: 6, 7, 15
13	1	759.9500	76.92	2	15.9	55.5	2	206-219	K.LDELRDEGKASSAK.Q	
18	1	438.2870	64.99	2	16.3	26.8	1	243-249	R.LSQRFPK.A	
46	1	696.3030	27.25	3	18.6	56.7	0	265-281	K.VHTECCHGDLLECADDR.A	Carbamidomethyl: 5, 6, 13

63	2	862.4430	76.04	3	20.2	73.3	1	265-286	K.VHTECCHGDLLECADDRADLAK.Y	Carbamidomethyl: 5, 6, 13
39	1	722.3660	57.24	2	18.2	89.2	0	287-298	K.YICENQDSISSK.L	Carbamidomethyl: 3
27	1	773.9780	98.18	2	17.0	61.4	1	299-310	K.LKECCEKPLLEK.S	Carbamidomethyl: 4, 5
191	4	997.5310	79.89	3	31.4	105.8	0	311-337	K.SHCIAEVENDEMPADLPSLAADFVESK.D	Carbamidomethyl: 3; Oxidation: 12
184	2	997.8720	92.89	3	30.5	89.8	0	311-337	K.SHCIAEVENDEMPADLPSLAADFVESK.D	Carbamidomethyl: 3; Oxidation: 12; Deamidated: 9
202	2	992.5280	411.57	3	32.4	94.2	0	311-337	K.SHCIAEVENDEMPADLPSLAADFVESK.D	Carbamidomethyl: 3
10	1	599.3260	73.32	2	15.7	35.5	1	338-347	K.DVCKNYAEAK.D	Carbamidomethyl: 3
116	1	490.0320	162.18	3	24.6	73.1	1	361-372	R.RHPDYSVVLLLR.L	
179	1	682.4350	95.29	3	29.7	60.2	0	397-413	K.VFDEFKPLVEEPQNLIK.Q	
147	1	829.4650	102.53	2	27.0	93.9	0	414-426	K.QNCELFEQLGEYK.F	Carbamidomethyl: 3
186	1	867.1790	86.54	3	31.0	61.0	1	414-434	K.QNCELFEQLGEYKFQNALLVR.Y	Carbamidomethyl: 3
92	1	820.5280	67.63	2	22.7	63.1	1	438-452	K.KVPQVSTPTLVEVSR.N	
7	1	501.8200	74.62	2	15.0	35.1	1	491-499	K.TPVSDRVTK.C	
122	1	956.0700	105.15	2	25.2	86.0	0	509-524	R.RPCFSALEVDETYVPK.E	Carbamidomethyl: 3
142	1	849.1520	107.99	3	26.8	58.1	1	525-545	K.EFNAETFTFHADICTLSEKER.Q	Carbamidomethyl: 14
64	1	564.9200	118.65	2	20.2	71.3	1	549-558	K.KQTALVELVK.H	
12	1	814.4100	52.74	2	15.8	76.7	2	585-598	K.ADDKETCFAEEGKK.L	Carbamidomethyl: 7

Protein 3: Haptoglobin OS=Homo sapiens GN=HP PE=1 SV=1
Accession: HPT_HUMAN
Database: SwissProt_July2013
Seq. Coverage [%]: 2.70 %

Score: 65.76
MW [kDa]: 45.20
pI: 6.13
No. of Peptides: 1

Cmpd.	No. of Cmpds.	m/z meas.	Δ m/z [ppm]	z	Rt [min]	Score	P	Range	Sequence	Modification
3	1	637.9140	143.60	2	14.6	65.8	1	312-322	R.HYEGSTVPEKK.T	

Accumulated Protein Report

Search Location /Kathir/ABC 2016 IgG And A1AT/KA A1AT ISo Drop 1min E2_RC7_01_8276.d/

Protein 1: Alpha-1-antitrypsin OS=Homo sapiens GN=SERPINA1 PE=1 SV=3
Accession: A1AT_HUMAN **Score:** 2345.01
Database: SwissProt_July2013 **MW [kDa]:** 46.70
Seq. Coverage [%]: 61.20 % **pI:** 5.27
No. of Peptides: 33
Modification(s): Carbamidomethyl, Oxidation, Deamidated

Cmpd.	No. of Cmpds.	m/z meas.	Δ m/z [ppm]	z	Rt [min]	Score	P	Range	Sequence	Modification
3	2	890.4180	34.03	2	14.7	68.9	0	35-49	K.TDTSHHDQDHPTFNK.I	
214	1	821.5290	113.95	2	34.0	95.1	0	50-63	K.ITPNLAEFAFSLYR.Q	
200	1	1288.2000	26.29	2	32.1	150.2	0	126-149	R.TLNQPDSQLQLTTGNGLFLSEGL K.L	Deamidated: 15
190	2	1287.7120	29.41	2	31.2	121.5	0	126-149	R.TLNQPDSQLQLTTGNGLFLSEGL K.L	
69	1	667.4160	39.07	2	21.0	66.8	2	150-160	K.LVDKFLEDVKK.L	
72	3	772.1000	64.95	3	21.2	90.8	2	160-179	K.KLYHSEAFTVNFGDTEEAKK.Q	
115	1	1029.5720	93.08	2	24.8	122.5	0	161-178	K.LYHSEAFTVNFGDTEEAK.K	
94	1	1093.5890	59.75	2	22.9	71.5	1	161-179	K.LYHSEAFTVNFGDTEEAKK.Q	
22	1	568.8530	91.52	2	16.8	68.4	1	179-187	K.KQINDYVEK.G	
20	1	536.6700	97.33	3	16.7	60.4	2	179-192	K.KQINDYVEKGTQGK.I	
38	1	504.7970	86.25	2	18.2	40.9	0	180-187	K.QINDYVEK.G	
33	1	740.4080	43.85	2	17.8	45.3	1	180-192	K.QINDYVEKGTQGK.I	
43	1	638.3660	31.56	2	18.7	47.2	1	216-225	K.GKWERPFEVK.D	
108	1	1050.2530	70.55	3	24.0	89.3	2	216-241	K.GKWERPFEVKDTEEEDFHVDQVT TVK.V	
63	1	545.8590	130.74	2	20.5	32.8	0	218-225	K.WERPFEVK.D	
96	1	946.4990	71.39	2	23.2	110.9	0	226-241	K.DTEEEDFHVDQVTTVK.V	
106	1	837.4410	66.04	3	23.8	70.0	1	226-246	K.DTEEEDFHVDQVTTVKVPMMK.R	Oxidation: 19, 20
87	1	889.4940	83.88	3	22.3	52.4	2	226-247	K.DTEEEDFHVDQVTTVKVPMMKR. L	Oxidation: 19, 20
31	1	473.9150	22.55	3	17.7	34.3	1	247-257	K.RLGMFNIQHCK.K	Carbamidomethyl: 10; Oxidation: 4
27	1	464.6200	109.93	3	17.2	20.9	1	248-258	R.LGMFNIQHCKK.L	Carbamidomethyl: 9; Oxidation:

Accumulated Protein Report

										3
89	1	602.0430	85.55	3	22.6	55.9	0	284-298	K.LQHLENELTHDIITK.F	
18	1	539.8040	67.74	2	16.4	32.6	1	299-306	K.FLENEDRR.S	
30	1	426.7530	5.79	2	17.6	64.1	0	307-314	R.SASLHLPK.L	
109	1	555.8890	149.88	2	24.2	70.6	0	315-324	K.LSITGTYDLK.S	
119	1	508.3800	135.84	2	25.2	85.3	0	325-334	K.SVLGQLGITK.V	
155	1	918.0340	83.79	2	27.7	116.7	0	335-352	K.VFSNGADLSGVTEEAPLK.L	Deamidated: 4
134	2	917.5370	78.39	2	26.2	110.9	0	335-352	K.VFSNGADLSGVTEEAPLK.L	
135	3	721.4550	100.04	3	26.3	62.2	1	335-355	K.VFSNGADLSGVTEEAPLKLSK.A	
153	2	721.7880	106.92	3	27.5	59.5	1	335-355	K.VFSNGADLSGVTEEAPLKLSK.A	Deamidated: 4
202	1	1054.2920	77.73	3	32.4	51.5	1	360-389	K.AVLTIDEKGTEAAGAMFLEAIPM SIPPEVK.F	Oxidation: 16, 23
191	2	1146.1350	58.00	2	31.3	66.3	0	368-389	K.GTEAAGAMFLEAIPMSIPPEVK. F	Oxidation: 8, 15
152	2	624.7380	121.15	3	27.3	67.9	0	390-404	K.FNKPFVFLMIEQNTK.S	Oxidation: 9
151	1	620.0450	94.04	3	27.3	41.3	0	390-404	K.FNKPFVFLMIEQNTK.S	Deamidated: 12, 13

Protein 2: Serum albumin OS=Homo sapiens GN=ALB PE=1 SV=2

Accession: ALBU_HUMAN

Database: SwissProt_July2013

Seq. Coverage [%]: 67.00 %

Score: 2321.87

MW [kDa]: 69.30

pI: 5.88

No. of Peptides: 35

Modification(s): Carbamidomethyl, Oxidation, Deamidated

Cmpd.	No. of Cmpds.	m/z meas.	Δ m/z [ppm]	z	Rt [min]	Score	P	Range	Sequence	Modification
1	1	575.3690	134.52	2	14.4	63.2	1	25-34	R.DAHKSEVAHR.F	
51	1	613.8520	74.57	2	19.7	75.5	1	35-44	R.FKDLGEENFK.A	
14	1	749.8520	78.87	2	16.1	106.7	0	76-88	K.TCVADESAENCDK.S	Carbamidomethyl: 2, 11
138	1	1137.8950	21.30	3	26.5	71.3	2	76-105	K.TCVADESAENCDKSLHTLFGDKL CTVATLR.E	Carbamidomethyl: 2, 11, 24
19	1	725.8030	48.50	2	16.6	52.0	0	106-117	R.ETYGEMADCCAK.Q	Carbamidomethyl: 9, 10; Oxidation: 6
25	1	572.3080	65.74	3	17.1	39.0	1	118-130	K.QEPERNECFLQHK.D	Carbamidomethyl: 8
49	2	879.4790	74.07	3	19.5	34.9	2	118-138	K.QEPERNECFLQHKDDNPNLPR.L	Carbamidomethyl: 8
56	1	879.7920	56.99	3	20.0	44.9	2	118-138	K.QEPERNECFLQHKDDNPNLPR.L	Carbamidomethyl: 8; Deamidated: 6

50	1	666.3500	53.06	3	19.6	53.4	1	123-138	R.NECFLQHKDDNPNLPR.L	Carbamidomethyl: 3
35	1	470.7690	87.57	2	18.1	45.7	0	131-138	K.DDNPNLPR.L	
105	3	699.4000	80.18	4	23.7	47.6	1	139-161	R.LVRPEVDVMCTAFHDNEETFLKK .Y	Carbamidomethyl: 10; Oxidation: 9
85	1	464.3090	126.31	2	22.2	47.9	0	162-168	K.YLYEIAR.R	
176	1	633.7440	116.91	3	29.4	75.7	1	169-183	R.RHPYFYAPELLFFAK.R	
113	1	709.0650	90.72	3	24.7	70.5	1	187-205	K.AAFTECCQAADKAACLLPK.L	Carbamidomethyl: 6, 7, 15
12	1	759.9490	75.60	2	15.9	75.2	2	206-219	K.LDELRDEGKASSAK.Q	
17	1	438.2830	55.86	2	16.3	20.6	1	243-249	R.LSQRFPK.A	
42	1	696.3020	25.81	3	18.6	59.0	0	265-281	K.VHTECCHGDLLECADDR.A	Carbamidomethyl: 5, 6, 13
61	2	862.4310	62.12	3	20.3	65.6	1	265-286	K.VHTECCHGDLLECADDRADLAK. Y	Carbamidomethyl: 5, 6, 13
37	1	722.3630	53.08	2	18.2	74.0	0	287-298	K.YICENQDSISSK.L	Carbamidomethyl: 3
24	1	773.9740	93.01	2	17.0	58.2	1	299-310	K.LKECCEKPLLEK.S	Carbamidomethyl: 4, 5
192	5	997.5270	75.88	3	31.3	118.1	0	311-337	K.SHCIAEVENDEMPADLPSLAADF VESK.D	Carbamidomethyl: 3; Oxidation: 12
186	1	998.0970	318.39	3	30.9	88.1	0	311-337	K.SHCIAEVENDEMPADLPSLAADF VESK.D	Carbamidomethyl: 3; Oxidation: 12; Deamidated: 9
199	2	992.1990	79.95	3	32.0	108.1	0	311-337	K.SHCIAEVENDEMPADLPSLAADF VESK.D	Carbamidomethyl: 3
9	1	599.3310	81.66	2	15.8	33.0	1	338-347	K.DVCKNYAEAK.D	Carbamidomethyl: 3
112	1	490.0290	156.06	3	24.6	73.0	1	361-372	R.RHPDYSVVLLLR.L	
179	1	1023.1650	111.12	2	29.7	70.3	0	397-413	K.VFDEFKPLVEEPQNLIK.Q	
148	1	829.4680	106.15	2	27.1	97.1	0	414-426	K.QNCELFEQLGEYK.F	Carbamidomethyl: 3
188	1	867.1750	81.93	3	31.0	81.2	1	414-434	K.QNCELFEQLGEYKFQNALLVR.Y	Carbamidomethyl: 3
92	1	820.5290	68.85	2	22.8	97.2	1	438-452	K.KVPQVSTPTLVEVSR.N	
205	1	897.5380	107.55	3	32.7	43.5	1	469-490	K.RMPCAEDYLSVVLNQLCVLHEK. T	Carbamidomethyl: 4, 17; Oxidation: 2
5	1	501.8290	92.56	2	15.1	31.7	1	491-499	K.TPVSDRVTK.C	
120	1	956.0640	98.88	2	25.3	64.5	0	509-524	R.RPCFSALEVDETYVPK.E	Carbamidomethyl: 3
146	1	849.1600	117.41	3	26.9	70.8	1	525-545	K.EFNAETFTFHADICTLSEKER.Q	Carbamidomethyl: 14
62	1	564.9230	123.96	2	20.3	81.6	1	549-558	K.KQTALVELVK.H	
11	1	814.4090	51.51	2	15.9	64.1	2	585-598	K.ADDKETCFAEEGKK.L	Carbamidomethyl: 7

**Protein 3:**	Haptoglobin OS=Homo sapiens GN=HP PE=1 SV=1		
**Accession:**	HPT_HUMAN	**Score:**	64.33
**Database:**	SwissProt_July2013	**MW [kDa]:**	45.20

Seq. Coverage [%]: 2.70 %

pI: 6.13
No. of Peptides: 1

Cmpd.	No. of Cmpds.	m/z meas.	Δ m/z [ppm]	z	Rt [min]	Score	P	Range	Sequence	Modification
2	1	637.9140	143.60	2	14.6	64.3	1	312-322	R.HYEGSTVPEKK.T	

Search Type Combined MS/MS - ProteinExtractor
Search Result Amazon Human_SPJuly2013_2016-08-08 18:23:29
Search Location /Kathir/ABC 2016 IgG And A1AT/KA A1AT ISo Drop 1min E3_RC8_01_8277.d/

Protein 1: Serum albumin OS=Homo sapiens GN=ALB PE=1 SV=2
Accession: ALBU_HUMAN
Database: SwissProt_July2013
Seq. Coverage [%]: 74.50 %

Score: 2613.53
MW [kDa]: 69.30
pI: 5.88
No. of Peptides: 39

Modification(s): Carbamidomethyl, Oxidation, Deamidated

Cmpd.	No. of Cmpds.	m/z meas.	Δ m/z [ppm]	z	Rt [min]	Score	P	Range	Sequence	Modification
1	1	575.3780	150.17	2	14.4	58.9	1	25-34	R.DAHKSEVAHR.F	
54	1	613.8600	87.60	2	19.7	72.2	1	35-44	R.FKDLGEENFK.A	
210	1	830.8480	98.09	3	34.7	90.5	0	45-65	K.ALVLIAFAQYLQQCPFEDHVK.L	Carbamidomethyl: 14
89	1	575.3580	81.44	2	22.9	66.6	0	66-75	K.LVNEVTEFAK.T	
16	1	749.8500	76.20	2	16.1	111.9	0	76-88	K.TCVADESAENCDK.S	Carbamidomethyl: 2, 11
136	2	1138.2380	34.46	3	26.5	70.9	2	76-105	K.TCVADESAENCDKSLHTLFGDKL CTVATLR.E	Carbamidomethyl: 2, 11, 24; Deamidated: 10
124	1	644.7590	116.49	3	25.8	62.7	1	89-105	K.SLHTLFGDKLCTVATLR.E	Carbamidomethyl: 11
21	1	725.7980	41.61	2	16.6	65.9	0	106-117	R.ETYGEMADCCAK.Q	Carbamidomethyl: 9, 10; Oxidation: 6
29	1	572.3040	58.75	3	17.0	38.1	1	118-130	K.QEPERNECFLQHK.D	Carbamidomethyl: 8
51	2	879.7600	20.62	3	19.2	37.4	2	118-138	K.QEPERNECFLQHKDDNPNLPR.L	Carbamidomethyl: 8; Deamidated: 6

52	2	879.4950	92.27	3	19.4	33.0	2	118-138	K.QEPERNECFLQHKDDNPNLPR.L	Carbamidomethyl: 8
40	1	470.7610	70.57	2	18.1	38.2	0	131-138	K.DDNPNLPR.L	
126	1	695.4100	93.20	4	25.9	63.6	1	139-161	R.LVRPEVDVMCTAFHDNEETFLKK .Y	Carbamidomethyl: 10
115	2	699.6550	444.81	4	24.9	55.7	1	139-161	R.LVRPEVDVMCTAFHDNEETFLKK .Y	Carbamidomethyl: 10; Oxidation: 9
79	1	464.3180	145.70	2	22.2	46.0	0	162-168	K.YLYEIAR.R	
170	1	633.7470	121.64	3	29.4	67.5	1	169-183	R.RHPYFYAPELLFFAK.R	
113	1	709.0600	83.66	3	24.7	67.9	1	187-205	K.AAFTECCQAADKAACLLPK.L	Carbamidomethyl: 6, 7, 15
15	1	506.9710	80.63	3	15.9	67.5	2	206-219	K.LDELRDEGKASSAK.Q	
19	1	438.2860	62.70	2	16.4	24.6	1	243-249	R.LSQRFPK.A	
38	1	440.7590	79.02	2	17.8	51.1	0	250-257	K.AEFAEVSK.L	
96	1	839.7130	77.01	4	23.5	33.8	2	258-286	K.LVTDLTKVHTECCHGDLLECADD RADLAK.Y	Carbamidomethyl: 12, 13, 20
46	1	696.3030	27.25	3	18.6	67.9	0	265-281	K.VHTECCHGDLLECADDR.A	Carbamidomethyl: 5, 6, 13
67	2	862.4410	73.72	3	20.9	53.1	1	265-286	K.VHTECCHGDLLECADDRADLAK. Y	Carbamidomethyl: 5, 6, 13
41	1	722.3560	43.39	2	18.2	90.8	0	287-298	K.YICENQDSISSK.L	Carbamidomethyl: 3
28	1	773.9790	99.47	2	17.0	59.3	1	299-310	K.LKECCEKPLLEK.S	Carbamidomethyl: 4, 5
188	3	997.5220	70.86	3	31.4	116.7	0	311-337	K.SHCIAEVENDEMPADLPSLAADF VESK.D	Carbamidomethyl: 3; Oxidation: 12
194	1	992.1910	71.89	3	32.1	117.8	0	311-337	K.SHCIAEVENDEMPADLPSLAADF VESK.D	Carbamidomethyl: 3
11	1	599.3310	81.66	2	15.7	32.7	1	338-347	K.DVCKNYAEAK.D	Carbamidomethyl: 3
111	1	490.0230	143.82	3	24.6	70.2	1	361-372	R.RHPDYSVVLLLR.L	
174	1	1023.1590	105.25	2	29.7	76.3	0	397-413	K.VFDEFKPLVEEPQNLIK.Q	
184	1	867.1840	92.30	3	31.0	97.6	1	414-434	K.QNCELFEQLGEYKFQNALLVR.Y	Carbamidomethyl: 3
88	1	820.5230	61.54	2	22.8	69.5	1	438-452	K.KVPQVSTPTLVEVSR.N	
199	1	897.5390	108.66	3	32.6	62.9	1	469-490	K.RMPCAEDYLSVVLNQLCVLHEK. T	Carbamidomethyl: 4, 17; Oxidation: 2
203	1	798.2130	91.60	4	33.2	76.8	1	470-496	R.MPCAEDYLSVVLNQLCVLHEKTP VSDR.V	Carbamidomethyl: 3, 16; Oxidation: 1
5	1	501.8210	76.62	2	15.0	31.8	1	491-499	K.TPVSDRVTK.C	
117	1	637.7080	92.93	3	25.3	87.3	0	509-524	R.RPCFSALEVDETYVPK.E	Carbamidomethyl: 3
139	1	849.1510	106.81	3	26.8	91.5	1	525-545	K.EFNAETFTFHADICTLSEKER.Q	Carbamidomethyl: 14
61	1	564.9160	111.57	2	20.2	81.8	1	549-558	K.KQTALVELVK.H	
12	1	543.2770	54.99	3	15.8	70.3	2	585-598	K.ADDKETCFAEEGKK.L	Carbamidomethyl: 7

Accumulated Protein Report

Protein 2: Alpha-1-antitrypsin OS=Homo sapiens GN=SERPINA1 PE=1 SV=3
Accession: A1AT_HUMAN
Database: SwissProt_July2013
Seq. Coverage [%]: 59.30 %
Score: 2214.96
MW [kDa]: 46.70
pI: 5.27
No. of Peptides: 32
Modification(s): Carbamidomethyl, Oxidation, Deamidated

Cmpd.	No. of Cmpds.	m/z meas.	Δ m/z [ppm]	z	Rt [min]	Score	P	Range	Sequence	Modification
3	2	593.9520	41.16	3	14.8	55.1	0	35-49	K.TDTSHHDQDHPTFNK.I	
171	1	1134.9710	83.61	3	29.5	60.0	1	35-63	K.TDTSHHDQDHPTFNKITPNLAEF AFSLYR.Q	
208	1	821.5310	116.38	2	34.0	91.9	0	50-63	K.ITPNLAEFAFSLYR.Q	
195	1	1288.2060	30.95	2	32.1	124.2	0	126-149	R.TLNQPDSQLQLTTGNGLFLSEGL K.L	Deamidated: 15
186	2	1287.7280	41.84	2	31.2	129.5	0	126-149	R.TLNQPDSQLQLTTGNGLFLSEGL K.L	
69	1	667.3980	12.10	2	21.0	68.2	2	150-160	K.LVDKFLEDVKK.L	
73	1	772.1040	70.14	3	21.3	87.6	2	160-179	K.KLYHSEAFTVNFGDTEEAKK.Q	
112	1	686.7030	72.51	3	24.6	72.0	0	161-178	K.LYHSEAFTVNFGDTEEAK.K	
90	2	1093.6050	74.39	2	23.0	68.5	1	161-179	K.LYHSEAFTVNFGDTEEAKK.Q	
26	1	568.8520	89.76	2	16.8	70.6	1	179-187	K.KQINDYVEK.G	
23	1	804.4920	85.75	2	16.7	72.8	2	179-192	K.KQINDYVEKGTQGK.I	
43	1	504.7990	90.21	2	18.3	55.4	0	180-187	K.QINDYVEK.G	
36	1	740.4060	41.14	2	17.8	56.5	1	180-192	K.QINDYVEKGTQGK.I	
47	1	638.3700	37.82	2	18.7	37.5	1	216-225	K.GKWERPFEVK.D	
99	2	1050.2550	72.45	3	23.7	100.2	2	216-241	K.GKWERPFEVKDTEEEDFHVDQVT TVK.V	
62	1	545.8530	119.75	2	20.4	34.2	0	218-225	K.WERPFEVK.D	
92	1	946.4960	68.22	2	23.2	73.8	0	226-241	K.DTEEEDFHVDQVTTVK.V	
106	2	837.4410	66.04	3	24.2	88.9	1	226-246	K.DTEEEDFHVDQVTTVKVPMMK.R	Oxidation: 19, 20
82	1	889.4970	87.25	3	22.3	26.2	2	226-247	K.DTEEEDFHVDQVTTVKVPMMKR. L	Oxidation: 19, 20
35	1	473.9200	33.10	3	17.6	20.5	1	247-257	K.RLGMFNIQHCK.K	Carbamidomethyl: 10; Oxidation: 4
31	1	464.6150	99.17	3	17.1	25.9	1	248-258	R.LGMFNIQHCKK.L	Carbamidomethyl: 9; Oxidation: 3

84	1	602.0400	80.57	3	22.6	52.3	0	284-298	K.LQHLENELTHDIITK.F	
20	1	539.8030	65.89	2	16.4	26.1	1	299-306	K.FLENEDRR.S	
34	1	426.7540	8.14	2	17.6	42.5	0	307-314	R.SASLHLPK.L	
107	1	555.8750	124.69	2	24.2	87.5	0	315-324	K.LSITGTYDLK.S	
116	1	508.3790	133.88	2	25.1	81.7	0	325-334	K.SVLGQLGITK.V	
152	1	918.0310	80.52	2	27.7	120.1	0	335-352	K.VFSNGADLSGVTEEAPLK.L	Deamidated: 4
132	2	917.5340	75.12	2	26.2	104.4	0	335-352	K.VFSNGADLSGVTEEAPLK.L	
133	4	721.4570	102.82	3	26.3	66.9	1	335-355	K.VFSNGADLSGVTEEAPLKLSK.A	
149	1	1082.1740	102.94	2	27.5	64.4	1	335-355	K.VFSNGADLSGVTEEAPLKLSK.A	Deamidated: 4
187	2	1146.1340	57.13	2	31.3	57.5	0	368-389	K.GTEAAGAMFLEAIPMSIPPEVK.F	Oxidation: 8, 15
145	1	936.5870	103.74	2	27.3	74.0	0	390-404	K.FNKPFVFLMIEQNTK.S	Oxidation: 9

Protein 3: Haptoglobin OS=Homo sapiens GN=HP PE=1 SV=1

**Accession:**	HPT_HUMAN	**Score:**	57.36
**Database:**	SwissProt_July2013	**MW [kDa]:**	45.20
**Seq. Coverage [%]:**	2.70 %	**pI:**	6.13
		**No. of Peptides:**	1

Cmpd.	No. of Cmpds.	m/z meas.	Δ m/z [ppm]	z	Rt [min]	Score	P	Range	Sequence	Modification
2	1	637.9060	131.06	2	14.6	57.4	1	312-322	R.HYEGSTVPEKK.T	

Search Type Combined MS/MS - ProteinExtractor
Search Result Amazon Human_SPJuly2013_2016-08-08 18:23:29
Search Location /Kathir/ABC 2016 IgG And A1AT/KA IgG EtOH Drop 1min E1_RD1_01_8278.d/

Protein 1: Ig kappa chain C region OS=Homo sapiens GN=IGKC PE=1 SV=1

**Accession:**	IGKC_HUMAN	**Score:**	854.69
**Database:**	SwissProt_July2013	**MW [kDa]:**	11.60
**Seq. Coverage [%]:**	75.50 %	**pI:**	5.53
		**No. of Peptides:**	9

Modification(s): Carbamidomethyl, Deamidated

Cmpd.	No. of Cmpds.	m/z meas.	Δ m/z [ppm]	z	Rt [min]	Score	P	Range	Sequence	Modification
185	2	973.6210	106.71	2	30.9	65.0	0	1-18	-.TVAAPSVFIFPPSDEQLK.S	
116	1	893.1230	31.52	3	22.5	111.2	1	38-61	K.VQWKVDNALQSGNSQESVTEQDSK.D	
73	4	1068.5390	47.73	2	18.7	120.8	0	42-61	K.VDNALQSGNSQESVTEQDSK.D	
85	1	1069.0510	66.41	2	19.6	146.9	0	42-61	K.VDNALQSGNSQESVTEQDSK.D	Deamidated: 9
141	1	1207.3000	48.63	3	26.0	92.8	1	42-75	K.VDNALQSGNSQESVTEQDSKDSTYSLSSTLTLSK.A	
150	1	752.0320	198.35	2	27.1	113.4	0	62-75	K.DSTYSLSSTLTLSK.A	
27	1	445.7750	118.98	2	14.4	25.1	1	76-82	K.ADYEKHK.V	
74	1	714.4350	97.82	3	18.7	106.0	1	81-99	K.HKVYACEVTHQGLSSPVTK.S	Carbamidomethyl: 6
93	1	626.0410	96.66	3	20.4	73.5	0	83-99	K.VYACEVTHQGLSSPVTK.S	Carbamidomethyl: 4

Protein 2: Ig gamma-1 chain C region OS=Homo sapiens GN=IGHG1 PE=1 SV=1
Accession: IGHG1_HUMAN
Database: SwissProt_July2013
Seq. Coverage [%]: 47.90 %
Score: 831.19
MW [kDa]: 36.10
pI: 9.36
No. of Peptides: 13

Modification(s): Carbamidomethyl, Deamidated

Cmpd.	No. of Cmpds.	m/z meas.	Δ m/z [ppm]	z	Rt [min]	Score	P	Range	Sequence	Modification
134	1	593.9190	154.99	2	24.6	69.2	0	5-16	K.GPSVFPLAPSSK.S	
118	1	661.4120	104.86	2	22.6	65.5	0	17-30	K.STSGGTAALGCLVK.D	Carbamidomethyl: 11
162	1	1112.3040	76.52	3	27.9	67.6	1	102-131	K.SCDKTHTCPPCPAPELLGGPSVFLFPPKPK.D	Carbamidomethyl: 2, 8, 11
137	1	839.4890	100.51	2	24.9	94.8	0	158-171	K.FNWYVDGVEVHNAK.T	
117	2	643.9210	124.87	2	22.5	26.9	0	228-238	R.EPQVYTLPPSR.D	
125	1	937.0510	66.47	2	23.1	29.6	1	228-243	R.EPQVYTLPPSRDELTK.N	
130	1	581.3860	116.20	2	23.9	71.4	0	244-253	K.NQVSLTCLVK.G	Carbamidomethyl: 7
181	2	1272.6210	40.61	2	29.8	100.2	0	254-275	K.GFYPSDIAVEWESNGQPENNYK.T	
182	4	1273.1190	45.30	2	30.2	122.2	0	254-275	K.GFYPSDIAVEWESNGQPENNYK.T	Deamidated: 14

186	3	1273.6000	36.64	2	30.9	78.0	0	254-275	K.GFYPSDIAVEWESNGQPENNYK.T	Deamidated: 14, 20
184	3	1273.5980	35.07	2	30.7	93.7	0	254-275	K.GFYPSDIAVEWESNGQPENNYK.T	Deamidated: 14, 19
183	1	937.5570	98.62	2	30.5	67.4	0	276-292	K.TTPPVLDSDGSFFLYSK.L	
128	1	934.3760	-54.82	3	23.7	22.7	0	300-322	R.WQQGNVFSCSVMHEALHNHYTQK.S	Carbamidomethyl: 9

Protein 3: Ig gamma-4 chain C region OS=Homo sapiens GN=IGHG4 PE=1 SV=1
Accession: IGHG4_HUMAN **Score:** 500.63
Database: SwissProt_July2013 **MW [kDa]:** 35.90
Seq. Coverage [%]: 8.60 % **pI:** 7.80
No. of Peptides: 2

Modification(s): Carbamidomethyl, Deamidated

Cmpd.	No. of Cmpds.	m/z meas.	Δ m/z [ppm]	z	Rt [min]	Score	P	Range	Sequence	Modification
126	1	712.4230	90.55	2	23.3	72.4	0	17-30	R.STSESTAALGCLVK.D	Carbamidomethyl: 11
31	1	555.6240	52.65	3	14.8	40.7	0	80-93	K.TYTCNVDHKPSNTK.V	Carbamidomethyl: 4

Protein 4: Ig lambda-3 chain C regions OS=Homo sapiens GN=IGLC3 PE=1 SV=1
Accession: LAC3_HUMAN **Score:** 312.02
Database: SwissProt_July2013 **MW [kDa]:** 11.20
Seq. Coverage [%]: 55.70 % **pI:** 7.72
No. of Peptides: 4

Modification(s): Carbamidomethyl, Deamidated

Cmpd.	No. of Cmpds.	m/z meas.	Δ m/z [ppm]	z	Rt [min]	Score	P	Range	Sequence	Modification
152	1	993.6050	93.04	2	27.3	89.1	0	5-23	K.AAPSVTLFPPSSEELQANK.A	
37	1	495.8210	125.57	2	15.4	61.3	0	51-60	K.AGVETTTPSK.Q	
166	2	873.0470	703.72	2	28.5	82.3	0	66-80	K.YAASSYLSLTPEQWK.S	
53	1	856.4630	93.14	2	16.8	79.4	0	84-98	K.SYSCQVTHEGSTVEK.T	Carbamidomethyl: 4

Protein 5: Ig gamma-2 chain C region OS=Homo sapiens GN=IGHG2 PE=1 SV=2
Accession: IGHG2_HUMAN **Score:** 273.87
Database: SwissProt_July2013 **MW [kDa]:** 35.90

Seq. Coverage [%]: 12.90 %
pI: 8.80
No. of Peptides: 2
Modification(s): Carbamidomethyl, Oxidation

Cmpd.	No. of Cmpds.	m/z meas.	Δ m/z [ppm]	z	Rt [min]	Score	P	Range	Sequence	Modification
177	1	970.2110	74.48	3	29.2	48.3	0	102-127	K.CCVECPPCPAPPVAGPSVFLFPP KPK.D	Carbamidomethyl: 1, 2, 5, 8
95	2	961.0620	93.43	2	20.7	32.1	1	224-239	R.EPQVYTLPPSREEMTK.N	Oxidation: 14

Protein 6: Ig heavy chain V-III region WEA OS=Homo sapiens PE=1 SV=1
Accession: HV302_HUMAN
Score: 71.50
Database: SwissProt_July2013
MW [kDa]: 12.20
Seq. Coverage [%]: 9.60 %
pI: 9.50
No. of Peptides: 1
Modification(s): Carbamidomethyl

Cmpd.	No. of Cmpds.	m/z meas.	Δ m/z [ppm]	z	Rt [min]	Score	P	Range	Sequence	Modification
89	1	660.3630	868.08	2	20.2	71.5	0	88-98	R.AEDTAVYYCAR.G	Carbamidomethyl: 9

Search Type Combined MS/MS - ProteinExtractor
Search Result Amazon Human_SPJuly2013_2016-08-08 18:23:29
Search Location /Kathir/ABC 2016 IgG And A1AT/KA IgG EtOH Drop 1min E2_RD2_01_8279.d/

Protein 1: Ig gamma-1 chain C region OS=Homo sapiens GN=IGHG1 PE=1 SV=1
Accession: IGHG1_HUMAN
Score: 824.62
Database: SwissProt_July2013
MW [kDa]: 36.10
Seq. Coverage [%]: 46.70 %
pI: 9.36
No. of Peptides: 12
Modification(s): Carbamidomethyl, Deamidated

Cmpd.	No. of Cmpds.	m/z meas.	Δ m/z [ppm]	z	Rt [min]	Score	P	Range	Sequence	Modification

146	1	593.9270	168.46	2	24.6	57.8	0	5-16	K.GPSVFPLAPSSK.S	
135	1	661.4080	98.81	2	22.6	90.5	0	17-30	K.STSGGTAALGCLVK.D	Carbamidomethyl: 11
187	2	949.2480	446.84	3	28.7	62.4	0	106-131	K.THTCPPCPAPELLGGPSVFLFPPKPK.D	Carbamidomethyl: 4, 7
149	1	560.6630	707.07	3	24.9	38.5	0	158-171	K.FNWYVDGVEVHNAK.T	Deamidated: 2
150	1	839.5240	142.21	2	24.9	95.4	0	158-171	K.FNWYVDGVEVHNAK.T	
133	1	643.8980	89.15	2	22.2	41.5	0	228-238	R.EPQVYTLPPSR.D	
137	1	625.0600	104.15	3	23.1	24.8	1	228-243	R.EPQVYTLPPSRDELTK.N	
142	1	581.3830	111.04	2	23.8	73.9	0	244-253	K.NQVSLTCLVK.G	Carbamidomethyl: 7
192	2	1272.6190	39.04	2	29.8	92.7	0	254-275	K.GFYPSDIAVEWESNGQPENNYK.T	
193	1	1273.1200	46.08	2	30.2	109.6	0	254-275	K.GFYPSDIAVEWESNGQPENNYK.T	Deamidated: 14
194	1	937.5690	111.42	2	30.5	98.3	0	276-292	K.TTPPVLDSDGSFFLYSK.L	
141	1	934.4580	32.94	3	23.7	39.1	0	300-322	R.WQQGNVFSCSVMHEALHNHYTQK.S	Carbamidomethyl: 9

Protein 2: Ig kappa chain C region OS=Homo sapiens GN=IGKC PE=1 SV=1

Accession: IGKC_HUMAN

Database: SwissProt_July2013

Seq. Coverage [%]: 58.50 %

Score: 691.98

MW [kDa]: 11.60

pI: 5.53

No. of Peptides: 8

Modification(s): Carbamidomethyl, Deamidated

Cmpd.	No. of Cmpds.	m/z meas.	Δ m/z [ppm]	z	Rt [min]	Score	P	Range	Sequence	Modification
136	2	893.1120	19.20	3	22.9	106.7	1	38-61	K.VQWKVDNALQSGNSQESVTEQDSK.D	
104	2	1068.9680	-11.23	2	19.1	84.5	0	42-61	K.VDNALQSGNSQESVTEQDSK.D	Deamidated: 3
103	6	1068.8500	338.80	2	19.0	145.5	0	42-61	K.VDNALQSGNSQESVTEQDSK.D	
159	2	1207.2930	42.83	3	26.0	135.2	1	42-75	K.VDNALQSGNSQESVTEQDSKDSTYSLSSTLTLSK.A	
163	2	1207.9470	312.78	3	26.5	114.8	1	42-75	K.VDNALQSGNSQESVTEQDSKDSTYSLSSTLTLSK.A	Deamidated: 17
54	1	445.7870	145.90	2	14.4	28.7	1	76-82	K.ADYEKHK.V	
101	1	714.4240	82.43	3	18.6	80.6	1	81-99	K.HKVYACEVTHQGLSSPVTK.S	Carbamidomethyl: 6
118	1	938.5610	100.06	2	20.4	80.6	0	83-99	K.VYACEVTHQGLSSPVTK.S	Carbamidomethyl: 4

Protein 3: Ig gamma-4 chain C region OS=Homo sapiens GN=IGHG4 PE=1 SV=1
Accession: IGHG4_HUMAN
Database: SwissProt_July2013
Seq. Coverage [%]: 8.60 %
Score: 387.44
MW [kDa]: 35.90
pI: 7.80
No. of Peptides: 2
Modification(s): Carbamidomethyl, Deamidated

Cmpd.	No. of Cmpds.	m/z meas.	Δ m/z [ppm]	z	Rt [min]	Score	P	Range	Sequence	Modification
139	1	712.4290	98.97	2	23.3	78.8	0	17-30	R.STSESTAALGCLVK.D	Carbamidomethyl: 11
59	1	555.6460	92.24	3	14.7	32.4	0	80-93	K.TYTCNVDHKPSNTK.V	Carbamidomethyl: 4

Protein 4: Ig lambda-3 chain C regions OS=Homo sapiens GN=IGLC3 PE=1 SV=1
Accession: LAC3_HUMAN
Database: SwissProt_July2013
Seq. Coverage [%]: 41.50 %
Score: 223.82
MW [kDa]: 11.20
pI: 7.72
No. of Peptides: 3
Modification(s): Carbamidomethyl

Cmpd.	No. of Cmpds.	m/z meas.	Δ m/z [ppm]	z	Rt [min]	Score	P	Range	Sequence	Modification
173	2	993.5990	87.00	2	27.4	66.9	0	5-23	K.AAPSVTLFPPSSEELQANK.A	
67	1	495.8160	115.49	2	15.4	66.9	0	51-60	K.AGVETTTPSK.Q	
82	1	856.4440	70.96	2	16.8	90.1	0	84-98	K.SYSCQVTHEGSTVEK.T	Carbamidomethyl: 4

Search Type Combined MS/MS - ProteinExtractor
Search Result Amazon Human_SPJuly2013_2016-08-08 18:23:29
Search Location /Kathir/ABC 2016 IgG And A1AT/KA IgG EtOH Drop 1min E3_RD3_01_8280.d/

Protein 1: Ig gamma-1 chain C region OS=Homo sapiens GN=IGHG1 PE=1 SV=1
Accession: IGHG1_HUMAN
Database: SwissProt_July2013
Score: 1006.67
MW [kDa]: 36.10

Seq. Coverage [%]: 53.60 %

pI: 9.36

No. of Peptides: 13

Modification(s): Carbamidomethyl, Deamidated

Cmpd.	No. of Cmpds.	m/z meas.	Δ m/z [ppm]	z	Rt [min]	Score	P	Range	Sequence	Modification
137	1	593.9230	161.72	2	24.6	73.2	0	5-16	K.GPSVFPLAPSSK.S	
122	1	661.4180	113.93	2	22.6	93.1	0	17-30	K.STSGGTAALGCLVK.D	Carbamidomethyl: 11
169	2	1112.3020	74.72	3	28.0	54.7	1	102-131	K.SCDKTHTCPPCPAPELLGGPSVF LFPPKPK.D	Carbamidomethyl: 2, 8, 11
141	1	1070.0440	24.90	2	25.0	34.2	0	139-157	R.TPEVTCVVVDVSHEDPEVK.F	Carbamidomethyl: 6
139	1	839.5540	177.95	2	24.8	94.8	0	158-171	K.FNWYVDGVEVHNAK.T	
119	1	643.8980	89.15	2	22.3	42.7	0	228-238	R.EPQVYTLPPSR.D	
124	1	937.0670	83.54	2	22.8	29.6	1	228-243	R.EPQVYTLPPSRDELTK.N	
131	1	581.3980	136.85	2	23.7	73.8	0	244-253	K.NQVSLTCLVK.G	Carbamidomethyl: 7
189	3	1272.6190	39.04	2	29.8	97.8	0	254-275	K.GFYPSDIAVEWESNGQPENNYK. T	
190	3	1273.1200	46.08	2	30.2	108.7	0	254-275	K.GFYPSDIAVEWESNGQPENNYK. T	Deamidated: 14
192	1	1273.6100	44.49	2	30.6	100.1	0	254-275	K.GFYPSDIAVEWESNGQPENNYK. T	Deamidated: 14, 19
191	1	937.5670	109.28	2	30.4	95.8	0	276-292	K.TTPPVLDSDGSFFLYSK.L	
130	1	934.8400	441.74	3	23.7	43.5	0	300-322	R.WQQGNVFSCSVMHEALHNHYTQK .S	Carbamidomethyl: 9

Protein 2: Ig kappa chain C region OS=Homo sapiens GN=IGKC PE=1 SV=1

Accession: IGKC_HUMAN

Score: 943.25

Database: SwissProt_July2013

MW [kDa]: 11.60

Seq. Coverage [%]: 97.20 %

pI: 5.53

No. of Peptides: 11

Modification(s): Carbamidomethyl, Deamidated

Cmpd.	No. of Cmpds.	m/z meas.	Δ m/z [ppm]	z	Rt [min]	Score	P	Range	Sequence	Modification
194	2	973.6220	107.74	2	31.0	63.4	0	1-18	-.TVAAPSVFIFPPSDEQLK.S	
197	1	899.5420	100.89	2	32.2	64.0	0	19-34	K.SGTASVVCLLNNFYPR.E	Carbamidomethyl: 8
121	2	893.1360	46.08	3	22.4	103.4	1	38-61	K.VQWKVDNALQSGNSQESVTEQDS	

									K.D	
87	4	1068.9490	431.45	2	19.1	127.8	0	42-61	K.VDNALQSGNSQESVTEQDSK.D	
94	2	1069.0500	65.47	2	19.6	123.3	0	42-61	K.VDNALQSGNSQESVTEQDSK.D	Deamidated: 9
150	4	1207.6030	299.61	3	26.1	126.2	1	42-75	K.VDNALQSGNSQESVTEQDSKDST YSLSSTLTLSK.A	
155	1	751.9740	121.21	2	27.0	93.4	0	62-75	K.DSTYSLSSTLTLSK.A	
37	1	445.8000	175.07	2	14.4	31.5	1	76-82	K.ADYEKHK.V	
83	1	714.4390	103.42	3	18.5	92.4	1	81-99	K.HKVYACEVTHQGLSSPVTK.S	Carbamidomethyl: 6
99	1	938.5610	100.06	2	20.4	73.9	0	83-99	K.VYACEVTHQGLSSPVTK.S	Carbamidomethyl: 4
43	1	435.2270	103.06	2	15.1	43.9	1	100-106	K.SFNRGEC.-	Carbamidomethyl: 7

Protein 3: Ig gamma-4 chain C region OS=Homo sapiens GN=IGHG4 PE=1 SV=1
Accession: IGHG4_HUMAN **Score:** 612.56
Database: SwissProt_July2013 **MW [kDa]:** 35.90
Seq. Coverage [%]: 12.20 % **pI:** 7.80
No. of Peptides: 3
Modification(s): Carbamidomethyl, Deamidated

Cmpd.	No. of Cmpds.	m/z meas.	Δ m/z [ppm]	z	Rt [min]	Score	P	Range	Sequence	Modification
146	2	644.4200	140.69	2	25.5	52.1	0	5-16	K.GPSVFPLAPCSR.S	Carbamidomethyl: 10
127	1	712.4260	94.76	2	23.3	70.8	0	17-30	R.STSESTAALGCLVK.D	Carbamidomethyl: 11
40	1	555.6400	81.45	3	14.7	25.4	0	80-93	K.TYTCNVDHKPSNTK.V	Carbamidomethyl: 4

Protein 4: Ig gamma-2 chain C region OS=Homo sapiens GN=IGHG2 PE=1 SV=2
Accession: IGHG2_HUMAN **Score:** 418.39
Database: SwissProt_July2013 **MW [kDa]:** 35.90
Seq. Coverage [%]: 10.10 % **pI:** 8.80
No. of Peptides: 3
Modification(s): Carbamidomethyl, Oxidation, Deamidated

Cmpd.	No. of Cmpds.	m/z meas.	Δ m/z [ppm]	z	Rt [min]	Score	P	Range	Sequence	Modification
101	2	961.0630	94.47	2	20.6	54.0	1	224-239	R.EPQVYTLPPSREEMTK.N	Oxidation: 14
115	1	635.7060	84.54	3	21.8	23.3	1	224-239	R.EPQVYTLPPSREEMTK.N	
183	2	961.5370	92.52	2	29.2	58.3	0	272-288	K.TTPPMLDSDGSFFLYSK.L	Oxidation: 5

Accumulated Protein Report

Protein 5: Ig lambda-3 chain C regions OS=Homo sapiens GN=IGLC3 PE=1 SV=1
Accession: LAC3_HUMAN
Database: SwissProt_July2013
Seq. Coverage [%]: 63.20 %
Modification(s): Carbamidomethyl, Deamidated

Score: 356.83
MW [kDa]: 11.20
pI: 7.72
No. of Peptides: 5

Cmpd.	No. of Cmpds.	m/z meas.	Δ m/z [ppm]	z	Rt [min]	Score	P	Range	Sequence	Modification
158	3	994.1190	610.40	2	27.3	85.2	0	5-23	K.AAPSVTLFPPSSEELQANK.A	
49	1	495.8130	109.44	2	15.5	70.7	0	51-60	K.AGVETTTPSK.Q	
175	2	873.0570	715.18	2	28.4	90.2	0	66-80	K.YAASSYLSLTPEQWK.S	
63	2	856.4570	86.14	2	16.8	69.3	0	84-98	K.SYSCQVTHEGSTVEK.T	Carbamidomethyl: 4
102	1	853.0760	37.25	3	20.6	41.4	1	84-106	K.SYSCQVTHEGSTVEKTVAPTECS .-	Carbamidomethyl: 4, 22

Search Type Combined MS/MS - ProteinExtractor
Search Result Amazon Human_SPJuly2013_2016-08-08 18:23:29
Search Location /Kathir/ABC 2016 IgG And A1AT/KA IgG ISo Drop 1min E1_RC3_01_8272.d/

Protein 1: Ig gamma-1 chain C region OS=Homo sapiens GN=IGHG1 PE=1 SV=1
Accession: IGHG1_HUMAN
Database: SwissProt_July2013
Seq. Coverage [%]: 59.40 %
Modification(s): Carbamidomethyl, Oxidation, Deamidated

Score: 1174.82
MW [kDa]: 36.10
pI: 9.36
No. of Peptides: 17

Cmpd.	No. of Cmpds.	m/z meas.	Δ m/z [ppm]	z	Rt [min]	Score	P	Range	Sequence	Modification
109	1	593.8890	104.47	2	24.6	71.7	0	5-16	K.GPSVFPLAPSSK.S	
85	1	661.4130	106.37	2	22.5	99.8	0	17-30	K.STSGGTAALGCLVK.D	Carbamidomethyl: 11
139	2	1112.2360	15.38	3	27.8	66.1	1	102-131	K.SCDKTHTCPPCPAPELLGGPSVF LFPPKPK.D	Carbamidomethyl: 2, 8, 11
149	1	949.2000	396.25	3	28.7	75.6	0	106-131	K.THTCPPCPAPELLGGPSVFLFPP	Carbamidomethyl: 4, 7

									KPK.D	
112	1	1070.1190	95.00	2	25.1	83.4	0	139-157	R.TPEVTCVVVDVSHEDPEVK.F	Carbamidomethyl: 6
111	1	839.5020	116.00	2	24.8	84.2	0	158-171	K.FNWYVDGVEVHNAK.T	
165	1	603.7300	102.12	3	31.0	67.1	0	185-200	R.VVSVLTVLHQDWLNGK.E	Deamidated: 14
153	1	743.8160	108.48	3	29.2	83.6	1	185-203	R.VVSVLTVLHQDWLNGKEYK.C	Deamidated: 14
81	1	643.9020	95.36	2	22.2	44.2	0	228-238	R.EPQVYTLPPSR.D	
86	2	937.0670	83.54	2	22.6	39.7	1	228-243	R.EPQVYTLPPSRDELTK.N	
101	1	581.3890	121.36	2	23.8	73.7	0	244-253	K.NQVSLTCLVK.G	Carbamidomethyl: 7
158	2	1272.6020	25.68	2	29.8	92.1	0	254-275	K.GFYPSDIAVEWESNGQPENNYK.T	
161	2	1273.5730	401.92	2	30.2	112.1	0	254-275	K.GFYPSDIAVEWESNGQPENNYK.T	Deamidated: 14
163	2	1273.0920	24.09	2	30.6	71.1	0	254-275	K.GFYPSDIAVEWESNGQPENNYK.T	Deamidated: 19
162	1	937.5470	87.95	2	30.5	63.2	0	276-292	K.TTPPVLDSDGSFFLYSK.L	
98	2	934.5060	84.31	3	23.6	56.7	0	300-322	R.WQQGNVFSCSVMHEALHNHYTQK.S	Carbamidomethyl: 9
77	6	939.8290	74.64	3	21.9	61.8	0	300-322	R.WQQGNVFSCSVMHEALHNHYTQK.S	Carbamidomethyl: 9; Oxidation: 12

Protein 2: Ig kappa chain C region OS=Homo sapiens GN=IGKC PE=1 SV=1

Accession: IGKC_HUMAN
Database: SwissProt_July2013
Seq. Coverage [%]: 90.60 %

Score: 922.67
MW [kDa]: 11.60
pI: 5.53
No. of Peptides: 12

Modification(s): Carbamidomethyl, Deamidated

Cmpd.	No. of Cmpds.	m/z meas.	Δ m/z [ppm]	z	Rt [min]	Score	P	Range	Sequence	Modification
166	2	973.6020	87.19	2	31.1	39.5	0	1-18	-.TVAAPSVFIFPPSDEQLK.S	
174	1	899.5300	87.55	2	32.2	64.6	0	19-34	K.SGTASVVCLLNNFYPR.E	Carbamidomethyl: 8
92	1	893.4620	43.82	3	23.2	95.3	1	38-61	K.VQWKVDNALQSGNSQESVTEQDSK.D	Deamidated: 13
83	3	893.1390	49.44	3	22.4	97.2	1	38-61	K.VQWKVDNALQSGNSQESVTEQDSK.D	
127	1	1041.0700	59.46	4	26.8	32.1	2	38-75	K.VQWKVDNALQSGNSQESVTEQDSKDSTYSLSSTLTLSK.A	
49	2	1068.9440	-33.69	2	19.1	85.8	0	42-61	K.VDNALQSGNSQESVTEQDSK.D	Deamidated: 3

50	3	1068.9750	455.78	2	19.2	126.8	0	42-61	K.VDNALQSGNSQESVTEQDSK.D	
125	2	1207.2760	28.75	3	26.7	129.7	1	42-75	K.VDNALQSGNSQESVTEQDSKDST YSLSSTLTLSK.A	
129	1	751.9650	109.24	2	27.0	113.5	0	62-75	K.DSTYSLSSTLTLSK.A	
1	1	445.7480	58.41	2	14.5	27.5	1	76-82	K.ADYEKHK.V	
44	1	714.4400	104.82	3	18.6	107.8	1	81-99	K.HKVYACEVTHQGLSSPVTK.S	Carbamidomethyl: 6
60	1	938.5480	86.20	2	20.4	88.8	0	83-99	K.VYACEVTHQGLSSPVTK.S	Carbamidomethyl: 4

Protein 3: Ig gamma-4 chain C region OS=Homo sapiens GN=IGHG4 PE=1 SV=1
Accession: IGHG4_HUMAN
Database: SwissProt_July2013
Seq. Coverage [%]: 20.50 %
Score: 643.15
MW [kDa]: 35.90
pI: 7.80
No. of Peptides: 4
Modification(s): Carbamidomethyl, Deamidated

Cmpd.	No. of Cmpds.	m/z meas.	Δ m/z [ppm]	z	Rt [min]	Score	P	Range	Sequence	Modification
118	1	644.3970	105.00	2	25.5	53.3	0	5-16	K.GPSVFPLAPCSR.S	Carbamidomethyl: 10
94	1	712.4270	96.16	2	23.3	70.5	0	17-30	R.STSESTAALGCLVK.D	Carbamidomethyl: 11
3	1	555.6460	92.24	3	14.7	43.3	0	80-93	K.TYTCNVDHKPSNTK.V	Carbamidomethyl: 4
172	1	981.9070	84.62	3	31.8	47.5	0	102-128	K.YGPPCPSCPAPEFLGGPSVFLFP PKPK.D	Carbamidomethyl: 5, 8

Protein 4: Ig gamma-2 chain C region OS=Homo sapiens GN=IGHG2 PE=1 SV=2
Accession: IGHG2_HUMAN
Database: SwissProt_July2013
Seq. Coverage [%]: 23.90 %
Score: 626.73
MW [kDa]: 35.90
pI: 8.80
No. of Peptides: 5
Modification(s): Carbamidomethyl, Oxidation, Deamidated

Cmpd.	No. of Cmpds.	m/z meas.	Δ m/z [ppm]	z	Rt [min]	Score	P	Range	Sequence	Modification
154	2	970.2000	63.14	3	29.3	42.6	0	102-127	K.CCVECPPCPAPPVAGPSVFLFPP KPK.D	Carbamidomethyl: 1, 2, 5, 8
134	1	739.1480	114.42	3	27.6	100.1	1	181-199	R.VVSVLTVVHQDWLNGKEYK.C	Deamidated: 14
75	1	635.7080	87.68	3	21.7	35.8	1	224-239	R.EPQVYTLPPSREEMTK.N	
66	3	961.0500	80.94	2	20.8	45.2	1	224-239	R.EPQVYTLPPSREEMTK.N	Oxidation: 14

155	2	961.5010	55.08	2	29.3	42.8	0	272-288	K.TTPPMLDSDGSFFLYSK.L	Oxidation: 5

Protein 5: Ig lambda-3 chain C regions OS=Homo sapiens GN=IGLC3 PE=1 SV=1
Accession: LAC3_HUMAN
Database: SwissProt_July2013
Seq. Coverage [%]: 67.90 %
Score: 389.25
MW [kDa]: 11.20
pI: 7.72
No. of Peptides: 6
Modification(s): Carbamidomethyl

Cmpd.	No. of Cmpds.	m/z meas.	Δ m/z [ppm]	z	Rt [min]	Score	P	Range	Sequence	Modification
132	1	993.5770	64.86	2	27.4	67.4	0	5-23	K.AAPSVTLFPPSSEELQANK.A	
8	1	495.8100	103.38	2	15.3	69.6	0	51-60	K.AGVETTTPSK.Q	
123	1	772.4390	74.31	3	25.7	24.0	1	61-80	K.QSNNKYAASSYLSLTPEQWK.S	
145	1	872.5190	98.52	2	28.5	67.9	0	66-80	K.YAASSYLSLTPEQWK.S	
30	1	856.4710	102.49	2	17.4	109.8	0	84-98	K.SYSCQVTHEGSTVEK.T	Carbamidomethyl: 4
63	1	853.0690	29.05	3	20.6	50.6	1	84-106	K.SYSCQVTHEGSTVEKTVAPTECS .-	Carbamidomethyl: 4, 22

Protein 6: Ig kappa chain V-III region VG (Fragment) OS=Homo sapiens PE=1 SV=1
Accession: KV309_HUMAN
Database: SwissProt_July2013
Seq. Coverage [%]: 18.30 %
Score: 57.62
MW [kDa]: 12.60
pI: 4.69
No. of Peptides: 1

Cmpd.	No. of Cmpds.	m/z meas.	Δ m/z [ppm]	z	Rt [min]	Score	P	Range	Sequence	Modification
115	1	784.8130	106.76	3	25.2	57.6	0	45-65	R.ASQSVSSYLAWYQQKPGQAPR.L	

Protein 7: Ig kappa chain V-II region MIL OS=Homo sapiens PE=1 SV=1
Accession: KV203_HUMAN
Database: SwissProt_July2013
Seq. Coverage [%]: 17.90 %
Score: 52.77
MW [kDa]: 12.00
pI: 0.00
No. of Peptides: 1
Modification(s): Deamidated

Cmpd.	No. of Cmpds.	m/z meas.	Δ m/z [ppm]	z	Rt [min]	Score	P	Range	Sequence	Modification
95	1	662.7000	64.96	3	23.3	52.8	1	59-78	R.ASGVPNRFSGSGSGTDFTLK.I	Deamidated: 6

Protein 8: Ig lambda chain V-IV region MOL OS=Homo sapiens PE=1 SV=1
Accession: LV405_HUMAN
Database: SwissProt_July2013
Seq. Coverage [%]: 7.50 %
Score: 41.60
MW [kDa]: 11.30
pI: 4.28
No. of Peptides: 1

Cmpd.	No. of Cmpds.	m/z meas.	Δ m/z [ppm]	z	Rt [min]	Score	P	Range	Sequence	Modification
13	1	456.3120	121.62	2	15.6	41.6	0	52-59	K.RPSGIPER.F	

Search Type Combined MS/MS - ProteinExtractor
Search Result Amazon Human_SPJuly2013_2016-08-08 18:23:29
Search Location /Kathir/ABC 2016 IgG And A1AT/KA IgG ISo Drop 1min E2_RC4_01_8273.d/

Protein 1: Ig gamma-1 chain C region OS=Homo sapiens GN=IGHG1 PE=1 SV=1
Accession: IGHG1_HUMAN
Database: SwissProt_July2013
Seq. Coverage [%]: 50.30 %
Score: 1002.58
MW [kDa]: 36.10
pI: 9.36
No. of Peptides: 17

Modification(s): Carbamidomethyl, Oxidation, Deamidated

Cmpd.	No. of Cmpds.	m/z meas.	Δ m/z [ppm]	z	Rt [min]	Score	P	Range	Sequence	Modification
125	1	593.8940	112.89	2	24.6	57.5	0	5-16	K.GPSVFPLAPSSK.S	
101	1	661.4130	106.37	2	22.5	94.9	0	17-30	K.STSGGTAALGCLVK.D	Carbamidomethyl: 11
133	2	1070.6220	565.08	2	25.3	65.3	0	139-157	R.TPEVTCVVVDVSHEDPEVK.F	Carbamidomethyl: 6
127	1	839.5070	121.96	2	24.8	86.5	0	158-171	K.FNWYVDGVEVHNAK.T	
178	1	743.8210	115.21	3	29.2	100.8	1	185-203	R.VVSVLTVLHQDWLNGKEYK.C	Deamidated: 14

97	2	643.9010	93.81	2	22.2	31.7	0	228-238	R.EPQVYTLPPSR.D	
103	2	937.0820	99.55	2	22.7	31.3	1	228-243	R.EPQVYTLPPSRDELTK.N	
118	1	581.3890	121.36	2	23.8	79.5	0	244-253	K.NQVSLTCLVK.G	Carbamidomethyl: 7
184	4	1272.6150	35.89	2	29.8	97.5	0	254-275	K.GFYPSDIAVEWESNGQPENNYK.T	
187	4	1273.5670	397.21	2	30.2	107.6	0	254-275	K.GFYPSDIAVEWESNGQPENNYK.T	Deamidated: 14
189	4	1273.1010	31.16	2	30.6	80.3	0	254-275	K.GFYPSDIAVEWESNGQPENNYK.T	Deamidated: 16
199	3	1273.6040	39.78	2	32.0	47.9	0	254-275	K.GFYPSDIAVEWESNGQPENNYK.T	Deamidated: 14, 19
198	3	1274.0740	408.82	2	31.8	40.4	0	254-275	K.GFYPSDIAVEWESNGQPENNYK.T	Deamidated: 16, 20
188	1	937.5580	99.68	2	30.5	67.0	0	276-292	K.TTPPVLDSDGSFFLYSK.L	
124	1	935.4640	407.20	3	24.4	31.4	0	300-322	R.WQQGNVFSCSVMHEALHNHYTQK.S	Carbamidomethyl: 9; Deamidated: 2, 3
121	2	934.5200	99.29	3	24.1	57.5	0	300-322	R.WQQGNVFSCSVMHEALHNHYTQK.S	Carbamidomethyl: 9
89	2	939.8460	92.73	3	21.5	53.8	0	300-322	R.WQQGNVFSCSVMHEALHNHYTQK.S	Carbamidomethyl: 9; Oxidation: 12

Protein 2: Ig kappa chain C region OS=Homo sapiens GN=IGKC PE=1 SV=1

Accession: IGKC_HUMAN
Database: SwissProt_July2013
Seq. Coverage [%]: 90.60 %

Score: 743.69
MW [kDa]: 11.60
pI: 5.53
No. of Peptides: 11

Modification(s): Carbamidomethyl, Deamidated

Cmpd.	No. of Cmpds.	m/z meas.	Δ m/z [ppm]	z	Rt [min]	Score	P	Range	Sequence	Modification
190	1	973.6160	101.57	2	30.9	96.5	0	1-18	-.TVAAPSVFIFPPSDEQLK.S	
200	1	899.5380	96.44	2	32.2	63.8	0	19-34	K.SGTASVVCLLNNFYPR.E	Carbamidomethyl: 8
100	1	893.1300	39.36	3	22.5	88.7	1	38-61	K.VQWKVDNALQSGNSQESVTEQDSK.D	
70	3	1068.5240	33.69	2	19.2	129.0	0	42-61	K.VDNALQSGNSQESVTEQDSK.D	
69	1	1068.9470	-30.88	2	19.1	82.4	0	42-61	K.VDNALQSGNSQESVTEQDSK.D	Deamidated: 3
132	1	1069.6170	135.57	2	25.2	20.1	0	42-61	K.VDNALQSGNSQESVTEQDSK.D	Deamidated: 3, 9
147	1	1207.6090	32.88	3	26.8	108.3	1	42-75	K.VDNALQSGNSQESVTEQDSKDST	Deamidated: 3

									YSLSSTLTLSK.A	
135	3	1207.2680	22.12	3	25.6	133.1	1	42-75	K.VDNALQSGNSQESVTEQDSKDST YSLSSTLTLSK.A	
15	1	445.7830	136.93	2	14.4	26.0	1	76-82	K.ADYEKHK.V	
67	1	1071.1460	95.20	2	18.6	109.8	1	81-99	K.HKVYACEVTHQGLSSPVTK.S	Carbamidomethyl: 6
82	1	626.0340	85.48	3	20.4	76.8	0	83-99	K.VYACEVTHQGLSSPVTK.S	Carbamidomethyl: 4

Protein 3: Ig gamma-4 chain C region OS=Homo sapiens GN=IGHG4 PE=1 SV=1
Accession: IGHG4_HUMAN **Score:** 702.89
Database: SwissProt_July2013 **MW [kDa]:** 35.90
Seq. Coverage [%]: 20.50 % **pI:** 7.80
No. of Peptides: 4
Modification(s): Carbamidomethyl, Deamidated

Cmpd.	No. of Cmpds.	m/z meas.	Δ m/z [ppm]	z	Rt [min]	Score	P	Range	Sequence	Modification
134	1	644.4010	111.20	2	25.5	58.3	0	5-16	K.GPSVFPLAPCSR.S	Carbamidomethyl: 10
110	1	712.4370	110.20	2	23.2	78.8	0	17-30	R.STSESTAALGCLVK.D	Carbamidomethyl: 11
20	1	832.9660	93.07	2	14.7	61.9	0	80-93	K.TYTCNVDHKPSNTK.V	Carbamidomethyl: 4
195	1	981.9210	98.88	3	31.6	46.8	0	102-128	K.YGPPCPSCPAPEFLGGPSVFLFP PKPK.D	Carbamidomethyl: 5, 8

Protein 4: Ig gamma-2 chain C region OS=Homo sapiens GN=IGHG2 PE=1 SV=2
Accession: IGHG2_HUMAN **Score:** 597.58
Database: SwissProt_July2013 **MW [kDa]:** 35.90
Seq. Coverage [%]: 18.10 % **pI:** 8.80
No. of Peptides: 4
Modification(s): Carbamidomethyl, Oxidation, Deamidated

Cmpd.	No. of Cmpds.	m/z meas.	Δ m/z [ppm]	z	Rt [min]	Score	P	Range	Sequence	Modification
180	4	970.2340	98.19	3	29.3	66.7	0	102-127	K.CCVECPPCPAPPVAGPSVFLFPP KPK.D	Carbamidomethyl: 1, 2, 5, 8
79	2	961.0510	81.98	2	20.3	53.6	1	224-239	R.EPQVYTLPPSREEMTK.N	Oxidation: 14
94	1	953.0650	94.69	2	21.8	35.0	1	224-239	R.EPQVYTLPPSREEMTK.N	
182	2	962.0350	610.49	2	29.6	51.6	0	272-288	K.TTPPMLDSDGSFFLYSK.L	Oxidation: 5

Protein 5: Ig lambda-3 chain C regions OS=Homo sapiens GN=IGLC3 PE=1 SV=1
Accession: LAC3_HUMAN
Database: SwissProt_July2013
Seq. Coverage [%]: 63.20 %
Score: 380.95
MW [kDa]: 11.20
pI: 7.72
No. of Peptides: 5
Modification(s): Carbamidomethyl

Cmpd.	No. of Cmpds.	m/z meas.	Δ m/z [ppm]	z	Rt [min]	Score	P	Range	Sequence	Modification
156	2	993.6020	90.02	2	27.4	84.4	0	5-23	K.AAPSVTLFPPSSEELQANK.A	
26	1	495.8110	105.40	2	15.3	59.9	0	51-60	K.AGVETTTPSK.Q	
171	1	872.5250	105.39	2	28.5	94.7	0	66-80	K.YAASSYLSLTPEQWK.S	
45	1	856.4460	73.29	2	16.8	103.5	0	84-98	K.SYSCQVTHEGSTVEK.T	Carbamidomethyl: 4
83	1	853.0690	29.05	3	20.7	38.5	1	84-106	K.SYSCQVTHEGSTVEKTVAPTECS.-	Carbamidomethyl: 4, 22

Protein 6: Immunoglobulin lambda-like polypeptide 5 OS=Homo sapiens GN=IGLL5 PE=2 SV=2
Accession: IGLL5_HUMAN
Database: SwissProt_July2013
Seq. Coverage [%]: 13.60 %
Score: 358.85
MW [kDa]: 23.00
pI: 10.03
No. of Peptides: 2
Modification(s): Carbamidomethyl

Cmpd.	No. of Cmpds.	m/z meas.	Δ m/z [ppm]	z	Rt [min]	Score	P	Range	Sequence	Modification
155	1	1022.1010	76.03	2	27.3	64.4	0	113-131	K.ANPTVTLFPPSSEELQANK.A	
18	1	509.3450	122.94	2	14.5	57.7	0	159-168	K.AGVETTKPSK.Q	

Protein 7: Ig kappa chain V-II region MIL OS=Homo sapiens PE=1 SV=1
Accession: KV203_HUMAN
Database: SwissProt_July2013
Seq. Coverage [%]: 17.90 %
Score: 64.46
MW [kDa]: 12.00
pI: 0.00
No. of Peptides: 1
Modification(s): Deamidated

Accumulated Protein Report

Cmpd.	No. of Cmpds.	m/z meas.	Δ m/z [ppm]	z	Rt [min]	Score	P	Range	Sequence	Modification
112	1	662.7170	90.61	3	23.3	64.5	1	59-78	R.ASGVPNRFSGSGSGTDFTLK.I	Deamidated: 6

Protein 8: Ig lambda chain V-IV region MOL OS=Homo sapiens PE=1 SV=1
Accession: LV405_HUMAN
Database: SwissProt_July2013
Seq. Coverage [%]: 7.50 %
Score: 37.28
MW [kDa]: 11.30
pI: 4.28
No. of Peptides: 1

Cmpd.	No. of Cmpds.	m/z meas.	Δ m/z [ppm]	z	Rt [min]	Score	P	Range	Sequence	Modification
32	1	456.3200	139.16	2	15.6	37.3	0	52-59	K.RPSGIPER.F	

Search Type Combined MS/MS - ProteinExtractor
Search Result Amazon Human_SPJuly2013_2016-08-08 18:23:29
Search Location /Kathir/ABC 2016 IgG And A1AT/KA IgG ISo Drop 1min E2_RC4_01_8298.d/

Protein 1: Ig gamma-1 chain C region OS=Homo sapiens GN=IGHG1 PE=1 SV=1
Accession: IGHG1_HUMAN
Database: SwissProt_July2013
Seq. Coverage [%]: 53.60 %
Score: 1081.01
MW [kDa]: 36.10
pI: 9.36
No. of Peptides: 16

Modification(s): Carbamidomethyl, Oxidation, Deamidated

Cmpd.	No. of Cmpds.	m/z meas.	Δ m/z [ppm]	z	Rt [min]	Score	P	Range	Sequence	Modification
107	1	593.8990	121.31	2	24.6	69.2	0	5-16	K.GPSVFPLAPSSK.S	
86	1	661.4110	103.35	2	22.6	101.5	0	17-30	K.STSGGTAALGCLVK.D	Carbamidomethyl: 11
131	2	1112.5700	315.68	3	27.7	81.6	1	102-131	K.SCDKTHTCPPCPAPELLGGPSVF LFPPKPK.D	Carbamidomethyl: 2, 8, 11
144	2	948.9210	102.20	3	28.7	66.1	0	106-131	K.THTCPPCPAPELLGGPSVFLFPP KPK.D	Carbamidomethyl: 4, 7

111	1	1070.1140	90.32	2	25.2	103.8	0	139-157	R.TPEVTCVVVDVSHEDPEVK.F	Carbamidomethyl: 6
109	1	839.5080	123.15	2	24.8	85.5	0	158-171	K.FNWYVDGVEVHNAK.T	
88	1	937.0620	78.20	2	22.7	34.1	1	228-243	R.EPQVYTLPPSRDELTK.N	
100	1	581.4030	145.45	2	23.8	71.6	0	244-253	K.NQVSLTCLVK.G	Carbamidomethyl: 7
153	4	1272.6070	29.61	2	29.8	93.1	0	254-275	K.GFYPSDIAVEWESNGQPENNYK.T	
157	7	1273.5860	412.13	2	30.2	102.3	0	254-275	K.GFYPSDIAVEWESNGQPENNYK.T	Deamidated: 14
165	7	1273.0750	10.74	2	31.5	90.7	0	254-275	K.GFYPSDIAVEWESNGQPENNYK.T	Deamidated: 16
161	4	1273.5900	28.78	2	30.8	93.2	0	254-275	K.GFYPSDIAVEWESNGQPENNYK.T	Deamidated: 14, 20
160	4	1273.5840	24.07	2	30.7	105.6	0	254-275	K.GFYPSDIAVEWESNGQPENNYK.T	Deamidated: 14, 19
158	1	937.5470	87.95	2	30.5	72.0	0	276-292	K.TTPPVLDSDGSFFLYSK.L	
69	2	939.8430	89.53	3	21.3	40.2	0	300-322	R.WQQGNVFSCSVMHEALHNHYTQK.S	Carbamidomethyl: 9; Oxidation: 12
97	1	934.5160	95.01	3	23.6	54.5	0	300-322	R.WQQGNVFSCSVMHEALHNHYTQK.S	Carbamidomethyl: 9

Protein 2: Ig kappa chain C region OS=Homo sapiens GN=IGKC PE=1 SV=1

Accession: IGKC_HUMAN — **Score:** 774.49

Database: SwissProt_July2013 — **MW [kDa]:** 11.60

Seq. Coverage [%]: 85.80 % — **pI:** 5.53

No. of Peptides: 9

Modification(s): Carbamidomethyl

Cmpd.	No. of Cmpds.	m/z meas.	Δ m/z [ppm]	z	Rt [min]	Score	P	Range	Sequence	Modification
164	1	973.5990	84.11	2	31.0	68.9	0	1-18	-.TVAAPSVFIFPPSDEQLK.S	
168	1	899.5390	97.56	2	32.3	60.0	0	19-34	K.SGTASVVCLLNNFYPR.E	Carbamidomethyl: 8
85	1	893.1290	38.24	3	22.5	78.4	1	38-61	K.VQWKVDNALQSGNSQESVTEQDSK.D	
121	1	1041.0690	58.49	4	26.9	23.9	2	38-75	K.VQWKVDNALQSGNSQESVTEQDSKDSTYSLSSTLTLSK.A	
50	2	1068.5440	52.41	2	19.2	129.6	0	42-61	K.VDNALQSGNSQESVTEQDSK.D	
119	2	1207.2720	25.43	3	26.8	126.3	1	42-75	K.VDNALQSGNSQESVTEQDSKDSTYSLSSTLTLSK.A	

124	1	751.9520	91.95	2	27.1	77.9	0	62-75	K.DSTYSLSSTLTLSK.A	
40	2	1071.1610	109.20	2	18.4	105.4	1	81-99	K.HKVYACEVTHQGLSSPVTK.S	Carbamidomethyl: 6
60	1	938.5530	91.53	2	20.4	88.7	0	83-99	K.VYACEVTHQGLSSPVTK.S	Carbamidomethyl: 4

Protein 3: Ig gamma-4 chain C region OS=Homo sapiens GN=IGHG4 PE=1 SV=1

Accession: IGHG4_HUMAN

Database: SwissProt_July2013

Seq. Coverage [%]: 20.50 %

Score: 592.00

MW [kDa]: 35.90

pI: 7.80

No. of Peptides: 4

Modification(s): Carbamidomethyl, Deamidated

Cmpd.	No. of Cmpds.	m/z meas.	Δ m/z [ppm]	z	Rt [min]	Score	P	Range	Sequence	Modification
113	1	644.4090	123.62	2	25.5	56.5	0	5-16	K.GPSVFPLAPCSR.S	Carbamidomethyl: 10
94	1	712.4250	93.35	2	23.3	67.9	0	17-30	R.STSESTAALGCLVK.D	Carbamidomethyl: 11
2	1	555.6480	95.84	3	14.7	40.8	0	80-93	K.TYTCNVDHKPSNTK.V	Carbamidomethyl: 4
167	1	981.8990	76.47	3	31.8	54.2	0	102-128	K.YGPPCPSCPAPEFLGGPSVFLFP PKPK.D	Carbamidomethyl: 5, 8

Protein 4: Ig gamma-2 chain C region OS=Homo sapiens GN=IGHG2 PE=1 SV=2

Accession: IGHG2_HUMAN

Database: SwissProt_July2013

Seq. Coverage [%]: 18.40 %

Score: 530.65

MW [kDa]: 35.90

pI: 8.80

No. of Peptides: 5

Modification(s): Carbamidomethyl, Oxidation, Deamidated

Cmpd.	No. of Cmpds.	m/z meas.	Δ m/z [ppm]	z	Rt [min]	Score	P	Range	Sequence	Modification
122	1	759.9490	91.31	4	27.0	27.1	1	101-127	R.KCCVECPPCPAPPVAGPSVFLFP PKPK.D	Carbamidomethyl: 2, 3, 6, 9
149	3	970.5080	380.62	3	29.2	54.4	0	102-127	K.CCVECPPCPAPPVAGPSVFLFPP KPK.D	Carbamidomethyl: 1, 2, 5, 8
59	4	961.0600	91.35	2	20.2	50.5	1	224-239	R.EPQVYTLPPSREEMTK.N	Oxidation: 14
78	1	953.0500	78.95	2	21.9	44.8	1	224-239	R.EPQVYTLPPSREEMTK.N	
151	1	961.5180	72.76	2	29.5	43.5	0	272-288	K.TTPPMLDSDGSFFLYSK.L	Oxidation: 5

Protein 5: Ig lambda-3 chain C regions OS=Homo sapiens GN=IGLC3 PE=1 SV=1

Accumulated Protein Report

Accession: LAC3_HUMAN
Database: SwissProt_July2013
Seq. Coverage [%]: 60.40 %
Score: 327.71
MW [kDa]: 11.20
pI: 7.72
No. of Peptides: 5

Modification(s): Carbamidomethyl

Cmpd.	No. of Cmpds.	m/z meas.	Δ m/z [ppm]	z	Rt [min]	Score	P	Range	Sequence	Modification
126	1	993.5730	60.83	2	27.3	75.3	0	5-23	K.AAPSVTLFPPSSEELQANK.A	
9	1	495.8120	107.42	2	15.4	81.9	0	51-60	K.AGVETTTPSK.Q	
116	1	772.4520	91.14	3	25.8	45.6	1	61-80	K.QSNNKYAASSYLSLTPEQWK.S	
142	1	872.5100	88.20	2	28.5	53.1	0	66-80	K.YAASSYLSLTPEQWK.S	
23	2	856.4860	120.00	2	16.8	71.8	0	84-98	K.SYSCQVTHEGSTVEK.T	Carbamidomethyl: 4

Protein 6: Ig kappa chain V-II region MIL OS=Homo sapiens PE=1 SV=1
Accession: KV203_HUMAN
Database: SwissProt_July2013
Seq. Coverage [%]: 17.90 %
Score: 56.75
MW [kDa]: 12.00
pI: 0.00
No. of Peptides: 1

Modification(s): Deamidated

Cmpd.	No. of Cmpds.	m/z meas.	Δ m/z [ppm]	z	Rt [min]	Score	P	Range	Sequence	Modification
95	1	662.7050	72.50	3	23.3	56.8	1	59-78	R.ASGVPNRFSGSGSGTDFTLK.I	Deamidated: 6

Search Type Combined MS/MS - ProteinExtractor
Search Result Amazon Human_SPJuly2013_2016-08-08 18:23:29
Search Location /Kathir/ABC 2016 IgG And A1AT/KA IgG ISo Drop 1min E3_RC5_01_8274.d/

Protein 1: Ig gamma-1 chain C region OS=Homo sapiens GN=IGHG1 PE=1 SV=1
Accession: IGHG1_HUMAN
Database: SwissProt_July2013
Seq. Coverage [%]: 54.50 %
Score: 1244.02
MW [kDa]: 36.10
pI: 9.36

Accumulated Protein Report

No. of Peptides: 18

Modification(s): Carbamidomethyl, Oxidation, Deamidated

Cmpd.	No. of Cmpds.	m/z meas.	Δ m/z [ppm]	z	Rt [min]	Score	P	Range	Sequence	Modification
126	1	593.8890	104.47	2	24.6	72.2	0	5-16	K.GPSVFPLAPSSK.S	
99	1	661.4090	100.32	2	22.5	95.9	0	17-30	K.STSGGTAALGCLVK.D	Carbamidomethyl: 11
65	1	426.2730	128.57	2	19.3	44.6	0	132-138	K.DTLMISR.T	Oxidation: 4
132	1	714.0750	552.54	3	25.2	80.3	0	139-157	R.TPEVTCVVVDVSHEDPEVK.F	Carbamidomethyl: 6
128	1	839.5100	125.53	2	24.8	94.8	0	158-171	K.FNWYVDGVEVHNAK.T	
180	1	743.8050	93.69	3	29.3	96.0	1	185-203	R.VVSVLTVLHQDWLNGKEYK.C	Deamidated: 14
95	2	643.9020	95.36	2	22.2	41.1	0	228-238	R.EPQVYTLPPSR.D	
101	2	937.0740	91.01	2	22.8	50.9	1	228-243	R.EPQVYTLPPSRDELTK.N	
116	1	581.3930	128.25	2	23.7	76.3	0	244-253	K.NQVSLTCLVK.G	Carbamidomethyl: 7
186	4	1273.5630	394.06	2	30.3	118.2	0	254-275	K.GFYPSDIAVEWESNGQPENNYK.T	Deamidated: 14
188	4	1273.1010	31.16	2	30.7	68.1	0	254-275	K.GFYPSDIAVEWESNGQPENNYK.T	Deamidated: 19
189	3	1273.5940	31.93	2	30.7	96.0	0	254-275	K.GFYPSDIAVEWESNGQPENNYK.T	Deamidated: 14, 19
184	2	1272.6190	39.04	2	29.8	100.4	0	254-275	K.GFYPSDIAVEWESNGQPENNYK.T	
187	1	937.5590	100.75	2	30.5	80.6	0	276-292	K.TTPPVLDSDGSFFLYSK.L	
16	1	409.7980	141.16	2	14.7	26.1	1	293-299	K.LTVDKSR.W	
114	2	934.5100	88.59	3	23.6	87.1	0	300-322	R.WQQGNVFSCSVMHEALHNHYTQK.S	Carbamidomethyl: 9
118	1	934.8350	85.34	3	24.0	31.3	0	300-322	R.WQQGNVFSCSVMHEALHNHYTQK.S	Carbamidomethyl: 9; Deamidated: 18
80	2	939.8360	82.08	3	21.3	52.3	0	300-322	R.WQQGNVFSCSVMHEALHNHYTQK.S	Carbamidomethyl: 9; Oxidation: 12

Protein 2: Ig kappa chain C region OS=Homo sapiens GN=IGKC PE=1 SV=1
Accession: IGKC_HUMAN
Score: 832.44
Database: SwissProt_July2013
MW [kDa]: 11.60
Seq. Coverage [%]: 97.20 %
pI: 5.53
No. of Peptides: 9

Modification(s): Carbamidomethyl, Deamidated

Cmpd.	No. of Cmpds.	m/z meas.	Δ m/z [ppm]	z	Rt [min]	Score	P	Range	Sequence	Modification
190	1	973.6160	101.57	2	31.0	80.4	0	1-18	-.TVAAPSVFIFPPSDEQLK.S	
196	1	899.5470	106.45	2	32.2	66.1	0	19-34	K.SGTASVVCLLNNFYPR.E	Carbamidomethyl: 8
98	1	893.1270	36.00	3	22.5	102.0	1	38-61	K.VQWKVDNALQSGNSQESVTEQDS K.D	
63	3	1068.9150	399.63	2	19.0	138.6	0	42-61	K.VDNALQSGNSQESVTEQDSK.D	
147	1	1207.6040	28.74	3	26.8	111.3	1	42-75	K.VDNALQSGNSQESVTEQDSKDST YSLSSTLTLSK.A	Deamidated: 3
11	1	445.7960	166.10	2	14.4	32.9	1	76-82	K.ADYEKHK.V	
60	1	714.4340	96.42	3	18.7	107.9	1	81-99	K.HKVYACEVTHQGLSSPVTK.S	Carbamidomethyl: 6
76	1	938.5470	85.14	2	20.4	78.3	0	83-99	K.VYACEVTHQGLSSPVTK.S	Carbamidomethyl: 4
19	1	435.2500	155.91	2	15.1	27.3	1	100-106	K.SFNRGEC.-	Carbamidomethyl: 7

Protein 3: Ig gamma-2 chain C region OS=Homo sapiens GN=IGHG2 PE=1 SV=2
Accession: IGHG2_HUMAN **Score:** 778.42
Database: SwissProt_July2013 **MW [kDa]:** 35.90
Seq. Coverage [%]: 31.90 % **pI:** 8.80
No. of Peptides: 7
Modification(s): Carbamidomethyl, Oxidation, Deamidated

Cmpd.	No. of Cmpds.	m/z meas.	Δ m/z [ppm]	z	Rt [min]	Score	P	Range	Sequence	Modification
133	1	644.4160	134.48	2	25.5	61.1	0	5-16	K.GPSVFPLAPCSR.S	Carbamidomethyl: 10
109	1	712.4260	94.76	2	23.3	83.0	0	17-30	R.STSESTAALGCLVK.D	Carbamidomethyl: 11
179	5	970.2280	92.00	3	29.3	57.9	0	102-127	K.CCVECPPCPAPPVAGPSVFLFPP KPK.D	Carbamidomethyl: 1, 2, 5, 8
155	1	739.1500	117.13	3	27.5	78.6	1	181-199	R.VVSVLTVVHQDWLNGKEYK.C	Deamidated: 14
92	1	635.7020	78.24	3	21.9	46.1	1	224-239	R.EPQVYTLPPSREEMTK.N	
73	2	961.0400	70.54	2	20.3	44.3	1	224-239	R.EPQVYTLPPSREEMTK.N	Oxidation: 14
178	1	961.5300	85.24	2	29.2	48.6	0	272-288	K.TTPPMLDSDGSFFLYSK.L	Oxidation: 5

Protein 4: Ig gamma-4 chain C region OS=Homo sapiens GN=IGHG4 PE=1 SV=1
Accession: IGHG4_HUMAN **Score:** 763.61
Database: SwissProt_July2013 **MW [kDa]:** 35.90
Seq. Coverage [%]: 4.30 % **pI:** 7.80
No. of Peptides: 1

Modification(s): Carbamidomethyl, Oxidation, Deamidated

Cmpd.	No. of Cmpds.	m/z meas.	Δ m/z [ppm]	z	Rt [min]	Score	P	Range	Sequence	Modification
17	1	832.9670	94.27	2	14.7	43.4	0	80-93	K.TYTCNVDHKPSNTK.V	Carbamidomethyl: 4

Protein 5: Ig gamma-3 chain C region OS=Homo sapiens GN=IGHG3 PE=1 SV=2
Accession: IGHG3_HUMAN
Database: SwissProt_July2013
Seq. Coverage [%]: 3.20 %
Score: 576.10
MW [kDa]: 41.30
pI: 9.41
No. of Peptides: 1
Modification(s): Carbamidomethyl, Oxidation, Deamidated

Cmpd.	No. of Cmpds.	m/z meas.	Δ m/z [ppm]	z	Rt [min]	Score	P	Range	Sequence	Modification
89	1	472.9580	118.99	3	21.7	44.6	0	207-218	K.WYVDGVEVHNAK.T	

Protein 6: Ig lambda-3 chain C regions OS=Homo sapiens GN=IGLC3 PE=1 SV=1
Accession: LAC3_HUMAN
Database: SwissProt_July2013
Seq. Coverage [%]: 60.40 %
Score: 382.25
MW [kDa]: 11.20
pI: 7.72
No. of Peptides: 5
Modification(s): Carbamidomethyl

Cmpd.	No. of Cmpds.	m/z meas.	Δ m/z [ppm]	z	Rt [min]	Score	P	Range	Sequence	Modification
154	3	993.6010	89.02	2	27.4	79.0	0	5-23	K.AAPSVTLFPPSSEELQANK.A	
23	1	495.8120	107.42	2	15.5	69.6	0	51-60	K.AGVETTTPSK.Q	
136	2	772.4730	118.33	3	25.7	49.7	1	61-80	K.QSNNKYAASSYLSLTPEQWK.S	
170	1	872.5290	109.98	2	28.5	98.3	0	66-80	K.YAASSYLSLTPEQWK.S	
43	2	856.4710	102.49	2	17.3	85.8	0	84-98	K.SYSCQVTHEGSTVEK.T	Carbamidomethyl: 4

Protein 7: Ig lambda-1 chain C regions OS=Homo sapiens GN=IGLC1 PE=1 SV=1
Accession: LAC1_HUMAN
Database: SwissProt_July2013
Seq. Coverage [%]: 27.40 %
Score: 370.79
MW [kDa]: 11.30
pI: 9.16

**No. of Peptides:**	2
**Modification(s):**	Carbamidomethyl

Cmpd.	No. of Cmpds.	m/z meas.	Δ m/z [ppm]	z	Rt [min]	Score	P	Range	Sequence	Modification
153	1	1022.1000	75.05	2	27.3	76.6	0	5-23	K.ANPTVTLFPPSSEELQANK.A	
14	1	509.3510	134.72	2	14.6	60.4	0	51-60	K.AGVETTKPSK.Q	

**Protein 8:**	Ig heavy chain V-III region TEI OS=Homo sapiens PE=1 SV=1		
**Accession:**	HV316_HUMAN	**Score:**	110.60
**Database:**	SwissProt_July2013	**MW [kDa]:**	12.80
**Seq. Coverage [%]:**	16.00 %	**pI:**	9.57
		**No. of Peptides:**	1

Cmpd.	No. of Cmpds.	m/z meas.	Δ m/z [ppm]	z	Rt [min]	Score	P	Range	Sequence	Modification
141	1	941.5910	91.27	2	26.4	110.6	0	1-19	-.EVQLVESGGGLVQPGGSLR.L	

**Search Type**	Combined MS/MS - ProteinExtractor
**Search Result**	Amazon Human_SPJuly2013_2016-08-08 18:23:29
**Search Location**	/Kathir/ABC 2016 IgG And A1AT/KA IgG ACN Drop 1min E3_RA3_01_8262.d/

**Protein 1:**	Ig gamma-1 chain C region OS=Homo sapiens GN=IGHG1 PE=1 SV=1		
**Accession:**	IGHG1_HUMAN	**Score:**	934.19
**Database:**	SwissProt_July2013	**MW [kDa]:**	36.10
**Seq. Coverage [%]:**	53.60 %	**pI:**	9.36
		**No. of Peptides:**	14
**Modification(s):**	Carbamidomethyl, Deamidated		

Cmpd.	No. of Cmpds.	m/z meas.	Δ m/z [ppm]	z	Rt [min]	Score	P	Range	Sequence	Modification
144	1	593.8690	70.79	2	24.6	84.8	0	5-16	K.GPSVFPLAPSSK.S	

127	1	661.3930	76.13	2	22.5	89.2	0	17-30	K.STSGGTAALGCLVK.D	Carbamidomethyl: 11
166	2	1112.3030	75.62	3	27.7	62.7	1	102-131	K.SCDKTHTCPPCPAPELLGGPSVF LFPPKPK.D	Carbamidomethyl: 2, 8, 11
173	1	948.9210	102.20	3	28.7	58.6	0	106-131	K.THTCPPCPAPELLGGPSVFLFPP KPK.D	Carbamidomethyl: 4, 7
150	1	713.7270	64.93	3	25.1	52.8	0	139-157	R.TPEVTCVVVDVSHEDPEVK.F	Carbamidomethyl: 6
146	1	839.4730	81.45	2	24.8	90.9	0	158-171	K.FNWYVDGVEVHNAK.T	
124	2	643.8970	87.60	2	22.3	33.5	0	228-238	R.EPQVYTLPPSR.D	
130	1	937.0590	75.00	2	22.8	38.9	1	228-243	R.EPQVYTLPPSRDELTK.N	
139	1	581.3920	126.53	2	23.7	76.4	0	244-253	K.NQVSLTCLVK.G	Carbamidomethyl: 7
181	3	1272.5830	10.75	2	29.7	94.8	0	254-275	K.GFYPSDIAVEWESNGQPENNYK. T	
182	2	1273.0790	13.88	2	30.1	118.1	0	254-275	K.GFYPSDIAVEWESNGQPENNYK. T	Deamidated: 14
184	2	1273.0570	-3.40	2	30.6	72.3	0	254-275	K.GFYPSDIAVEWESNGQPENNYK. T	Deamidated: 19
183	1	937.5390	79.42	2	30.4	75.2	0	276-292	K.TTPPVLDSDGSFFLYSK.L	
135	1	934.5030	81.10	3	23.4	58.3	0	300-322	R.WQQGNVFSCSVMHEALHNHYTQK .S	Carbamidomethyl: 9

Protein 2: Ig kappa chain C region OS=Homo sapiens GN=IGKC PE=1 SV=1

Accession: IGKC_HUMAN

Database: SwissProt_July2013

Seq. Coverage [%]: 75.50 %

Score: 727.49

MW [kDa]: 11.60

pI: 5.53

No. of Peptides: 9

Modification(s): Carbamidomethyl, Deamidated

Cmpd.	No. of Cmpds.	m/z meas.	Δ m/z [ppm]	z	Rt [min]	Score	P	Range	Sequence	Modification
185	1	973.5920	76.92	2	30.9	72.6	0	1-18	-.TVAAPSVFIFPPSDEQLK.S	
125	1	893.1320	41.60	3	22.4	93.1	1	38-61	K.VQWKVDNALQSGNSQESVTEQDS K.D	
120	2	894.4760	77.29	3	21.8	33.6	1	38-61	K.VQWKVDNALQSGNSQESVTEQDS K.D	Deamidated: 2, 7, 13, 15
121	2	894.4670	434.12	3	21.9	59.5	1	38-61	K.VQWKVDNALQSGNSQESVTEQDS K.D	Deamidated: 2, 7, 10
95	3	1068.5650	72.06	2	19.2	123.4	0	42-61	K.VDNALQSGNSQESVTEQDSK.D	
156	2	1207.5610	264.82	3	26.1	140.1	1	42-75	K.VDNALQSGNSQESVTEQDSKDST	

									YSLSSTLTLSK.A	
34	1	445.7580	80.84	2	14.4	31.7	1	76-82	K.ADYEKHK.V	
85	1	1071.1490	98.00	2	18.2	124.6	1	81-99	K.HKVYACEVTHQGLSSPVTK.S	Carbamidomethyl: 6
105	1	938.5510	89.40	2	20.4	82.5	0	83-99	K.VYACEVTHQGLSSPVTK.S	Carbamidomethyl: 4

Protein 3: Ig gamma-2 chain C region OS=Homo sapiens GN=IGHG2 PE=1 SV=2

Accession: IGHG2_HUMAN
Database: SwissProt_July2013
Seq. Coverage [%]: 26.40 %

Score: 487.14
MW [kDa]: 35.90
pI: 8.80
No. of Peptides: 7

Modification(s): Carbamidomethyl, Oxidation

Cmpd.	No. of Cmpds.	m/z meas.	Δ m/z [ppm]	z	Rt [min]	Score	P	Range	Sequence	Modification
152	1	644.3920	97.24	2	25.4	59.9	0	5-16	K.GPSVFPLAPCSR.S	Carbamidomethyl: 10
133	1	712.4230	90.55	2	23.2	77.6	0	17-30	R.STSESTAALGCLVK.D	Carbamidomethyl: 11
160	1	759.9420	82.09	4	27.0	21.7	1	101-127	R.KCCVECPPCPAPPVAGPSVFLFP PKPK.D	Carbamidomethyl: 2, 3, 6, 9
177	2	970.2200	83.76	3	29.2	61.0	0	102-127	K.CCVECPPCPAPPVAGPSVFLFPP KPK.D	Carbamidomethyl: 1, 2, 5, 8
107	2	961.0480	78.86	2	20.6	35.0	1	224-239	R.EPQVYTLPPSREEMTK.N	Oxidation: 14
119	1	953.0550	84.20	2	21.7	30.7	1	224-239	R.EPQVYTLPPSREEMTK.N	
179	1	961.5180	72.76	2	29.4	33.0	0	272-288	K.TTPPMLDSDGSFFLYSK.L	Oxidation: 5

Protein 4: Ig lambda-2 chain C regions OS=Homo sapiens GN=IGLC2 PE=1 SV=1

Accession: LAC2_HUMAN
Database: SwissProt_July2013
Seq. Coverage [%]: 62.30 %

Score: 381.10
MW [kDa]: 11.30
pI: 7.73
No. of Peptides: 5

Modification(s): Carbamidomethyl

Cmpd.	No. of Cmpds.	m/z meas.	Δ m/z [ppm]	z	Rt [min]	Score	P	Range	Sequence	Modification
163	1	993.5850	72.91	2	27.3	91.3	0	5-23	K.AAPSVTLFPPSSEELQANK.A	
73	1	838.0020	84.88	2	17.3	43.5	1	44-60	K.ADSSPVKAGVETTTPSK.Q	
47	1	495.8050	93.30	2	15.3	67.4	0	51-60	K.AGVETTTPSK.Q	
170	1	872.5080	85.91	2	28.4	105.9	0	66-80	K.YAASSYLSLTPEQWK.S	

67	1	856.4460	73.29	2	16.7	72.9	0	84-98	R.SYSCQVTHEGSTVEK.T	Carbamidomethyl: 4

Protein 5: Ig gamma-3 chain C region OS=Homo sapiens GN=IGHG3 PE=1 SV=2
Accession: IGHG3_HUMAN
Database: SwissProt_July2013
Seq. Coverage [%]: 2.70 %
Score: 375.71
MW [kDa]: 41.30
pI: 9.41
No. of Peptides: 1
Modification(s): Carbamidomethyl, Oxidation

Cmpd.	No. of Cmpds.	m/z meas.	Δ m/z [ppm]	z	Rt [min]	Score	P	Range	Sequence	Modification
64	1	593.8060	89.91	2	16.5	51.0	0	119-128	K.SCDTPPPCPR.C	Carbamidomethyl: 2, 8

Protein 6: Ig lambda-1 chain C regions OS=Homo sapiens GN=IGLC1 PE=1 SV=1
Accession: LAC1_HUMAN
Database: SwissProt_July2013
Seq. Coverage [%]: 27.40 %
Score: 311.14
MW [kDa]: 11.30
pI: 9.16
No. of Peptides: 2
Modification(s): Carbamidomethyl

Cmpd.	No. of Cmpds.	m/z meas.	Δ m/z [ppm]	z	Rt [min]	Score	P	Range	Sequence	Modification
162	1	1022.0740	49.61	2	27.2	72.1	0	5-23	K.ANPTVTLFPPSSEELQANK.A	
37	1	509.3410	115.09	2	14.5	60.2	0	51-60	K.AGVETTKPSK.Q	

Protein 7: Ig lambda chain V-IV region Hil OS=Homo sapiens PE=1 SV=1
Accession: LV403_HUMAN
Database: SwissProt_July2013
Seq. Coverage [%]: 17.80 %
Score: 89.57
MW [kDa]: 11.50
pI: 6.93
No. of Peptides: 1

Cmpd.	No. of Cmpds.	m/z meas.	Δ m/z [ppm]	z	Rt [min]	Score	P	Range	Sequence	Modification
143	1	1002.5330	27.66	2	24.6	89.6	0	1-19	-.SYELTQPPSVSVSPGQTAR.I	

Accumulated Protein Report

Protein 8: Ig kappa chain V-III region NG9 (Fragment) OS=Homo sapiens PE=1 SV=1

**Accession:**	KV303_HUMAN	**Score:**	73.15
**Database:**	SwissProt_July2013	**MW [kDa]:**	10.70
**Seq. Coverage [%]:**	22.00 %	**pI:**	7.08
		**No. of Peptides:**	1

Cmpd.	No. of Cmpds.	m/z meas.	Δ m/z [ppm]	z	Rt [min]	Score	P	Range	Sequence	Modification
140	1	813.7830	52.96	3	24.0	73.2	0	29-50	R.ASQSVSSSYLAWYQQKPGQAPR.L	

Protein 9: Ig heavy chain V-III region HIL OS=Homo sapiens PE=1 SV=1

**Accession:**	HV310_HUMAN	**Score:**	53.08
**Database:**	SwissProt_July2013	**MW [kDa]:**	13.60
**Seq. Coverage [%]:**	13.20 %	**pI:**	9.93
		**No. of Peptides:**	1

Cmpd.	No. of Cmpds.	m/z meas.	Δ m/z [ppm]	z	Rt [min]	Score	P	Range	Sequence	Modification
169	1	796.9920	33.64	2	28.4	53.1	1	1-16	-.QVKLVQAGGGVVQPGR.S	
